# Effects of Various Feed Additives on Finishing Pig Growth Performance and Carcass Characteristics: A Review

**DOI:** 10.3390/ani13020200

**Published:** 2023-01-05

**Authors:** Zhong-Xing Rao, Mike D. Tokach, Jason C. Woodworth, Joel M. DeRouchey, Robert D. Goodband, Jordan T. Gebhardt

**Affiliations:** 1 Department of Animal Sciences and Industry, College of Agriculture, Kansas State University, Manhattan, KS 66506, USA; 2 Department of Diagnostic Medicine/Pathobiology, College of Veterinary Medicine, Kansas State University, Manhattan, KS 66506, USA

**Keywords:** carcass, feed additive, feed efficiency, finishing pig, swine

## Abstract

**Simple Summary:**

There are numerous feed additives that can be used to enhance grow–finish pig growth performance and carcass characteristics, which can potentially lead to a higher economic return. Therefore, we collected scientific data to summarize the results of these additives’ effects on grow–finish pigs. The feed additive categories chosen were acidifiers, betaine, chromium, conjugated linoleic acid, copper, direct-fed microbials, carbohydrases, proteases, phytases, multi-enzymes, essential oils, L-carnitine, yeasts, and zinc. We found that most of the results for each feed additive showed positive outcomes. For daily gain, direct-fed microbials, copper, L-carnitine, and multi-enzymes showed relatively large (>2.1% improvement) and positive effects. For feed efficiency, acidifiers, betaine, conjugated linoleic acid, multi-enzymes, direct-fed microbials, L-carnitine, and yeasts showed relatively large (>2.5% improvement) and positive effects. For backfat thickness, except for betaine, chromium, conjugated linoleic acid, and L-carnitine, most feed additives showed little effect (<1.7% improvement). This review provides information on the feed additives that have the most evidence in improving the performance of grow–finish pigs for pork producers around the world.

**Abstract:**

Feed additives have shown benefits throughout the literature in improving grow–finish pigs’ growth performance and carcass characteristics. However, the results have not been well summarized. Therefore, this review summarizes the available research (402 articles) on 14 feed additive categories fed to grow–finish pigs. The categories were acidifiers, betaine, Cr, conjugated linoleic acids, Cu, direct-fed microbials, carbohydrases, proteases, phytases, multi-enzymes, essential oils, L-carnitine, yeasts, and Zn. Qualified articles were collected and selected based on inclusion and exclusion criteria from online databases. The percentage difference for each response variable between the treatment and control group was calculated and summarized. Most results were positive for each feed additive; however, the magnitude of improvement varied, and most were not statistically significant. For ADG, DFM, Cu, L-carnitine, and multi-enzymes showed relatively large positive effects (>2.1% improvement) across a reasonable number of articles. Acidifiers, betaine, CLA, multi-enzymes, DFM, L-carnitine, and yeasts showed relatively large positive effects (>2.5% improvement) on improving G:F. Moreover, except for betaine, Cr, CLA, and L-carnitine, most feed additives showed little and non-significant effects on BF thickness (<1.7% improvement). This review provides a descriptive analysis for commonly used feed additives in the hope of better understanding feed additives’ effects on grow–finish pigs.

## 1. Introduction

Growth performance, especially feed efficiency, becomes increasingly important for achieving sustainable and competitive pig production as feed prices and environmental concerns rise. A pig consumes most of the feed in its lifetime during the grow–finish phase. Moreover, feed efficiency decreases as the pig’s weight increases because of the increasing maintenance requirement [1]. One of the potential methods to improve efficiency is including feed additives that have the potential to enhance energy utilization or reduce maintenance requirements.

Several feed additives have been widely used in the swine feed industry. These feed additives provide different mechanisms of action that can potentially improve growth performance without negatively affecting the ADG. Feed additives (acidifiers, EO, DFM, yeasts, Cu, and Zn) that show antibacterial and immune-promoting properties have been added to diets to control pathogens and maintain a balanced microbiota in the gastrointestinal tract [2,3,4,5,6,7]. Betaine, Cr, CLA, and L-carnitine are added to diets for their beneficial effects on energy and lipid metabolism [8,9,10,11]. Moreover, exogenous enzymes, such as carbohydrases, proteases, and phytases, are added to improve nutrient digestibility coefficients and for the potential positive effects on gastrointestinal health and immune functions [12,13]. Even though the mechanisms of these feed additives seem promising, the effects on finishing pigs’ growth performance are variable throughout the literature. This variability in response may be caused by the developmental status (weaned pigs vs. grow–finish pigs) of the pig, diet compositions [14,15], and environmental factors [16]. Moreover, because carcass characteristics are important economic criteria, this literature review also discusses the effects of these feed additives on backfat, percentage lean, and loin muscle criteria. Therefore, this literature review summarizes the available results of feed additive research to help determine which feed additives have the greatest and most consistent potential to improve growth performance and carcass characteristics of finishing pigs.

## 2. Materials and Methods

### 2.1. Data Source

The online article databases used for this literature review were the International System for Agricultural Science and Technology (AGRIS), Centre for Agriculture and Bioscience International (CABI; CAB Direct), Pork Checkoff research, PubMed, and Scopus. Articles were identified using the following terms: pig, swine, barrow, or gilt with the name of the feed additive of interest. The feed additives of interest were acidifiers, betaine, Cr, conjugated linoleic acid (CLA), Cu, direct-fed microbials (DFM), carbohydrases, proteases, phytases, multi-enzymes (combinations of carbohydrases, proteases, or phytases), essential oils (EO), L-carnitine, yeasts, and Zn. This literature review did not include ractopamine (RAC) because of the global trend of removing RAC in grow–finish pig diets. The language of the articles was limited to English, and the article types were limited to research articles and university research reports. There is no restriction on the publication years for selecting articles and the years of the selected papers ranged from 1957 to 2022.

### 2.2. Inclusion and Exclusion Criteria

Research articles were included if they met the following criteria: (1) the study was an original randomized controlled in vivo study; (2) the study had a control group fed a basal diet without the feed additive of interest and treatment groups with the feed additive added to the basal diet with other nutrient values similar to the control; (3) control and treatment pigs had to have a similar starting live body weight over 7 kg (post-weaning), and an end point above 80 kg live body weight with an identical (fix-time study) or similar (fix-weight study) experimental period; (4) the study reported either the growth performance (BW, ADG, ADFI, G:F), carcass characteristics [e.g., carcass weight, percent carcass yield, backfat thickness (BF), loin muscle area (LMA), loin muscle depth (LD), percentage lean] or both criteria with statistical analysis. The exclusion criteria were (1) duplicate search results; (2) data duplication between different research articles; (3) the article did not provide numeric values of the results; or (4) the original full text of the article could not be found. With the inclusion and exclusion criteria, a total of 402 research articles were selected for the 14 different feed additives frequently used in grow–finish pig diets.

### 2.3. Data Extraction and Analysis

Article information, treatment design, response variables, and statistical results were extracted from the selected articles. Article information included authors, published journal, article type, title, published year, and the location of the study. Treatment design included the feed additive used, the form of the additive, feed additive inclusion level, duration of the study, the first 2 major ingredients in the diet (e.g., corn–soybean meal, barley–wheat), pig breed, sex, housing type (individual or group pen), and initial BW. Response variables included final BW, ADG, ADFI, G:F, carcass weight, BF, percentage lean, and LMA/LD. Statistical results included the standard error of the mean (SEM) of the response of interest and the *p*-value of the response of interest compared to the control. Because the reported values of BF vary on the sampling locations, if the location was reported, the priority sequence of extracted value was average BF, 10th rib BF, last rib BF, loin BF, and other locations. If the location was not reported, the value was extracted as listed in the article regardless of the location. Because of the similarity between LMA and LD, both values were extracted for the same category. If both values were reported, LMA was prioritized over LD. The extracted data of each treatment group was entered into the database as a row of data.

The relative difference in the response between the treatment group and the control was calculated as the percentage of difference and defined as a comparison in this literature review. The determination of significance, *p*-value, and response value were based on the study design and statistical analysis. The significance of each comparison was categorized as significant if the reported *p*-value was below or at 0.05 (*p* ≤ 0.05). The comparisons were categorized as tendency if the reported *p*-value was between 0.05 and 0.10 (0.05 < *p* ≤ 0.10) and as non-significant if the reported *p*-value was above 0.10 (*p* > 0.10). For studies that only reported whether the *p*-value was below or above 0.05 and it cannot be determined whether there was a tendency (0.05 < *p* ≤ 0.10), the comparisons were categorized as significant (*p* ≤ 0.05) or non-significant (*p* > 0.10). For studies that utilized polynomial contrasts, if the polynomial *p*-value was significant or indicated a tendency, the same *p*-value was assigned to all comparisons in the polynomial contrast despite the numeric difference in the response, to reflect the general effect of adding the additive on finding a significant difference. If the polynomial *p*-value was not significant, the determination of the *p*-value was based on the *p*-value of the pairwise comparison if available. If the pairwise comparison was unavailable, the non-significant polynomial *p*-value was used for all comparisons. For studies with factorial treatment structure, the combined means and main effect *p*-values were extracted if there was no significant interactive effect, regardless of the other factors. If there was a significant or a tendency of interactive effect of either variable, all possible comparisons were extracted separately for all variables of interest. However, if the other factor in the basal diets was the addition of ractopamine, the data was not extracted. For each response of the feed additive, the number of the extracted comparisons was counted, and the percentage difference was used to summarize the average positive, neutral, and negative effects of the feed additives at each significant level as a descriptive statistical analysis.

**Figure 1:**
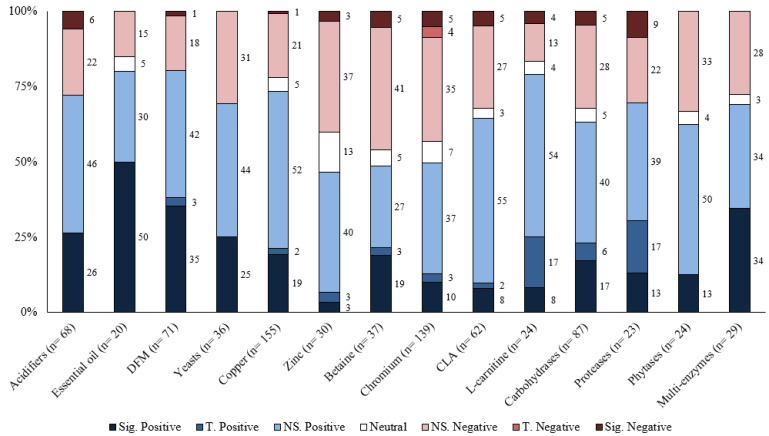
The distribution of results of various feed additives on ADG by significance level and direction. The comparison was significant (Sig.) if *p* ≤ 0.05, had a tendency (T.) if 0.05 < *p* ≤ 0.10, and was non-significant (NS.) if *p* > 0.10.

**Figure 2:**
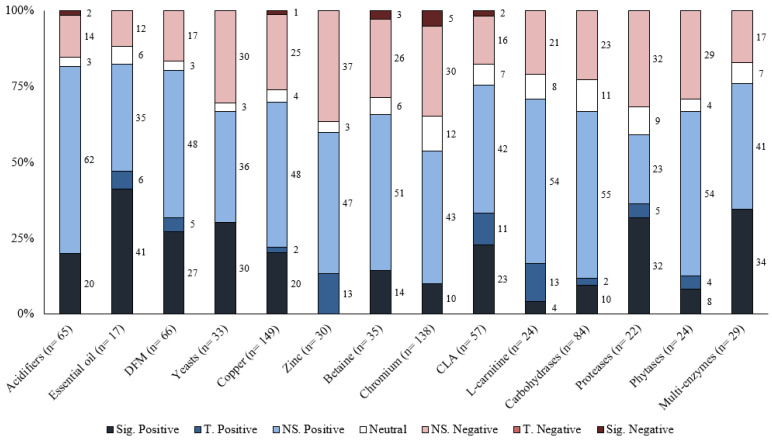
The distribution of results for various feed additives on G:F by significance level and direction. The comparison was significant (Sig.) if *p* ≤ 0.05, had a tendency (T.) if 0.05 < *p* ≤ 0.10, and was non-significant (NS.) if *p* > 0.10.

**Figure 3:**
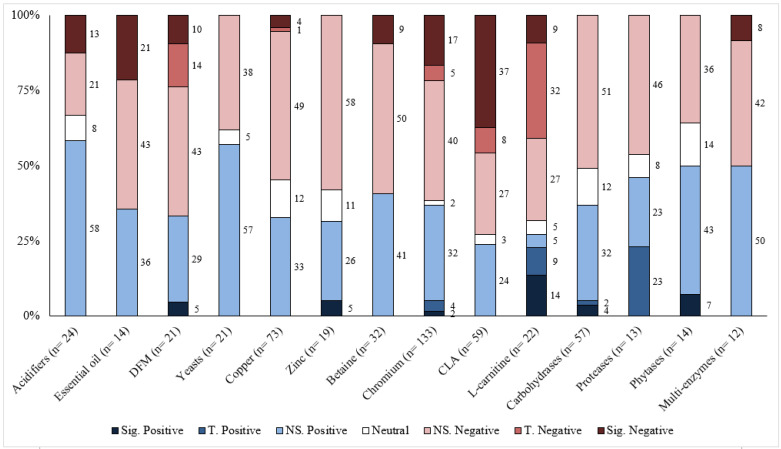
The distribution of result for various feed additives on BF by significance level and direction. The comparison was significant (Sig.) if *p* ≤ 0.05, had a tendency (T.) if 0.05 < *p* ≤ 0.10, and was non-significant (NS.) if *p* > 0.10.

**Figure 4:**
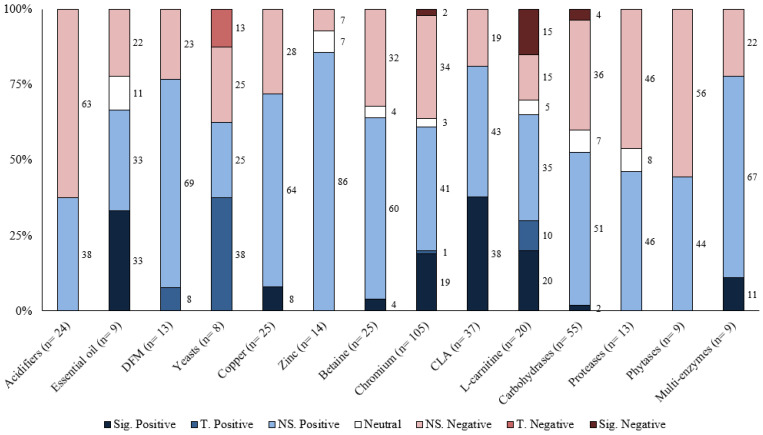
The distribution of results for various feed additives on percentage lean by significance level and direction. The comparison was significant (Sig.) if *p* ≤ 0.05, had a tendency (T.) if 0.05 < *p* ≤ 0.10, and was non-significant (NS.) if *p* > 0.10.

**Figure 5:**
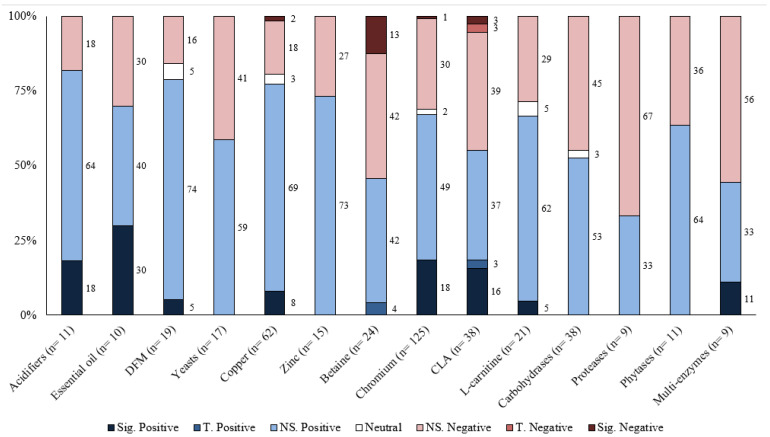
The distribution of results for various feed additives on longissimus muscle area/loin depth by significance level and direction. The comparison was significant (Sig.) if *p* ≤ 0.05, had a tendency (T.) if 0.05 < *p* ≤ 0.10, and was non-significant (NS.) if *p* > 0.10.

Responses to each feed additive are summarized for the different response criteria (growth performance and carcass characteristics) based on the number of comparisons, magnitude of improvement, and statistical significance levels in Table 1, Table 2, Table 3, Table 4 and Table 5. Moreover, the distribution of results for each feed additive was summarized by the significance level and direction of improvement in Figure 1, Figure 2, Figure 3, Figure 4 and Figure 5. Because of the large number of studies included in the review, the detailed summaries of each additive category, the extracted results of every comparison, and citations were reported in the supplemental paper (Tables S1–S22).

## 3. Feed Additives—Health

This section discusses the feed additives that can potentially improve growth performance and carcass characteristics by enhancing the health status of grow–finish pigs. The feed additives discussed are acidifiers, essential oils, DFM, yeasts, Cu, and Zn.

### 3.1. Acidifiers—Mechanism of Action

Acidifiers have been used in animal diets for their beneficial effects on antimicrobial activity and nutrient digestibility coefficients. The most used acidifiers are organic acids in the form of short-chain fatty acids (fumaric, citric, malic, formic, lactic, acetic, butyric, and propionic acid), medium-chain fatty acids (sorbic, capric, and caprylic acid), and benzoic acid. Acidifiers lower the pH of the digestive tract, which provides an acidic environment (pH < 4.5) that inhibits the growth of acid-sensitive bacteria [2]. The low pH also assists the digestibility of protein and minerals by stimulating the secretion and activity of enzymes in the small intestine [17,18]. Moreover, in an acidic environment, non-dissociated organic acids can freely penetrate the bacterial cell wall and reduce the pH of cytoplasm [2]. The increased H^+^ requires bacteria to spend energy on removing these H^+^ and, therefore, retards the growth of acid-sensitive pathogens [2]. With these mechanisms, acidifiers can potentially improve growth performance and carcass characteristics by enhancing the gut health and digestibility of the pig [2,17,18].

### 3.2. Acidifiers—Results

There were 68 comparisons for ADG between pigs fed a control diet or diets with added acidifiers with an average of a 1.7% increase (range between −14.9 and 11.4%) in pigs fed acidifiers, and for G:F, there were 65 comparisons between pigs fed a control diet or diets with added acidifiers with an average of a 3.1% increase (range between −9.7 and 11.3%) in pigs fed acidifiers. For carcass data, there were 24 comparisons evaluating BF change between pigs fed a control diet or diets with added acidifiers with an average of a 0.6% decrease (range between −15.3 and 14.4%) in pigs fed acidifiers. For percentage lean, there were 24 comparisons between pigs fed a control diet or diets with added acidifiers with an average of a 0.5% decrease (range between −3.6 and 4.2%) in pigs fed acidifiers. There were 11 comparisons for LMA/LD between pigs fed a control diet or diets with added acidifiers with an average of a 1.6% improvement (range between −7.2 and 8.1%) in pigs fed acidifiers. These results could be expected because the mechanisms do not directly affect protein and lipid metabolism. In summary, feeding acidifiers has the potential to improve growth performance but only minor effects on carcass characteristics.

### 3.3. Essential Oils (EO)- Mechanism of Action

Essential oils (ethereal oils) are classified as phytogenic feed additives. Essential oils are a mixture of volatile and non-volatile compounds extracted from plants (approximately 1% of the wet weight of plants), such as oregano, thyme, rosemary, and garlic [19]. The primary active ingredients in EO are phenols (thymol, carvacrol, eugenol, ρ-cymene). These phenolic components have been widely used for antibacterial, antiviral, antifungal, insecticidal, and antiparasitic activities in humans and animals [5]. For the antibacterial effects, the lipophilic structures of EO can penetrate and disrupt the cell wall and cell membrane of the pathogens, which causes alterations in the cell functions [20], which is similar to the antimicrobial mechanism of the organic acids. The phenolic OH group can also act as an antioxidant by donating hydrogen to free radicals [19]. Moreover, EO may potentially enhance the immune system by interacting with the microbiota of the pigs and altering the lymphocyte population and distribution in the gut [19]. These beneficial mechanisms suggest that EO may potentially improve grow–finish pig growth performance and carcass characteristics.

### 3.4. Essential Oils—Results

For ADG, there were 20 comparisons between pigs fed a control diet or diets with added EO with an average of a 5.8% improvement (range between −2.9 and 18.8%) in pigs fed EO. There were 17 comparisons for G:F between pigs fed a control diet or diets with added EO with an average of a 5.8% improvement (range between −2.6 and 19.9%) in pigs fed EO. Fourteen comparisons evaluated BF between pigs fed a control diet or diets with added EO with an average of a 2.7% decrease (range between −14.2 and 6.3%) in pigs fed EO. For percentage lean, there were 9 comparisons with an average of a 0.9% improvement (range between −2.5 and 2.8%) in pigs fed EO. For LMA/LD, there was an average of a 1.9% improvement (range between −6.3 and 12.3%) in pigs fed EO.

Overall, the results suggest that EO positively affected ADG and G:F. Adding EO alone or in combination with acids has the potential to improve growth performance. However, there was only a small amount of research on EO’s effect on growth performance, and only three studies were conducted in the US; therefore, using EO may not be beneficial in US-based conditions. More experiments are needed to determine the effect of including EO in the diets of grow–finish pigs.

### 3.5. Direct-Fed Microbials (DFM) -Mechanism of Action

Direct-fed microbial (DFM) or probiotic products are defined as feed additives that contain live (viable) microorganisms (bacteria and/or yeast) that are beneficial to the host. The most used DFM strains added in grow–finish pig diets are yeast (*Saccharomyces cerevisiae*), and *Bacillus* and *Lactobacillus* species either as a single strain or blend based on the articles we collected (the effect of the single addition of yeast in diets was discussed in the yeast section). Adding DFM aims to achieve a healthy and balanced intestinal microbial composition [4]. These beneficial microorganisms may improve the digestibility of nutrients and reduce the adverse effects of pathogens in the gastrointestinal tract by competitive exclusion, modulation of the immune response, and/or the production of bacteriocins [21]. The inclusion of DFM has been used as an alternative to antibiotics and has shown beneficial effects in research when fed mainly in weaned pig diets.

### 3.6. DFM—Results

There were 71 comparisons for ADG between pigs fed a control diet or diets with added DFM with an average of a 3.3% improvement (range between −6.2 and 20.3%) in pigs fed DFM. For G:F, there were 66 comparisons between pigs with an average of a 3.3% improvement (range between −7.2 and 13.1%) in pigs fed DFM. There was an average 1.5% decrease (range between −18.1 and 20.3%) in BF for pigs fed a DFM vs. a control diet across 21 comparisons. For percentage lean, there were 13 comparisons between pigs fed a control diet or diets with added DFM with an average of a 1.0% improvement (range between −2.0 and 3.6%) in pigs fed DFM. There were 19 comparisons evaluating added DFM for LMA/LD, with an average of a 1.5% improvement (range between −5.8 and 10.9%) in pigs fed DFM.

In summary, DFM can potentially improve the growth performance of grow–finish pigs. However, the small effects and lack of statistical differences of DFM on carcass characteristics may suggest that the mechanisms of DFM do not directly affect pigs’ protein and lipid metabolism. It is worth mentioning that there were relatively few US-based studies for DFM; therefore, the effects of DFM in US-based conditions may not be the same as what has been observed to date.

### 3.7. Yeasts—Mechanism of Action

Yeast is a single-cell fungus used in the food industry, ethanol production, and animal feed for its nutritional and health benefits. The most used yeast strain in animal feed is *Saccharomyces cerevisiae*, while *Phaffia rhodozyma* (red yeast) is rarely used. Yeast products are added as live yeast (as a DFM additive), yeast cell wall extracts, or a combination of both. Yeast converts substrates (carbon and nitrogen sources) into carbon dioxide, ethanol, and yeast cell contents through fermentation [6]. The fermented yeast cell culture contains vitamin B, β-glucan, α-mannans polysaccharides, and microbial protein, which can serve as a protein source for animals. The yeast cell wall extracts mainly consists of β-glucan and α-mannan polysaccharides, which have shown prebiotic effects on improving nursery pigs’ immune system and gastrointestinal health [6]. Mannan oligosaccharides (MOS) are the side chains of mannan polysaccharides and have been widely studied as an antimicrobial feed additive for their positive effects on microbiota and intestinal morphology in nursery pigs [22]. In addition, MOS reduces the colonization of pathogens by binding to the pathogens and improves gut morphology by increasing the villus height:crypt depth ratio [22].

### 3.8. Yeasts—Results

There were 36 comparisons for ADG between pigs fed diets with added yeasts with an average of a 1.6% improvement (range between −13.7 and 10.3%) in pigs fed yeasts. For G:F, there were 33 comparisons between pigs fed a control diet or diets with added yeasts with an average of a 2.7% improvement (range between −11.7 and 17.7%) in pigs fed diets containing yeasts. There were 21 comparisons evaluating pigs fed diets with added yeasts on BF with an average of a 3.1% decrease (range between −30.7 and 11%) in pigs fed yeasts. For percentage lean, there were 8 comparisons between pigs fed a control diet or diets with added yeasts with an average of a 1.0% improvement (range between −1.7 and 6.6%) in pigs fed yeasts. Lastly, for LMA/LD, there were 17 comparisons with pigs fed added yeasts having an average of a 1.4% improvement (range between −4.3 and 16.6%).

In summary, yeasts can be a potential feed additive with a relatively large magnitude of improving the growth performance of grow–finish pigs, especially for growth performance.

### 3.9. Copper (Cu)—Mechanism of Action

Copper is an essential trace mineral for several metalloenzymes that play roles in oxidation–reduction reactions, transport of oxygen and electrons, and protection against oxidative stress [3]. Feeding pharmacological levels of Cu has shown growth-promoting effects in weaned and growing pigs by reducing diarrhea frequency and increasing feed efficiency [23,24]. These improvements may be because of Cu’s effects on the enzymes (lipase, phospholipase A, lipoprotein lipase) involved in lipid digestion and metabolism [3]. Copper also showed bacteriostatic and bactericidal properties that improve weaned pigs’ microbiota, gastrointestinal structure, and immune status [25,26]. However, because Cu accumulates in the liver and other organs when fed above requirement estimates, toxicity should be a concern when provided above 250 mg/kg in pig diets. Feeding excess levels of Cu resulted in hemolysis and organ damage in pigs [3].

### 3.10. Copper—Results

There were 155 comparisons of ADG between pigs fed a control diet or diets with added Cu with an average of a 2.5% improvement (range between −12.2 and 15.2%) in pigs fed pharmacological levels of added Cu. For G:F, there were 149 comparisons between pigs fed a control diet or diets with added Cu with an average of a 1.8% improvement (range between −8.0 and 17.6%) in pigs fed Cu. Seventy-three comparisons evaluated BF between pigs fed diets with added Cu with an average of a 1.4% decrease (range between −17.0 and 11.5%) in BF of pigs fed Cu. For percentage lean, there were 25 comparisons with pigs fed added Cu having an average improvement of 1.6% (range between −2.7 and 34.7%). For LMA/LD, there were 62 comparisons between pigs fed diets with added Cu with an average of a 2.3% improvement (range between −7.5 and 14.5%).

Most studies used Cu additions of 125 to 250 mg/kg (137 comparisons), and increasing Cu addition did not generally further improve pig performance. The growth-promoting effects of Cu can potentially improve growth performance (2.5 and 1.8% improvement for ADG and G:F); however, with carcass characteristics, the effects were relatively small, with most comparisons finding no evidence of difference.

### 3.11. Zinc (Zn)—Mechanism of Action

Zinc is an essential trace mineral in several important metalloenzymes for the growth and development of animals. High levels (1500 to 4000 mg/kg) of dietary zinc oxide (ZnO) have been widely used as a growth-promotive feed additive in weaned pig diets to improve growth performance and gastrointestinal health [7]. However, the mechanisms of the growth-promotive effect of ZnO are still not fully understood. Zinc oxide may regulate the secretion of ions in the intestine, reduce the inflammatory reaction, stabilize the microbiota, prevent the attachment of pathogens, and improve the gastrointestinal structure [7]. Moreover, for grow–finish pigs, whether high Zn inclusion (above 100 mg/kg) can provide a growth-promotive effect is also unclear.

### 3.12. Zinc—Results

For ADG and G:F, there were 30 comparisons between pigs fed a control diet or diets with added Zn with increases of 0.6% (range between −14.4 and 18.7%) and 1.2% (range between −7.6 and 14.4%), respectively. There were 19 comparisons for BF and pigs fed diets with added Zn had an average of a 0.6% decrease (range between −7.6 and 13.1%). For percentage lean, there were 14 comparisons that observed an average 0.9% improvement (range between −0.4 and 3.9%) in pigs fed Zn. All the comparisons (15) found a 0.2% improvement (range between −2.9 and 2.7%) in LMA/LD.

Overall, the results suggest that Zn had positive but relatively small effects on ADG, G:F, and carcass characteristics. Moreover, there were insufficient data to support whether different types of basal diets and inclusion levels affected the response to added Zn.

## 4. Feed Additives—Energy and Lipid Metabolism

This section discusses the feed additives that have the potential to improve growth performance and carcass characteristics by affecting the energy and lipid metabolism of grow–finish pigs. The feed additives discussed are betaine, Cr, CLA, and L-carnitine.

### 4.1. Betaine—Mechanism of Action

Betaine is a trimethyl derivative of glycine that can be widely found in plants and animals. It serves as a methyl group donor along with choline and methionine, and plays a role in synthesizing carnitine, creatine, and methylated AAs. It can also improve the metabolism of methionine by donating a carbon molecule for the remethylation of methionine from homocysteine [8]. In pigs, betaine supplementation increased serum growth hormone (GH) and insulin growth factor 1 (IGF-1), which may improve protein synthesis and growth performance [27,28]. Betaine supplementation may also improve energy utilization, resulting in improved growth performance [29]. For meat quality, betaine is likely to regulate genes responsible for the uptake and oxidation of fatty acids in the muscle, and therefore reduce the body fat percentage of the pigs and change the free fatty acid concentration in muscles. Betaine can also delay the anaerobic glycolysis after slaughter, affecting muscle pH, pork color, and water-holding capacity [8].

### 4.2. Betaine—Results

There was an average of a 1.3% improvement (range between −8.3 and 27.5%) in ADG across 37 comparisons for pigs fed betaine vs. those fed a control diet. For G:F, there were 35 comparisons and those fed added betaine had on average a 2.7% improvement (range between −6.3 and 23.2%). For BF, there were 32 comparisons between pigs fed a control diet or diets with added betaine with an average of 1.7% in favor of pigs fed betaine (range between −18.6 and 7.4%). For percentage lean and LMA/LD, there were 25 and 24 comparisons, respectively, between pigs fed a control diet or diets with added betaine with an average of a 2.0% (range between −4.8 and 12.4%) and 0.20% (range between −7.0 and 8.9%) improvement in pigs fed betaine.

There were insufficient data to support whether different types of basal diets affected the response to betaine for ADG and G:F. However, betaine may have a more beneficial effect on ADG and G:F in limit-fed pigs [30,31]. In summary, the results suggest that betaine had relatively small positive effects on ADG but may benefit G:F more. Adding dietary betaine to finishing pig diets only significantly affected carcass characteristics in a few experiments.

### 4.3. Chromium (Cr)—Mechanism of Action

Chromium affects several enzymes (adenosine monophosphate-activated protein kinase, tyrosine kinase, etc.) and hormones (IGF-1, triiodothyronine, etc.) that regulate energy metabolism, protein accretion, and fat deposition [9]. The primary mechanism of action for Cr is potentiating the action of insulin by facilitating the binding of insulin to receptors on cell membranes which increases the translocation of glucose transporter type 4 to plasma membrane and thus improves glucose utilization of these cells [9,32]. For muscle cells, Cr increases glucose and AAs uptake and improves energy metabolism which increases lean accretion and reduces fat deposition [9]. Therefore, Cr has the potential to improve feed efficiency and carcass characteristics of grow–finish pigs.

### 4.4. Chromium—Results

There were 138 comparisons for ADG and G:F between pigs fed a control diet or diets with added Cr with an average of a 1.1% (range between −21.1 and 21.2%) and 1.0% (range between −10.3 and 10.3%) improvement, respectively. In addition, many experiments (133) also evaluated BF change when pigs were fed added Cr with an average of a 3.9% decrease (range between −31.4 and 15%) in BF depth. For percentage lean, there were 105 comparisons with pigs fed added Cr having an average of a 1.6% improvement (range between −7.4 and 14.1%). For LMA/LD, there were 125 comparisons with pigs fed added Cr having an average of a 3.1% (range between −11.6 and 22.6%) improvement.

According to our database, Cr slightly improved ADG and G:F. This is in agreement with a meta-analysis that analyzed data from 31 studies and found that grow–finish pigs fed 200 to 500 µg/kg Cr had improved (*p* ≤ 0.05) ADG and G:F compared with the control pigs [33]. Chromium’s effect on improving BF, percentage lean, and LMA/LD was relatively large and more consistent than many of the other feed additives reviewed.

### 4.5. Conjugated Linoleic Acid (CLA)—Mechanism of Action

Conjugated linoleic acid is a collective term for fatty acids with 18 carbon-atom structures that are geometric isomers of linoleic acid [34]. These fatty acids contain 2 double bonds on either positions 9 and 11 or 10 and 12 in *cis* or *trans* configuration [10]. Conjugated linoleic acids play a significant role in lipid metabolism by inhibiting glucose entry into adipocytes and increasing the activities of nuclear transcription factors and enzymes that affect fatty acid catabolism; therefore, these effects reduce lipogenesis and potentiate lipolysis through β-oxidation [10]. Thus, CLA can potentially improve growth performance by regulating energy metabolism and improving carcass composition by reducing adipose tissue and increasing lean tissue.

### 4.6. CLA—Results

A 2.1% (range between −14.5 and 14.1%) and 3.5% (range between −7.4 and 16.7%) improvement in ADG and G:F, respectively, were observed in pigs fed added CLA across 57 comparisons. Fifty-nine comparisons with pigs fed added CLA observed an average of a 7.0% decrease (range between −27.5 and 18%) in BF. For percentage lean, there were 37 comparisons with an average of a 2.6% (range between −1.8 and 9.1%) improvement in pigs fed CLA. However, for LMA/LD, there was only an average of a 0.9% increase (range between −7.5 and 11%) with added CLA.

In summary, CLA may improve growth performance, with the greatest chance for improvement being elicited for G:F. In addition, CLA has the potential to reduce BF and increase percentage lean more consistently compared with other feed additives considered in this review.

### 4.7. L-carnitine—Mechanism of Action

L-carnitine is an essential molecule that transports long-chain fatty acids into the mitochondrial matrix, where the fatty acids are oxidized for energy production through β-oxidation [11]. L-carnitine can also promote energy production by regulating important key enzymes for glycolysis and the tricarboxylic acid cycle [11]. However, the concentration of L-carnitine is low in plants (e.g., corn and soybean) typically used in animal feeds [11]. Even though pigs can produce endogenous L-carnitine, its production is affected by the pig’s micronutrient status, and in some situations, endogenous production or renal absorption may not satisfy the requirements [11]. Due to these reasons, the addition of L-carnitine in plant-based swine diets has been investigated in numerous studies for its potential to improve performance and carcass characteristics.

### 4.8. L-carnitine—Results

For ADG and G:F, there were 24 comparisons evaluating pigs fed a control diet or diets with added L-carnitine with an average of a 2.1% (range between −4.8 and 9.4%) and 2.5% (range between −3.6 and 7.7%) increase, respectively, observed in pigs fed L-carnitine. For BF, there were 22 comparisons and an average of a 3.4% decrease (range between −18.2 and 4.8%) in pigs fed L-carnitine. Percentage lean significantly increased (*p* ≤ 0.05) by an average of 3.8% (range between −2.6 and 7.6%) for pigs fed added L-carnitine. For LMA/LD, there was an average of a 2.4% improvement (range between −6.4 and 16.2%) in pigs fed L-carnitine (21 comparisons).

Overall, the results suggest that L-carnitine has the potential to improve ADG and G:F (79 and 71% of all the comparisons, respectively) with relatively large improvements. In addition, the results also suggest that L-carnitine is a potential feed additive that had relatively large effects on BF, percentage lean, and LMA/LD compared to other feed additives evaluated in this review.

## 5. Feed Additives—Enzymes

This section discusses dietary enzymes used as feed additives in classes of carbohydrases, proteases, phytases, and combination of different types of enzymes (multi-enzymes).

### 5.1. Mechanism of Action—Enzymes

Because endogenous digestive enzymes of pigs cannot fully digest various feed substances (e.g., fiber and phytate), exogenous enzymes are included in diets to improve the digestion of these feed ingredients [12]. Moreover, indigestible fibrous substances can entrap nutrients or negatively affect digestion. Carbohydrases (xylanase, glucanase, mannanase, etc.) can break down some indigestible non-starch polysaccharides (cell wall and fiber) of the plant-based ingredients and release previously unavailable nutrients for the animals [13]. Moreover, the products of this degradation process may be beneficial for gut health [13]. In addition, proteases assist the breakdown of dietary protein to improve utilization and reduce excess protein in the hindgut and manure.

The main reason for adding phytases is to break down dietary phytate and release phytate-bound [35]. Furthermore, phytases may also improve the digestibility of other nutrients (i.e., energy, AAs, minerals, etc.) by reducing the negative effect of dietary phytate on these nutrients [36]. Blending different enzymes into multi-enzyme complexes is common with the intent of combining the benefits because of different enzymatic mechanisms.

### 5.2. Carbohydrases—Results

For ADG, there were 87 comparisons with pigs fed a control diet or diets with added carbohydrases with an average of a 1.3% improvement (range between −9.3 and 14.8%). For G:F, there were 84 comparisons with an average of a 1.7% improvement (range between −12.3 and 16.5%) in pigs fed carbohydrases. There were over 50 comparisons evaluating BF and percentage lean between pigs fed a control diet or diets with added carbohydrases with an average of a 0.4% decrease (range between −17 and 16.1%) or a 0.40% increase (range between −4.8 and 8.1%) in BF and percentage lean, respectively. For LMA/LD, there were 38 comparisons between pigs fed a control diet or diets with added carbohydrases with an average of a 1.1% improvement (range between −3.5 and 12.7%) in pigs fed carbohydrases. Overall, results suggest that carbohydrases had positive effects on ADG and G:F (63 and 67% of all the comparisons, respectively), but the magnitude was small, and most comparisons had no statistical differences. The mixed and relatively small and non-significant responses are expected because the mechanisms of exogenous enzymes assist the digestion of feed substrates but do not affect protein and lipid metabolism. The high percentage of non-significant comparisons also suggested that the carcass results were highly variable.

### 5.3. Proteases—Results

There were 23 comparisons for ADG between pigs fed a control diet or diets with added proteases with an average of a 0.6% improvement (range between −9.8 and 6.0%) in pigs fed proteases. For G:F, there were 22 comparisons with an average of a 1.8% improvement (range between −4.3 and 15.1%) in pigs fed proteases. Comparisons between pigs fed a control diet or diets with added proteases showed an average of a 0.1% decrease (range between −8.3 and 10.8%) in BF of pigs fed proteases. There was no difference (range between −2.1 and 2.4%) in percentage lean across 13 comparisons between pigs fed a control diet or diets with added proteases. For LMA/LD, there were 9 comparisons between pigs fed a control diet or diets with added proteases with an average of a 2.0% decrease (range between −6.5 and 5.4%) in pigs fed proteases.

Overall, there were relatively small and non-significant results observed in growth performance and carcass characteristics when proteases were included in grow–finish pig diets.

### 5.4. Phytases—Results

An average of a 1.1% improvement (range between −4.6 and 10.6%) in ADG of pigs fed phytases was observed across 24 comparisons between pigs fed a control diet or diets with added phytases. Feed efficiency was improved by 1.1% (range between −6.6 and 6.9%) across 24 comparisons between pigs fed a control diet or diets with added phytase. There were 14 comparisons for BF between pigs fed a control diet or diets with added phytases with an average of a 0.2% decrease (range between −6.7 and 8.3%) in pigs fed phytases. No differences (range between −0.8 and 1.3%) were observed in percentage lean with 9 comparisons between pigs fed a control diet or diets with added phytases. For LMA/LD, there were 11 comparisons between pigs fed a control diet or diets with added phytases with an average of a 1.4% decrease (range between −11.6 and 3.5%) in pigs fed phytases.

In summary, including phytase in diets with adequate P may not affect finishing pig ADG and G:F. Furthermore, there were relatively small and non-significant results observed in carcass characteristics when phytases were included in grow–finish pig diets.

### 5.5. Multi-Enzymes—Results

For ADG, there were 29 comparisons between pigs fed a control diet or diets with added multi-enzymes with an average of a 3.1% improvement (range between −6.5 and 24.9%) by the addition of multi-enzymes. A 3.3% improvement (range between −6.6 and 30.8%) in G:F of pigs fed multi-enzymes was observed across 29 comparisons. There were 12 comparisons for BF between pigs fed a control diet or diets with added multi-enzymes with an average of a 2.8% increase (range between −12.3 and 29.4%) in pigs fed multi-enzymes. There were 9 comparisons in percentage lean and LMA/LD between pigs fed a control diet or diets with added multi-enzymes with an average of a 0.7% (range between −1.3 and 4.4%) and 0.30% (range between −3.2 and 11.3%) improvement in pigs fed multi-enzymes. Overall, results suggest that multi-enzymes positively affect ADG and G:F. There were relatively small and non-significant results observed in carcass characteristics when multi-enzymes are included in grow–finish pig diets. Moreover, the combination of multiple enzymes provided greater improvement than adding any single type of enzyme (carbohydrase, protease, and phytase) alone according to our summaries, which suggests that different types of enzymes may have a synergetic effect; however, some factorial studies that used multi enzyme types found combining enzyme types do not improve performance [37,38].

Furthermore, most comparisons showed little or negative effects in US-based research; therefore, US-based diets with multi-enzymes should be evaluated further.

## 6. Discussion

Overall, the greatest proportion of the comparisons for each feed additive was positive; however, most of them were also not statistically significant (Table 1, Table 2, Table 3, Table 4 and Table 5). For most feed additives, there were enough comparisons to show the general effects on ADG and G:F. For carcass characteristics, the overall effects were also positive; however, there were fewer comparisons, and effects were mostly small and inconsistent (Figure 1, Figure 2, Figure 3, Figure 4 and Figure 5). Moreover, the sampling process for carcass characteristics often only selects a smaller portion of the animals that were used for the growth performance data, which accentuated the between-animal variations for the relatively small treatment effects. This potentially resulted in a higher proportion of the comparisons having no evidence of difference for carcass characteristics.

For utilizing these results in US-based production, the results suggest that Cr, carbohydrases, proteases, phytases, and Zn had minor effects and did not appear to be potential feed additives based on growth performance. Essential oils consistently improved ADG and G:F with a relatively larger magnitude (>3%), but the number of comparisons was low, and most studies were not US-based; therefore, publication bias and locational effect should be concerned. On the other hand, acidifiers, betaine, CLA, L-carnitine, and yeasts had relatively substantial positive effects (2.5 to 3.5 %) on G:F. Moreover, despite limited data, benzoic acid and other acidifiers may be potential additives for improving ADG and G:F, but further research is needed. The effects of Cu were most studied, with a 2.5% improvement in ADG, but the average effect on G:F was minor (1.8%). Moreover, DFM and multi-enzymes had relatively large and consistent improvements (approximately 3%) in ADG and G:F with a sizeable number of comparisons; however, there were relatively few US-based studies for DFM. Therefore, acidifiers, betaine, CLA, DFM, multi-enzymes, L-carnitine, and yeasts may have the greatest opportunity to improve finishing pig G:F. However, their concentration and feeding strategies need further research. Lastly, betaine, Cr, CLA, and L-carnitine may potentially improve carcass characteristics because of their effects on lipid and energy metabolism.

Additionally, even though we collected all the known research studies, publication bias still needs to be kept in mind when interpreting the results of this literature review. Furthermore, most research was conducted in well-controlled research facilities that only utilized a small number of pigs per experiment; therefore, it may have limitations in representing the whole pig population. Moreover, compared with pigs in commercial settings, these research pigs were observed closely, experienced relatively little environmental stress (e.g., space allowance and temperature), and often had better health status and high feed intake (nutrient intake) relative to the pigs’ requirements. Therefore, adding these feed additives may be more advantageous in pigs’ diets in commercial settings where pigs are not under optimal conditions. More commercial research should be conducted to understand the effects of additives used under these conditions. Additionally, to utilize these results, the location of these studies should also be considered because some additives (e.g., DFM and EOs) had large positive responses in some countries, but, in contrast, the US-based results showed neutral or negative responses. These may be due to the differences in pig genetics, farm environments, formulated nutrient levels, and diet compositions. Lastly, even though economics was not discussed in this review, the decision to include feed additives in pig diets should consider the return of investment based on the price and the magnitude of benefit of the feed additive. For example, a relatively small magnitude of G:F improvement may still be economical when provided by a relatively low-priced feed additive. On the other hand, a feed additive with a relatively large magnitude of improvement in G:F may not be economical if it has a relatively high price.

## 7. Conclusions

In conclusion, this literature review collected available research on finishing pig feed additives to provide a descriptive analysis of the effects on growth and carcass performance. In addition, this database has the potential to be further analyzed with advanced statistical methods, such as meta-analysis, to figure out the reasons behind the variable results when additives were added, and possibly lead to a better understanding of the effect of feed additives to improve the efficiency of swine production.

## Figures and Tables

**Table 1:** Summary of the effects of feed additives on grow–finish pig ADG ^1,3^.

			Positive			Neutral	Negative		
Item	Comparisons, n	Difference, % ^2^	Sig.	Tendency	NS.		NS.	Tendency	Sig.
Acidifiers	68	1.7	18 (5.8)	0	31 (3.4)	0	15 (−3.4)	0	4 (−10.8)
Essential oils	20	5.8	10 (9.9)	0	6 (3.8)	1	3 (−1.7)	0	0
DFM	71	3.3	25 (6.3)	2 (3.9)	30 (3.6)	0	13 (−2.3)	0	1 (−5.8)
Yeasts	36	1.6	9 (5.6)	0	16 (3.2)	0	11 (−4.1)	0	0
Copper	155	2.5	30 (6.2)	3 (4.1)	81 (3.8)	7	33 (−3.4)	0	1 (−0.1)
Zinc	30	0.6	1 (18.7)	1 (1.1)	12 (4.0)	4	11 (−3.2)	0	1 (−14.4)
Betaine	37	1.3	7 (10.6)	1 (4.3)	10 (2.4)	2	15 (−3.3)	0	2 (−2.8)
Chromium	139	1.1	14 (8.9)	4 (4.6)	51 (3.6)	10	48 (−2.2)	5 (−4.1)	7 (−7.2)
CLA	62	1.2	5 (7.2)	1 (3.6)	34 (3.7)	2	17 (−4.1)	0	3 (−7.8)
L-carnitine	24	2.1	2 (3.3)	4 (3.1)	13 (3.4)	1	3 (−2.6)	0	1 (−4.8)
Carbohydrases	87	1.3	15 (5.3)	5 (4.0)	35 (2.9)	4	24 (−3.3)	0	4 (−2.7)
Proteases	23	0.6	3 (5.2)	4 (3.2)	9 (2.1)	0	5 (−3.7)	0	2 (−7.6)
Phytases	24	1.1	3 (6.8)	0	12 (2.6)	1	8 (−3.0)	0	0
Multi-enzymes	29	3.1	10 (7.9)	0	10 (2.9)	1	8 (−2.3)	0	0

^1^ Significant (Sig.; *p* ≤ 0.05), tendency (0.05 < *p* ≤ 0.10), and non-significant (NS.; *p* > 0.10). ^2^ Average of the % of difference of all the comparisons. ^3^ Number outside of the parentheses represents the number of comparisons. Number inside the parentheses represents the average of the percentage of difference of these comparisons.

**Table 2:** Summary of the effects of feed additives on grow–finish pig G:F ^1,3^.

			Positive			Neutral	Negative		
Item	Comparisons, n	Difference, % ^2^	Sig.	Tendency	NS.		NS.	Tendency	Sig.
Acidifiers	65	3.1	13 (6.4)	0	40 (3.8)	2	9 (−3.1)	0	1 (−9.7)
Essential oils	17	5.8	7 (10.9)	1 (4.5)	6 (3.5)	1	2 (−1.5)	0	0
DFM	66	3.3	18 (6.1)	3 (3.0)	32 (3.9)	2	11 (−2.2)	0	0
Yeasts	33	2.7	10 (7.8)	0	12 (3.9)	1	10 (−3.6)	0	0
Copper	149	1.8	30 (5.1)	3 (1.0)	71 (3.1)	6	37 (−2.7)	0	2 (−3.7)
Zinc	30	1.2	0	4 (1.2)	14 (4.2)	1	11 (−2.6)	0	0
Betaine	35	2.7	5 (13.2)	0	18 (2.7)	2	9 (−2.3)	0	1 (−0.4)
Chromium	138	1.0	14 (5.2)	0	60 (3.1)	16	41 (−2.1)	0	7 (−4.3)
CLA	57	3.5	13 (4.5)	6 (8.8)	24 (4.6)	4	9 (−2.3)	0	1 (−2.8)
L-carnitine	24	2.5	1 (2.9)	3 (3.7)	13 (4.4)	2	5 (−2.0)	0	0
Carbohydrases	84	1.7	8 (8.5)	2 (5.9)	46 (2.9)	9	19 (−3.8)	0	0
Proteases	22	1.8	7 (4.9)	1 (7.6)	5 (2.2)	2	7 (−2.0)	0	0
Phytases	24	1.1	2 (5.7)	1 (2.9)	13 (2.3)	1	7 (−2.5)	0	0
Multi-enzymes	29	3.3	10 (9.0)	0	12 (1.8)	2	5 (−3.3)	0	0

^1^ Significant (Sig.; *p* ≤ 0.05), tendency (0.05 < *p* ≤ 0.10), and non-significant (NS.; *p* > 0.10). ^2^ Average of the % of difference of all the comparisons. ^3^ Number outside of the parentheses represents the number of comparisons. Number inside the parentheses represents the average of the percentage of difference of these comparisons.

**Table 3:** Summary of the effects of feed additives on grow–finish pig BF ^1,3^.

			Positive			Neutral	Negative		
Item	Comparisons, n	Difference, % ^2^	Sig.	Tendency	NS.		NS.	Tendency	Sig.
Acidifiers	24	−0.6	0	0	14 (2.6)	2	5 (−3.2)	0	3 (−12)
Essential oils	14	−2.7	0	0	5 (3.7)	0	6 (−5.5)	0	3 (−2.7)
DFM	21	−1.5	1 (16.8)	0	6 (7.1)	0	9 (−6.3)	3 (−2.9)	2 (−13.1)
Yeasts	21	−3.1	0	0	12 (4.1)	1	8 (−14.4)	0	0
Copper	73	−1.4	0	0	24 (3.5)	9	36 (−4.1)	1 (−5.4)	3 (−10.3)
Zinc	19	−0.6	1 (13.1)	0	5 (1.3)	2	11 (−2.9)	0	0
Betaine	32	−1.7	0	0	13 (2)	0	16 (−2.9)	0	3 (−10.7)
Chromium	133	−3.9	2 (8)	5 (6.3)	42 (4.2)	2	53 (−6.4)	7 (−12.4)	22 (−14.4)
CLA	59	−7.0	0	0	14 (4)	2	16 (−6.1)	5 (−6.5)	22 (−15.4)
L-carnitine	22	−3.4	3 (4)	2 (1.4)	1 (1.9)	1	6 (−5.7)	7 (−4.8)	2 (−12.5)
Carbohydrases	57	−0.4	2 (4.1)	1 (4.8)	18 (4)	7	29 (−3.7)	0	0
Proteases	13	−0.1	0	3 (3.7)	3 (4.5)	1	6 (−4.4)	0	0
Phytases	14	−0.2	1 (8.3)	0	6 (1.7)	2	5 (−4.2)	0	0
Multi-enzymes	12	2.8	0	0	6 (10.4)	0	5 (−3.8)	0	1 (−10.2)

^1^ Significant (Sig.; *p* ≤ 0.05), tendency (0.05 < *p* ≤ 0.10), and non-significant (NS.; *p* > 0.10). ^2^ Average of the % of difference of all the comparisons. ^3^ Number outside of the parentheses represents the number of comparisons. Number inside the parentheses represents the average of the percentage of difference of these comparisons.

**Table 4:** Summary of the effects of feed additives on grow–finish pig percentage lean ^1,3^.

			Positive			Neutral	Negative		
Item	Comparisons, n	Difference, % ^2^	Sig.	Tendency	NS.		NS.	Tendency	Sig.
Acidifiers	24	−0.5	0	0	9 (0.9)	0	15 (−1.4)	0	0
Essential oils	9	0.9	3 (2.5)	0	3 (1.2)	1	2 (−1.5)	0	0
DFM	13	1.0	0	1 (1.8)	9 (1.8)	0	3 (−1.8)	0	0
Yeasts	8	1.0	0	3 (0.8)	2 (4.9)	0	2 (−1.3)	1 (−1.2)	0
Copper	25	1.6	2 (1.1)	0	16 (2.8)	0	7 (−1.1)	0	0
Zinc	14	0.9	0	0	12 (1.1)	1	1 (−0.4)	0	0
Betaine	25	2.0	1 (5.2)	0	15 (3.6)	1	8 (−1.2)	0	0
Chromium	105	1.6	20 (6.6)	1 (5)	43 (1.9)	3	36 (−1.2)	0	2 (−4.1)
CLA	37	2.6	14 (4.9)	0	16 (1.9)	0	7 (−0.6)	0	0
L-carnitine	20	1.1	4 (3.8)	2 (1.5)	7 (1.5)	1	3 (−0.7)	0	3 (−1.3)
Carbohydrases	55	0.3	1 (5.6)	0	28 (1.1)	4	20 (−0.8)	0	2 (−0.7)
Proteases	13	0.0	0	0	6 (1.1)	1	6 (−1.1)	0	0
Phytases	9	0.0	0	0	4 (0.6)	0	5 (−0.5)	0	0
Multi-enzymes	9	0.7	1 (4.4)	0	6 (0.6)	0	2 (−0.9)	0	0

^1^ Significant (Sig.; *p* ≤ 0.05), tendency (0.05 < *p* ≤ 0.10), and non-significant (NS.; *p* > 0.10). ^2^ Average of the % of difference of all the comparisons. ^3^ Number outside of the parentheses represents the number of comparisons. Number inside the parentheses represents the average of the percentage of difference of these comparisons.

**Table 5:** Summary of the effects of feed additives on grow–finish pig longissimus muscle area/loin depth ^1,3^.

			Positive			Neutral	Negative		
Item	Comparisons, n	Difference, % ^2^	Sig.	Tendency	NS.		NS.	Tendency	Sig.
Acidifiers	11	1.6	2 (6.3)	0	7 (2.6)	0	2 (−6.9)	0	0
Essential oils	10	1.9	3 (7.1)	0	4 (1)	0	3 (−2.3)	0	0
DFM	19	1.5	1 (10.9)	0	14 (2)	1	3 (−3.4)	0	0
Yeasts	17	1.4	0	0	10 (3.6)	0	7 (−1.9)	0	0
Copper	62	2.3	5 (4.4)	0	43 (3.4)	2	11 (−1.4)	0	1 (−7.5)
Zinc	15	0.2	0	0	11 (0.9)	0	4 (−1.5)	0	0
Betaine	24	−0.2	0	1 (6.3)	10 (1.9)	0	10 (−2.2)	0	3 (−2.3)
Chromium	125	3.1	23 (13.9)	0	61 (3.2)	2	38 (−3)	0	1 (−11.6)
CLA	38	0.9	6 (7.6)	1 (3.7)	14 (3)	0	15 (−3)	1 (−4.8)	1 (−5.9)
L-carnitine	21	2.4	1 (6.3)	0	13 (4.4)	1	6 (−2.3)	0	0
Carbohydrases	38	1.1	0	0	20 (3.3)	1	17 (−1.5)	0	0
Proteases	9	−2	0	0	3 (2.4)	0	6 (−4.1)	0	0
Phytases	11	−1.4	0	0	7 (1.7)	0	4 (−6.9)	0	0
Multi-enzymes	9	0.3	1 (11.3)	0	3 (1.8)	0	5 (−2.8)	0	0

^1^ Significant (Sig.; *p* ≤ 0.05), tendency (0.05 < *p* ≤ 0.10), and non-significant (NS.; *p* > 0.10). ^2^ Average of the % of difference of all the comparisons. ^3^ Number outside of the parentheses represents the number of comparisons. Number inside the parentheses represents the average of the percentage of difference of these comparisons.

## Data Availability

Not applicable.

## Supplementary Materials

The following supporting information can be downloaded at: https://www.mdpi.com/article/10.3390/ani13020200/s1.

## Abbreviations

AA = amino acid; ADG = average daily gain; ADFI = average daily feed intake; BF = backfat; BW = body weight; CLA = conjugated linoleic acid; CP = crude protein; DDGS = distiller’s dried grains with solubles; DFM = direct-fed microbial; DM = dry matter; EO = Essential oils; G:F = gain-to-feed ratio; HCW = hot carcass weight; LD = loin depth; LMA = loin muscle area; MOS = mannan oligosaccharide; NE = net energy; % lean = Percentage lean; RAC = ractopamine; SBM = soybean meal; SID = standardized ileal digestible.
