# Supplementary material for: Effects of Various Feed Additives on Finishing Pig Growth Performance and Carcass Characteristics: A Review
Source: Animals (Basel). 2023 Jan 5;13(2):200. doi: 10.3390/ani13020200 (PMC9854424; doi:10.3390/ani13020200)
Supplement: Supplementary file 1 [file animals-13-00200-s001.zip › animals-2116661-supplementary.pdf]

Effects of Various Feed Additives on Finishing Pig Growth Performance and Carcass Characteristics: A Review—Supplemental Material

Zhong-Xing Rao 1, Mike D. Tokach 1, Jason C. Woodworth 1, Joel M. DeRouchey 1, Robert D. Goodband 1,* and Jordan T. Gebhardt 2

[1] Department of Animal Sciences and Industry, College of Agriculture, Kansas State University, Manhattan, KS, USA 66506

[2] Department of Diagnostic Medicine/Pathobiology, College of Veterinary Medicine, Kansas State University, Manhattan, KS, USA 66506

* Corresponding author: Goodband@ksu.edu

1. Feed Additives – Health

This section discusses the feed additives that have the potential to improve growth performance and carcass characteristics by enhancing the health status of grow-finish pigs. The feed additives discussed are acidifiers, essential oils, DFM, yeasts, Cu, and Zn.

1.1. Acidifiers

There were 32 research articles for acidifiers with 68 comparisons from 16 countries during the grow-finish or finishing period which met the requirements for inclusion. Of these, 68 comparisons reported growth performance data and 42 comparisons reported carcass data. Most acidifiers collected for this review were organic acids that were in the form of short-chain fatty acids (**SCFA**; 39 comparisons), medium-chain fatty acids (**MCFA**; 1 comparison), and benzoic acid (10 comparisons), and were added alone or in combinations (SCFA and MCFA; 18 comparisons) from 0.05 to 5.0% in the diets.

1.1.1. Growth performance - Acidifiers

Average daily gain significantly increased ($P \leq 0.05$) in 18 comparisons (average of 5.8%) and significantly decreased ($P \leq 0.05$) in 4 comparisons (average of 10.8%) compared to control pigs (Table S1). The greatest proportion of the comparisons found no evidence of difference ($P > 0.10$) in ADG (46 comparisons). Of these, ADG was numerically increased ($P > 0.10$) in 31 comparisons (average of 3.4%) and numerically decreased in 15 comparisons (average of 3.4%) compared to control pigs. Feed efficiency significantly increased ($P \leq 0.05$) in 13 comparisons (average of 6.4%) and significantly decreased ($P \leq 0.05$) in 1 comparison (9.7%) compared to control pigs. The greatest proportion of the comparisons found no evidence of difference ($P > 0.10$) in ADG (51 comparisons). Of these, G:F was numerically increased ($P > 0.10$) in 40 comparisons (average of 3.8%) and numerically decreased in 9 comparisons (average of 3.1%)

compared to control pigs. By comparing different acid types, acid blends and benzoic acids improved ADG more than SCFAs, while acid blends and SCFA improved G:F more than benzoic acid. Compared to control pigs, those fed acidifiers had 4.1% (18 comparisons), 2.8% (10 comparisons), and 0.3% (39 comparisons) improvement in ADG when fed acid blends, benzoic acid, and SCFA, respectively. Feed efficiency was improved by 4.2% (18 comparisons), 2.1% (10 comparisons), and 2.9% (39 comparisons) in pigs fed acid blends, benzoic acid, and SCFA, respectively, compared to control pigs. There were not enough data to support whether different types of basal diets and inclusion levels affected the response to acidifiers for ADG and G:F in grow-finish pig diets. In summary, feeding acidifiers has the potential to improve growth performance.

Table S1. Studies on the effects of dietary acidifiers on growth performance.

Author	Country	Acids	Inclusion, %	Sig.	Difference, %	
					ADG	G:F
Thacker and Bowland (1980)	Canada	Propionic acid	3.0	ns	-2.7	6.3
			6.0	ADG	-12.2	4.5
			9.0	ADG	-14.9	10.8
Thacker and Bowland (1981)	Canada	Propionic acid	3.5	ns	1.3	7.1
			7.0	ns	-3.8	5.9
		Calcium propionate	3.5	ns	4.8	6.6
			7.0	ns	-8.3	-6.8
Thacker et al. (1981)	Canada	Propionic acid	5.0[4]	ADG, G:F	-8.1	8.8
			5.0[4]	ADG	-7.9	6.1
Giesting and Easter (1985)	USA	Fumaric acid	1.5	ns[2]	2.5	-2.7
			3.0		7.6	0.0
Thacker et al. (1992)	Canada	Propionic acid	2.5	ns	-1.2	4.1
Baustad (1993), Exp. 1	Norway	Formic acid	0.6	ADG, G:F	11.4	11.3
			1.2	ns	5.3	6.2
Baustad (1993), Exp. 2	Norway	Formic acid	0.6	ADG, G:F	7.0	7.0
Baustad (1993), Exp. 3	Norway	Formic acid	0.6	ns	3.8	3.6
Krause et al. (1994)	USA	Fumaric acid	2.5	ns	2.3	3.6
Siljander-Rasi et al. (1998)	Finland	Formic acid	0.8	ns	1.4	1.0
Partanen et al. (2002)	Finland	Formic acid	0.8	G:F	4.7	4.9
		Formic acid and sorbate	0.8	ADG, G:F	8.6	9.9
Canibe et al. (2005)	Denmark	Formic acid	1.8	ns	9.0	5.4
Jansons and Nudiens (2005)	Lativia	Formic acid, acetic acid, citric acid, and phosphoric acid	0.6/0.4/0.3	ADG	5.9	n/a
Bühler et al. (2006)	Switzerland	Benzoic acid	1.0	ns	4.0	1.7
Campbell et al. (2006)	Ireland	Acid blend	0.3	ns	-5.4	-7.5
		Fumaric acid	0.2	ns	-8.1	-6.3
Partanen et al. (2006)	Finland	Sorbate-coated formic acid	0.3	ns	4.9	3.7
			0.6	ADG	6.4	3.7
			1.2	ADG, G:F	5.6	7.3
		Formic acid and lactic acid	0.3	ADG, G:F	6.4	5.5
			0.6	G:F	4.6	4.6
			1.2	G:F	5.4	5.0
Eisemann and Heugten (2007)	USA	Formic acid, ammonium formate	1.2/1.0	ns	-0.9	2.4
			1.0/0.8	ns	-0.1	5.2
			0.8/0.6	ns	0.9	3.9
			1.0	ns	2.9	3.4
			0.8	ns	-1.0	2.5

			0.6	ns	-1.4	2.3
Øverland et al. (2007)	Norway	Formic acid	1.0	ns	1.8	3.4
		Benzoic acid	0.85	ns	2.3	3.9
		Sorbic acid	0.85	ns	2.4	4.4
		Fat coated Ca-butyrate	1.2	ns	7.2	0.9
		Inulin coated Ca-butyrate	1.5	ns	-2.9	0.5
Guy et al. (2008)	UK	Formic acid and propionic acid	0.7/0.6/0.5/0.0.3	ns	-1.5	-0.9
		Formic acid, fumaric acid, and propionic acid	1.0/0.8/0.6/0.5/0.4	ns	-0.3	-1.3
Kijparkorn et al. (2009)	Thailand	Formic acid, lactic acid, citric acid, fumaric acid	0.4	G:F	-12.5	-9.7
Thacker and Haq (2009)	Canada	Propionic acid and acetic acid	1.0	ns	4.8	-0.7
Jansons et al. (2011)	Lativia	Formic acid, acetic acid, citric acid, and phosphoric acid	0.6/0.4/0.3[4]	ADG	6.2	n/a
			0.6/0.4/0.3[4]	ns	-1.3	n/a
Upadhaya et al. (2014)	South Korea	Fumaric acid, citric acid, malic acid, MCFA (capric and caprylic acid)	0.1	ADG[2]	3.9	2.8
			0.2		6.0	3.7
Cho et al. (2015)	South Korea	Benzoic acid	0.5	ns	0.1	-1.1
Giannenas et al. (2016)	Greece	Benzoic acid	0.5	ns	2.9	7.1
Zhai et al. (2017)	China	Benzoic acid	0.3	ADG[2], G:F[2]	5.9	3.1
			0.5		5.2	3.1
Lei et al. (2018)	South Korea	Fumaric acid, citric acid, malic acid, MCFA (capric and caprylic acid)	0.05	ns	3.1	4.7
			0.1	ns	5.4	6.3
Nguyen Thi (2018)	Vietnam	Fumaric acid, lactic acid, calcium formate, and phosphoric acid	0.2	ADG	5.6	4.3
Morel et al. (2019)	New Zealand	Benzoic acid	0.5	ns	0.5	0.7
		Butyrate	0.15	ns	2.4	0.3
Nguyen et al. (2019)	South Korea	Fumaric acid, citric acid, malic acid, MCFA (capric and caprylic acid)	0.1	ADG	4.1	4.5
			0.2	ADG, G:F	4.5	5.0
O' Meara et al. (2020)	Ireland	Benzoic acid	0.25	ns[2]	2.9	-0.9
			0.5		1.1	1.8
Tran Thi Bich et al. (2020)	Thailand	Formic acid, acetic acid, lactic acid, propionic acid, citric acid, and sorbic acid) and MCFAs	0.2	ADG, G:F	5.3	8.1

Muniyappan et al. (2021)	South Korea	Fumaric acid, citric acid, phosphoric acid, and malic acid	0.05	ADG[2]	2.5	1.4
			0.1		3.3	1.8
Tutida et al. (2021)	Brazil	Lactic, citric, and ascorbic acid	0.1/0.05	ns	0.0	0.0

[1]Significant level at $P \leq 0.05$. Difference is calculated as [(treatment value – control value) / control value] * 100%.

[2]Polynomial contrasts were used for statistical analysis.

[3]Significant level at $0.05 < P \leq 0.10$.

[4]For experiments using factorial treatment structures, if the interaction of factors of either interested variable was observed the effect of the feed additive within each level of the other factor is included within the database.

1.1.2. *Carcass Characteristics - Acidifiers*

Back-fat significantly decreased ($P \leq 0.05$) in 3 comparisons (average of 12%) compared to control pigs. The greatest proportion of the comparisons found no evidence of difference ($P > 0.10$) in BF (21 comparisons; Table S2). Of these, BF was numerically increased ($P > 0.10$) in 14 comparisons (average of 2.6%) and numerically decreased in 5 comparisons (average of 3.2%) compared to control pigs. For percentage lean, all comparisons found no evidence of difference ($P > 0.10$) in percentage lean. Of these, percentage lean was numerically increased ($P > 0.10$) in 9 comparisons (average of 0.9%) and numerically decreased in 15 comparisons (average of 1.4%) compared to control pigs. Loin muscle area/depth significantly increased ($P \leq 0.05$) in 2 comparisons (average of 6.3%) compared to control pigs. The greatest proportion of the comparisons found no evidence of difference ($P > 0.10$) in LAM/LD (9 comparisons). Of these, LMA/LD was numerically increased ($P > 0.10$) in 7 comparisons (average of 2.6%) and numerically decreased in 2 comparisons (average of 6.9%) compared to control pigs. These results could be expected because the mechanisms do not directly affect the protein and lipid metabolism. Also, it appears that acidifiers' impacts on ADG and G:F were not great enough to affect carcass characteristics.

Table S2. Studies on the effects of dietary acidifiers on carcass characteristics.

Author	Country	Acidifiers	Inclusion, %	Sig.	Difference, %			
					Yield	BF	percentage lean	LMA/LD
Thacker and Bowland (1980)	Canada	Propionic acid	3.0	ns	0.1	-2.4	n/a	n/a
			6.0	BF	2.2	-5.9	n/a	n/a
			9.0	BF	-3.5	-15.3	n/a	n/a
Thacker and Bowland (1981)	Canada	Propionic acid	3.5	ns	-0.4	0.9	n/a	n/a
			7.0	ns	-0.6	-2.6	n/a	n/a
		Calcium propionate	3.5	ns	-0.6	-6.6	n/a	n/a
			7.0	BF	-2.7	-14.8	n/a	n/a
Thacker et al. (1992)	Canada	Propionic acid	2.5	ns	-0.6	n/a	-0.6	n/a
Baustad (1993), Exp. 1	Norway	Formic acid	0.6	ns	n/a	n/a	-0.5	n/a
			1.2	ns	n/a	n/a	-1.6	n/a
Baustad (1993), Exp. 2	Norway	Formic acid	0.6	ns	n/a	n/a	4.2	n/a
Baustad (1993), Exp. 3	Norway	Formic acid	0.6	ns	n/a	n/a	0.8	n/a
Partanen et al. (2002)	Finland	Formic acid	0.8	ns	0.5	1.3	0.2	n/a
		Formic acid and sorbate	0.8	ns	0.0	1.3	0.3	n/a
Campbell et al. (2006)	Ireland	Acid blend	0.3	ns	-0.3	n/a	n/a	n/a
		Fumaric acid	0.2	ns	0.5	n/a	n/a	n/a
Partanen et al. (2006)	Finland	Sorbate-coated formic acid	0.3	ns	-0.1	6.2	-1.0	n/a
			0.6	ns	-0.8	0.0	-0.2	n/a
			1.2	ns	0.1	1.6	-0.3	n/a
		Formic acid and lactic acid	0.3	ns	-0.4	1.6	-0.3	n/a
			0.6	ns	0.0	2.3	-0.3	n/a
			1.2	ns	0.5	0.8	0.5	n/a
Øverland et al. (2007)	Norway	Formic acid	1.0	ns	0.4	n/a	-2.7	n/a
		Benzoic acid	0.85	Yield	2.3	n/a	-1.8	n/a
		Sorbic acid	0.85	ns	-0.3	n/a	-2.8	n/a
		Fat coated Ca-butyrate	1.2	ns	-0.1	n/a	-2.9	n/a
		Inulin coated Ca-butyrate	1.5	ns	-0.4	n/a	-3.6	n/a
Thacker and Haq (2009)	Canada	Propionic and acetic acid	1.0	ns	0.0	14.4	-1.7	4.1
Upadhaya et al. (2014)	South Korea	Fumaric acid, citric acid, malic acid, MCFA (capric and caprylic acid)	0.1	LMA[2]	n/a	n/a	n/a	4.6
			0.2		n/a	n/a	n/a	8.1
Nguyen Thi (2018)	Vietnam	Fumaric acid, lactic acid, calcium formate, and phosphoric acid	0.2	ns	-0.8	0.5	n/a	2.5
Morel et al. (2019)		Benzoic acid	0.5	ns	0.1	0.0	n/a	1.4

	New Zealand	Butyrate	0.15	ns	-0.4	1.1	n/a	3.4
Nguyen et al. (2019)	South Korea	Fumaric acid, citric acid, malic acid, MCFA (capric and caprylic acid)	0.1	ns	n/a	n/a	n/a	-7.2
			0.2	ns	n/a	n/a	n/a	-6.5
O′ Meara et al. (2020)	Ireland	Benzoic acid	0.25	ns[2]	-0.1	3.6	-0.5	1.3
			0.5		-0.5	-0.7	0.5	2.6
			1.0		-0.9	-3.6	1.1	3.0
Muniyappan et al. (2021)	South Korea	Fumaric acid, citric acid, phosphoric acid, and malic acid	0.05	ns[2]	n/a	1.2	0.5	n/a
			0.1		n/a	2.2	0.1	n/a

[1]Significant level at $P \leq 0.05$. Difference is calculated as [(treatment value – control value) / control value] * 100%.
[2]Polynomial contrasts were used for statistical analysis.

1.2. *Essential Oils (EO)*

There were 13 research articles for EO with 20 comparisons from 6 countries during the grow-finish or finishing period with added dietary levels of 0.003 to 0.1%. Of these, 20 comparisons reported growth performance data and 17 comparisons reported carcass data. Essential oils used in these experiments were extracted from caraway, citrus, cinnamon, Chinese cinnamon, oregano, clove, clover, rosemary, fenugreek seed, eucalyptus, lemon, garlic, and *Eucommia ulmoides*. Because of the similar antibacterial properties between essential oils and acids, these two additives are sometimes blended as a single additive. Therefore, 5 more articles (5 experiments) from 4 countries with blended additives (EO and acids) were also included.

1.2.1. *Growth Performance - Essential Oils*

Average daily gain significantly increased ($P \leq 0.05$) in 10 comparisons (average of 9.9%) compared to control pigs (Table S3). Half of the studies found no evidence of difference ($P > 0.10$) in ADG (10 comparisons). Of these, ADG was numerically increased ($P > 0.10$) in 6 comparisons (average of 3.8%) and numerically decreased ($P > 0.10$) in 3 comparisons (average of 1.7%) compared to control pigs. Feed efficiency significantly increased ($P \leq 0.05$) in 7 comparisons (average of 10.9%) and tended to increase ($0.05 < P \leq 0.10$) in 1 comparison (4.5%) compared to control pigs. Half of the studies found no evidence of difference ($P > 0.10$) in G:F (9 comparisons). Of these, G:F was numerically increased ($P > 0.10$) in 6 comparisons (average

of 3.5%) and numerically decreased ($P > 0.10$) in 2 comparisons (average of 1.5%) compared to control pigs. Overall, the results suggest that EO had positive effects on ADG and G:F (80 and 82% of all the comparisons). Moreover, the beneficial effects of EO were significant ($P < 0.10$) for ADG and G:F in 50 and 57% of all the comparisons, respectively. For EO and acid blends, there are only 7 comparisons for both ADG (average of 1.9% improvement) and G:F (average of 2.2% improvement). Of these, 71% of the comparison where pigs fed the additive had increased ADG (average of 3.8%) and G:F (average of 3.7%), and 29% of the comparisons had reduced ADG (average of 2.9%) and G:F (average of 1.5%) compared to control. There were insufficient data to support whether different types of basal diets and inclusion levels affected EO response for ADG and G:F. In summary, adding EO alone or in combination with acids has the potential to improve growth performance. However, there was only a small amount of research on EO's effect on growth performance, and only three studies were conducted in the US; therefore, the use of EO may not be beneficial in US-based conditions. More experiments are needed to determine the effect of including EO in the diets of grow-finish pigs.

Table S3. Studies on the effects of dietary essential oils (EO) with or without acids on growth performance.

Author	Country	Additive	Inclusion, %	Sig.[1]	Difference, %[1]	
					ADG	G:F
Essential oils						
Onibala et al. (2001)	Indonesia	Oregano EO	0.0025	ADG, G:F	4.1	5.8
		Thyme EO	0.0025	ADG, G:F	8.8	6.5
		Garlic EO	0.0025	ADG, G:F	5.9	7.7
Yan et al. (2010)	South Korea	Thyme, rosemary, and oregano EO	0.01	ADG	6.6	6.6
Simitzis et al. (2010)	Greece	Oregano EO	0.025	ns	11.1	n/a
			0.05	ns	2.6	n/a
			0.1	ns	3.7	n/a
Zhou et al. (2016)	China	*Eucommia ulmoides* oliver leaf polyphenolic extract	0.08	ADG, G:F	18.8	19.9
Zou et al. (2016)	China	*Oregano EO*	0.0025	ADG, G:F	18.6	15.7
Li et al. (2017)	South Korea	Cinnamon, oregano, clove, thyme, and rosemary EO	0.05	ns	3.8	4.1
			0.05	ns	1.0	2.0
Soto et al. (2017)	USA	Caraway, garlic, thyme, and cinnamon	0.020	ns	-0.5	1.3
		Oregano, citrus, and anise	0.013	ns	0.5	1.0
		Caraway, garlic, thyme, cinnamon, oregano, citrus, and anise	0.033	ns	0.0	-0.3
Zou et al. (2017)	China	Oregano EO	0.0025	ADG, G:F	10.2	9.4
Cheng et al. (2018)	China	Oregano EO	0.025	ADG, G:F	10.6	11.0
Lan and Kim (2018)	South Korea	Fenugreek seed, clover, and Chinese cinnamon EO	0.01	ADG	5.0	5.9
Lowell et al. (2018)	USA	Oregano EO	0.025	ns	-2.9	0.0
Huang et al. (2021)	China	Eucalyptus, oregano, thyme, lemon, garlic EO	0.02	ADG, G:F[3]	10.3	4.5
Tutida et al. (2021)	Brazil	Thymol and Carvacrol	0.100	ns	-1.6	-2.6
Essential oils and Acidifier Blends						
Cho et al. (2014)	South Korea	Citric acids, sorbic acid, and EOs (thymol and vanillin)	0.025<br>0.05	ADG[2]	4.3<br>4.0	3.0<br>3.0
Walia et al. (2017)	Ireland	Formic acid, citric acid, and EOs (citrus fruit extract, cinnamon, oregano, thyme, and capsicum)	0.4	ns	-4.6	-2.5
Oh et al. (2019)			0.1/0.025[5]	ADG[2], G:F[2]	2.2	2.7

	South Korea	Citric acids, sorbic acid, and EOs (thymol and vanillin)	0.2/0.05[5]		5.4	5.9
Resende et al. (2020)	Brazil	Benzoic acid and EOs (thymol, 2-methoxyphenol, and eugenol)	0.3	ADG	3.3	3.8
Hutchens et al. (2021)	USA	Citric acids, sorbic acid, and EOs (thymol and vanillin)	0.3/0.1/0.054	ns	-1.2	-0.5

[1]Significant level at $P \leq 0.05$. Difference is calculated as [(treatment value – control value) / control value] * 100%.

[2]Polynomial contrasts were used for statistical analysis.

[3]Significant level at $0.05 < P \leq 0.10$.

[4]Feed additive was added at 0.3, 0.1% in nursery phase 1 and 2, respectively, and 0.05% in grow-finish phase.

[5]For the low inclusion treatment, feed additive was added at 0.1% in nursery phase and 0.025% in grow-finish phase. For the high inclusion treatment, feed additive was added at 0.2% in nursery phase and 0.05% in grow-finish phase.

1.2.2. *Carcass Characteristics - Essential Oils*

Back-fat significantly decreased ($P \leq 0.05$) in 3 comparisons (average of 2.7%) compared to control pigs (Table S4). The greatest proportion of the comparisons found no evidence of difference ($P > 0.10$) in BF (11 comparisons). Of these, BF was numerically increased ($P > 0.10$) in 5 comparisons (average of 3.7%) and numerically decreased ($P > 0.10$) in 6 comparisons (average of 5.5%) compared to control pigs. Percentage lean significantly increased ($P \leq 0.05$) in 3 comparisons (average of 2.5%) compared to control pigs. The greatest proportion of the comparisons found no evidence of difference ($P > 0.10$) in percentage lean (6 comparisons). Of these, percentage lean was numerically increased ($P > 0.10$) in 3 comparisons (average of 1.2%) and numerically decreased ($P > 0.10$) in 2 comparisons (average of 1.5%) compared to control pigs. Loin muscle area/depth significantly increased ($P \leq 0.05$) in 3 comparisons (average of 7.1%) compared to control pigs. The greatest proportion of the comparisons found no evidence of difference ($P > 0.10$) in LMA/LD (7 comparisons). Of these, LMA/LD was numerically increased ($P > 0.10$) in 4 comparisons (average of 1.0%) and numerically decreased ($P > 0.10$) in 3 comparisons (average of 2.3%) compared to control pigs. For essential oils and acid blends, there are only 3 experiments, and the effects on carcass characteristics were small and not statistically significant [BF (average of 0.5% improvement); percentage lean (average of 1.7% improvement); and LMA (average of 0.1% improvement)]. These results suggest that adding EO alone had some positive effects on carcass characteristics, which may be due to the

improvement in growth performance. However, there was only a small amount of research on EO's effect on carcass characteristics, and only two research were conducted in the US. Therefore, more experiments are needed to determine the effect of including EO in the diets of grow-finish pigs.

Table S4. Studies on the effects of dietary essential oils (EO) with or without acids on carcass characteristics.

Author	Country	Additive	Inclusion, %	Sig.[1]	Difference, %[1]			
					Yield	BF	percentage lean	LMA/LD
Essential oils								
Onibala et al. (2001)	Indonesia	Oregano EO	0.0025	Yield, BF, percentage lean	3.5	-7.5	2.4	n/a
		Thyme EO	0.0025	Yield, BF, percentage lean	3.8	-7.8	2.6	n/a
		Garlic EO	0.0025	Yield, BF, percentage lean	3.3	-7.7	2.5	n/a
Simitzis et al. (2010)	Greece	Oregano EO	0.025	ns	-0.6	4.1	n/a	n/a
			0.05	ns	0.1	-3.2	n/a	n/a
			0.1	ns	0.5	1.8	n/a	n/a
Yan et al. (2010)	South Korea	Thyme, rosemary, and oregano EO	0.01	LMA	n/a	n/a	n/a	12.3
Zhou et al. (2016)	China	Eucommia ulmoides oliver leaf polyphenolic extract	0.08	ns	0.8	-9.9	-2.5	-6.3
Zou et al. (2016)	China	Oregano EO	0.0025	Yield	8.2	-0.5	n/a	n/a
Li et al. (2017)	South Korea	Cinnamon, oregano, clove, thyme, and rosemary EO	0.05	LMA	n/a	n/a	n/a	5.4
			0.05	LMA	n/a	n/a	n/a	3.7
Soto et al. (2017)	USA	Caraway, garlic, thyme, and cinnamon	0.020	ns	0.3	6.3	-0.4	0.8
		Oregano, citrus, and anise	0.013	ns	0.0	3.1	0.0	1.2
		Caraway, garlic, thyme, cinnamon, oregano, citrus, and anise	0.033	ns	0.1	3.1	0.2	2.0
Cheng et al. (2018)	China	Oregano EO	0.025	ns	-0.8	-14.2	2.8	0.0
Lowell et al. (2018)	USA	Oregano EO	0.025	Yield	-0.8	-3.9	0.7	-0.1
Huang et al. (2021)	China	Eucalyptus, oregano, thyme, lemon, garlic EO	0.02	ns	0.3	-1.4	n/a	-0.6
Essential oils and Acidifier Blends								
Cho et al. (2014)			0.025	ns[2]	n/a	n/a	n/a	-2.7

	South Korea	Citric acids, sorbic acid, and EOs (thymol and vanillin)	0.05		n/a	n/a	n/a	-2.4
Walia et al. (2017)	Ireland	Formic acid, citric acid, and EOs (citrus fruit extract, cinnamon, oregano, thyme, and capsicum)	0.4	Yield, BF[3], percentage lean, LMA	-1.1	-7.0	2.0	4.0
Oh et al. (2019)	South Korea	Citric acids, sorbic acid, and EOs (thymol and vanillin)	0.1/0.025[4]	percentage lean[2,3]	n/a	4.2	0.5	0.1
			0.2/0.05[4]		n/a	4.2	2.6	1.5

[1]Significant level at $P \leq 0.05$. Difference is calculated as [(treatment value – control value) / control value] * 100%.
[2]Polynomial contrasts were used for statistical analysis.
[3]Significant level at $0.05 \leq P < 0.10$.
[4]For the low inclusion treatment, feed additive was added at 0.1% in nursery phase and 0.025% in grow-finish phase. For the high inclusion treatment, feed additive was added at 0.2% in nursery phase and 0.05% in grow-finish phase.

1.3. *Direct-Fed Microbials (DFM)*

There were 48 research articles for DFM with 79 comparisons from 14 countries during the grow-finish or finishing period which met the requirements for inclusion. Of these, 73 comparisons reported growth performance data, and 33 comparisons reported carcass data. Most strains of DFM used in the studies were *Bacillus spp., Lactobacillus spp.,* and *Enterococcus faecium.* A DFM additive could contain a single or several strains of microbials. In addition, comparisons were also included when yeast (*Saccharomyces cerevisiae*) was added with other microbials as a blended DFM product. The effect of the single addition of yeast in diets was discussed in the yeast section.

1.3.1. *Growth Performance - DFM*

Average daily gain significantly increased ($P \leq 0.05$) in 25 comparisons (average of 6.3%), tended to increase ($0.05 < P \leq 0.10$) in 2 comparisons (average of 3.9%), and significantly decreased ($P \leq 0.05$) in 1 comparison (5.8%) compared to control pigs (Table S5). The greatest proportion of the comparisons found no evidence of difference ($P > 0.10$) in ADG (43 comparisons). Of these, ADG was numerically increased ($P > 0.10$) in 30 comparisons (average of 3.6%) and numerically decreased ($P > 0.10$) in 13 comparisons (average of 2.3%) compared to control pigs. in pigs fed DFM. Feed efficiency significantly increased ($P \leq 0.05$) in 18 comparisons (average of 6.1%) and tended to increase ($0.05 < P \leq 0.10$) in 3 comparisons (average of 3%) compared to control pigs. The greatest proportion of the comparisons found

no evidence of difference ($P > 0.10$) in G:F (45 comparisons). Of these, G:F was numerically increased ($P > 0.10$) in 32 comparisons (average of 3.9%) and numerically decreased ($P > 0.10$) in 11 comparisons (average of 2.2%) compared to control pigs. Overall, the results suggest that DFM positively affected ADG and G:F (80% of all the comparisons). Moreover, DFM showed positive statistical improvement ($P < 0.10$) in 38 and 32% of all the comparisons for ADG and G:F, respectively. Similarly, Zimmermann et al. [50] conducted a meta-analysis and found probiotics significantly improved ADG and G:F of weaned piglets and finishing pigs. There were insufficient data to support whether different types of basal diets affected the response to DFM for ADG and G:F in grow-finish pigs. The effect of strain and inclusion level of DFM cannot be discussed because most studies used a blend of several microbials with varying concentrations. In summary, DFM has the potential to improve growth performance (3.3% improvement for ADG and G:F) of grow-finish pigs. However, there were relatively fewer US-based studies for DFM; therefore, the effects of DFM in US-based conditions may not be the same as what we discussed in this section.

Table S5. Studies on the effects of DFM on growth performance.

Author	Country	DFM[4]	Inclusion, %	Sig.[1]	Difference, %[1]	
					ADG	G:F
Pollmann et al. (1980)	USA	*L. acidophilus*	0.05	ns	-1.2	-0.9
		Streptococcus faeciurn	0.05	ns	-1.2	-0.9
Harper et al. (1983)	USA	*L. acidophilus*	0.1/0.05	ADG	-5.8	-3.0
			0.05	ns	1.3	-1.4
Kim et al. (1998)	USA	*L. acidophilus*	0.05	ns	2.2	-0.4
Kyriakis et al. (2003)	Greece	*B. toyoi*	1.0/0.5/0.2×10[9] spores/kg	ADG	4.5	n/a
			1.0/0.5/0.2×10[9] spores/kg	ADG	8.3	n/a
Alexopoulos et al. (2004)	Greece	*B. licheniformis and B. subtilis*	0.04/0.02	ADG, G:F	2.2	4.5
			0.04/0.04	ADG, G:F	3.6	5.7
			0.04/0.06	ADG, G:F	3.6	5.3
Rekiel et al. (2005)	Poland	*Pediococcus acidilactici*	0.01	ns	-1.9	-1.9
Jukna et al. (2005)	Lithuania	*Saccharamyce cerevisiae, L. casei, L. acidophilus, Streptococcus faecium, B. subtilis*	0.20	ns	20.3	n/a
Shon et al. (2005)	South Korea	*L. reuteri and L. salivarius complex*	0.2	ns	3.2	1.4
Chen et al. (2006)	South Korea	*B. subtilis, B. coagulans, and L. acidophilus*	0.1	ns	5.3	3.7
			0.2	ADG	11.4	5.1
Chen et al. (2006)	South Korea	*Enterococcus faecium SF68*	0.1	ns[2]	4.9	2.0
			0.2		4.1	4.0
Davis et al. (2008)	USA	*B. lichenformis and B. subtilis*	0.05	G:F	0.6	3.0
Ko et al. (2008)	South Korea	*L. acidophilus, L. plantarum, B. subtilis, B. coagulans, and Saccharomyces cerevisiae*	0.5	ns	4.6	7.7
Ko and Yang (2008)	South Korea	*L. acidophilus, L. plantarum, B. subtilis, B. coagulans, and Saccharomyces cerevisiae*	0.1	ns	7.5	0.9
			0.5	ns	6.5	2.9
			1.0	ns	4.3	-1.4
Černauskienė et al. (2010), Exp. 1	Lithuania	*Enterococcus faecium*	10^{10} cfu/kg	ADG	3.1	-0.6
Černauskienė et al. (2010), Exp. 2	Lithuania	*Enterococcus faecium*	10^{10} cfu/kg	ns	1.5	3.0
Meng et al. (2010)	South Korea	*B. subtilis endospores and Clostridium butyricum*	0.2	ADG, G:F	7.5	7.9
Giang et al. (2011)	Vietnam	*B. subtilis*	0.3	ns	1.3	1.8
		B. subtilis and Saccharomyces boulardi	0.3	ns	2.6	2.9
		B., Saccharomyces boulardi, Enterococcus faecium, L. acidophilus, Pediococcus pentosaceus, and L. fermentum	0.3	ADG	5.2	5.3

Nitikanchana et al. (2011)	USA	*B. species*	0.2 × 10⁹ cfu/g<br>2 × 10⁹ cfu/g	ns[2]	-2.3<br>-1.4	0.8<br>0.4
Hossain et al. (2012)	South Korea	*L. acidophilus, L. plantarum, B. subtilis, B. coagulans, and Saccharomyces cerevisiae*	0.5	ADG	7.9	6.0
Cui et al. (2013)	China	*B. subtilis*	2.0	ADG, G:F	3.6	2.4
Kerr et al. (2013)	USA	*Pediococcus acidilactici*	0.011	ns	0.8	-1.5
		B. licheniformis and B. subtilis	0.05	ns	-1.1	-5.4
Liu et al. (2013)	China	*Yeasts, lactic acid-producing bacteria, and B. subtilis*	1.0	ns	3.2	2.9
Balasubramanian et al. (2016)	South Korea	*B. coagulans, B. licheniformis, B. subtilis, and Clostridium butyricum*	0.01<br>0.02	ADG[2], G:F[2]	2.7<br>3.2	4.9<br>5.2
Dowarah et al. (2016)	India	*L. acidophilus NCDC-15*	0.02	ADG, G:F	11.7	9.4
		Pediococcus acidilactici FT28	0.02	ADG, G:F	14.9	9.8
Giannenas et al. (2016)	Greece	*Enterococcus faecium*	0.0035	ns	3.0	6.8
Jørgensen et al. (2016)	Denmark	*B. licheniformis and B. subtilis*	0.04	ADG, G:F	3.3	1.9
Sarker et al. (2016)	South Korea	*L. acidophilus, L. plantarum, B. subtilis, B. coagulans, and Saccharomyces cerevisiae*	0.2	ns	-6.2	-7.2
			0.4	ns	-0.5	3.9
			0.8	ns	2.1	13.1
Nguyen et al. (2017)	South Korea	*Enterococcus faecium*	0.01	ADG, G:F	3.9	3.1
Tufarelli et al. (2017)	Italy	*Streptococcus thermophilus, Bifidobacterium animalis ssp. Lactis, L. acidophilus, L. helveticus, L. paracasei, L. plantarum, and L. brevis.*	100 mg/kg BW	ADG3	3.0	n/a
Balasubramanian et al. (2018)	South Korea	*B. coagulans, B. licheniformis, B. subtilis, and Clostridium butyricum*	0.01	ADG, G:F	6.0	8.4
			0.02	ADG, G:F	7.4	9.3
Bučko et al. (2018)	Slovak	*L. plantarum*	3g/day	ns	-1.2	0.4
Nguyen Thi (2018)	Vietnam	*B. subtilis, L. spp., Saccharomyces cerevisiae*	0.2	ADG	4.7	3.3
Samolińska et al. (2018)	Poland	*L. lactis, Carnobacterium divergens, L. casei, L. plantarum, and Saccharomyces cerevisiae*	0.05[5]	ns	2.2	2.2
			0.05[5]	ns	3.1	3.5
			0.05[5]	ns	2.5	3.0
Shi et al. (2018)	China	*B. subtilis and Devosia sp.*	0.2	ns	-3.2	10.9
			0.2	ADG	15.3	5.1
Lan and Kim (2019)	South Korea	*B. licheniformis and B. subtilis*	0.02<br>0.04<br>0.08	ns[2]	1.8<br>2.0<br>5.4	0.0<br>0.9<br>1.2
Wang and Kim (2019)	South Korea	*B. subtilis and P. farinosa*	0.1<br>0.2	ADG[2], G:F[2,3]	1.7<br>3.8	2.0<br>4.0
Peet-Schwering et al. (2020)	Netherlands	*B. amyloliquefaciens and B. subtilis*	0.04	G:F	1.4	0.5

Reszka et al. (2020)	Poland	*EM Carbon Bokash*	0.5/0.3	ns	1.4	0.8
Rybarczyk et al. (2020)	Poland	*Saccharomyces cerevisiae, L. casei, and L. plantarum*	0.30	ns	-5.9	n/a
			0.50	ns	-2.4	n/a
Frimpong et al. (2021)	Ghana	*L. sp., B. sp., and Saccharomyces cerevisiae*	0.1	ns	n/a	9.0
		L. sp., B. sp., Saccharomyces cerevisiae, and Paenibacillus polymyxa	0.15	ns	n/a	4.1
Grela et al. (2021)	Poland	*Lactococcus lactis, Carnobacterium divergens S1, L. casei, L. plantarum, and Sacharomyces cerevisiae*	0.1	ADG, G:F[3]	2.5	3.0
Kwak et al. (2021)	South Korea	*L. plantarum, L. fermentum, L. salivarius, Leuconostoc paramesenteroides, and B. subtilis, and B. licheniformis*	0.2	ADG[3], G:F	4.8	8.6
Pomorska-Mól et al. (2021)	Poland	*Leuconostoc mesenteroides, L. casei, L. plantarum, Pediococcus pentosaceus.*	4×10^{12} cfu/kg	ns	3.7	n/a
Rybarczyk et al. (2021)	Poland	*B. licheniformis and B. subtilis*	0.04	ADG, G:F	14.6	7.7
Shen et al. (2021)	China	*B. subtilis*	5×10^{9} cfu/kg	ns	0.8	6.7
		biodegradable *B. subtilis*	5×10^{9} cfu/kg	G:F	3.6	13.0
Tutida et al. (2021)	Brazil	*B. spp., B. bifidum, E. faecium, L. acidophilus*	0.05	ns	-1.4	0.0

[1]Significant level at $P \leq 0.05$. Difference is calculated as [(treatment value – control value) / control value] * 100%.

[2]Polynomial contrasts were used for statistical analysis.

[3]Significant level at $0.05 < P \leq 0.10$.

[4]*Bacillus spp.* is abbreviated as *B.*, and *Lactobacillus spp.* is abbreviated as *L.*

[5]The basal diets in the middle and bottom comparison contained long-chain inulin and Jerusalem artichoke, respectively, while the top comparison did not.

1.3.2. Carcass Characteristics - DFM

Back-fat significantly increased ($P \leq 0.05$) in 1 comparison (16.8%), significantly decreased ($P \leq 0.05$) in 2 comparisons (average of 13.1%) and tended to decrease ($0.05 < P \leq 0.10$) in 3 comparisons (average of 2.9%) compared to control pigs (Table S6). The greatest proportion of the comparisons found no evidence of difference ($P > 0.10$) in BF (15 comparisons). Of these, BF was numerically increased ($P > 0.10$) in 6 comparisons (average of 7.1%) and numerically decreased ($P > 0.10$) in 9 comparisons (average of 6.3%) compared to control pigs. Percentage lean tended to increase ($0.05 < P \leq 0.10$) in 1 comparison (1.8%) compared to control pigs. The greatest proportion of the comparisons found no evidence of difference ($P > 0.10$) in percentage lean (12 comparisons). Of these, percentage lean was numerically increased ($P > 0.10$) in 9 comparisons (average of 1.8%) and numerically decreased ($P > 0.10$) in 3 comparisons (average of 1.8%) compared to control pigs. Loin muscle area/depth significantly increased ($P \leq 0.05$) in 1 comparison (average of 10.9%) compared to control pigs. The greatest proportion of the comparisons found no evidence of difference ($P > 0.10$) in LMA/LD (18 comparisons). Of these, LMA/LD was numerically increased ($P > 0.10$) in 14 comparisons (average of 2%) and numerically decreased ($P > 0.10$) in 3 comparisons (average of 3.4%) compared to control pigs. The small effects and lack of statistical differences of DFM on carcass characteristics may suggest that the mechanisms of DFM do not directly affect pigs' protein and lipid metabolism.

Even though DFM has beneficial effects on growth performance, the improvement in growth did not equally improve BF, percentage lean, and LMA/LD to the same extent.

Table S6. Studies on the effects of DFM on carcass characteristics.

Author	Country	DFM[4]	Inclusion, %	Sig.[1]	Difference, %[1]			
					Yield	BF	percentage lean	LMA/LD
Kim et al. (1998)	USA	*L. acidophilus*	0.05	ns	0.8	-5.6	1.5	n/a
Jukna et al. (2005)	Lithuania	*Saccharamyce cerevisiae, L. casei, L. acidophilus, Streptococcus faecium, B. subtilis*	0.20	ns	2.7	n/a	n/a	n/a
Rekiel et al. (2005)	Poland	*Pediococcus acidilactici*	0.01	ns	0.3	0.4	2.4	2.2
Černauskienė et al. (2010), Exp. 1	Lithuania	*Enterococcus faecium*	10^{10} cfu/kg	ns	-0.3	n/a	-2.0	n/a
Černauskienė et al. (2010), Exp. 2	Lithuania	*Enterococcus faecium*	10^{10} cfu/kg	ns	0.1	n/a	n/a	n/a
Meng et al. (2010)	South Korea	*B. subtilis endospores and Clostridium butyricum*	0.2	ns	n/a	n/a	n/a	4.3
Nitikanchana et al. (2011)	USA	*B. species*	0.2×10^9 cfu/g	BF[2,3]	0.5	-4.2	0.7	0.0
			2×10^9 cfu/g		0.9	-1.4	0.5	1.1
Hossain et al. (2012)	South Korea	*L. acidophilus, L. plantarum, B. subtilis, B. coagulans, and Saccharomyces cerevisiae*	0.5	ns	n/a	20.3	n/a	n/a
Cui et al. (2013)	China	*B. subtilis*	2.0	BF, LMA	2.0	16.8	n/a	10.9
Balasubramanian et al. (2016)	South Korea	*B. coagulans, B. licheniformis, B. subtilis, and Clostridium butyricum*	0.01	ns[2]	n/a	4.5	0.9	1.2
			0.02		n/a	-5.1	1.5	2.4
Sarker et al. (2016)	South Korea	*L. acidophilus, L. plantarum, B. subtilis, B. coagulans, and Saccharomyces cerevisiae*	0.2	ns	n/a	-10.9	n/a	n/a
			0.4	ns	n/a	-4.2	n/a	n/a
			0.8	ns	n/a	-9.1	n/a	n/a
Nguyen et al. (2017)	South Korea	*Enterococcus faecium*	0.01	ns	n/a	n/a	n/a	0.5
Balasubramanian et al. (2018)	South Korea	*B. coagulans, B. licheniformis, B. subtilis, and Clostridium butyricum*	0.01	ns	n/a	-2.2	n/a	0.1
			0.02	BF	n/a	-8.2	n/a	2.8
Bučko et al. (2018)	Slovak	*L. plantarum*	3g/day	BF	n/a	-18.1	3.6	1.2
Nguyen Thi (2018)	Vietnam	*B. subtilis, L. spp., Saccharomyces cerevisiae*	0.2	ns	-0.8	-1.0	n/a	2.9
Runjun et al. (2018)	India	*P. acidilactici*	2×10^9 cfu/g	ns	0.6	-10.7	3.1	n/a
		L. acidophilus	2×10^9 cfu/g	ns	-0.5	-7.9	2.1	n/a
Reszka et al. (2020)	Poland	EM Carbon Bokash	0.5/0.3	ns	n/a	n/a	n/a	5.0

			0.5/0.3	ns	n/a	n/a	n/a	-1.5
			0.5/0.3	ns	n/a	n/a	n/a	0.6
Rybarczyk et al. (2020)	Poland	*Saccharomyces cerevisiae, L. casei, and L. plantarum*	0.30	ns	n/a	8.1	-2.0	-5.8
			0.50	ns	n/a	6.3	-1.3	-2.9
Grela et al. (2021)	Poland	*Lactococcus lactis, Carnobacterium divergens S1, L. casei, L. plantarum, and Sacharomyces cerevisiae*	0.1	BF[3], percentage lean[3]	-0.5	-3.0	1.8	1.9
Rybarczyk et al. (2021)	Poland	*B. licheniformis and B. subtilis*	0.04	ns	0.9	n/a	n/a	n/a
Tian et al. (2021)	China	*L. reuteri*	5×10^{10} cfu/kg	ns	2.2	3.1	n/a	1.9

[1]Significant level at $P \leq 0.05$. Difference is calculated as [(treatment value – control value) / control value] * 100%.
[2]Polynomial contrasts were used for statistical analysis.
[3]Significant level at $0.05 < P \leq 0.10$.
[4]*Bacillus spp.* is abbreviated as *B.*, and *Lactobacillus spp.* is abbreviated as *L.*

1.4. *Yeasts – Yeast Culture and Yeast-Derived Ingredients*

There were 22 research articles for yeasts with 36 comparisons from 12 countries during the grow-finish or finishing period which met the requirements for inclusion. Of these, 36 comparisons reported growth performance data, and 24 comparisons reported carcass data. Yeast was included in the diets as yeast culture, hydrolysate yeast culture, or mannan oligosaccharide (**MOS**). Yeast products were derived from Saccharomyces cerevisiae (yeast) and Phaffia rhodozyma (red yeast) yeast strains. Because of the lack of studies for individual yeast products, the effects of yeast culture and yeast-derived ingredients were combined and discussed for growth performance and carcass characteristics.

1.4.1. *Growth Performance - Yeasts*

Average daily gain significantly increased ($P \leq 0.05$) in 9 comparisons (average of 5.6%) compared to control pigs (Table S7). The greatest proportion of the comparisons found no evidence of difference ($P > 0.10$) in ADG (27 comparisons). Of these, ADG was numerically increased ($P > 0.10$) in 16 comparisons (average of 3.2%) and numerically decreased ($P > 0.10$) in 11 comparisons (average of 4.1%) compared to control pigs. Feed efficiency significantly increased ($P \leq 0.05$) in 10 comparisons (average of 7.8%) compared to control pigs. The greatest proportion of the comparisons found no evidence of difference ($P > 0.10$) in G:F (23 comparisons). Of these, G:F was numerically increased ($P > 0.10$) in 12 comparisons (average of 3.9%) and numerically decreased ($P > 0.10$) in 10 comparisons (average of 3.6%) compared to

control pigs. There were not enough data to support whether different basal diets affected the response of yeasts on ADG and G:F. Moreover, there were insufficient comparisons or information to determine the effect of different concentrations of active yeast ingredients on ADG and G:F. Overall, the results suggest that yeasts positively affected ADG and G:F (69 and 67% of all the comparisons, respectively), with 25 and 30% of all the comparisons being significant ($P \leq 0.05$). In summary, yeasts can be a potential feed additive with relatively large magnitude on improving the growth performance of grow-finish pigs, especially for G:F.

Table S7. Studies on the effects of yeasts and yeast-derived ingredients on growth performance.

Author	Country	Yeast form[6]	Inclusion, %	Sig.[1]	Difference, %[1]	
					ADG	G:F
Barber et al. (1971)	UK	Yeast culture	(7.1 and 3.1)/(3.6 and 1.6)[4]	ADG	2.9	n/a
Bowman and Veum (1973)	USA	Yeast culture	2.00[5]	ns	-5.3	0.0
			2.00[5]	ns	2.9	3.3
Burnett and Neil (1977), Exp. 1	UK	Yeast culture	0.05	ns	-0.6	-0.9
Burnett and Neil (1977), Exp. 2	UK	Yeast culture	0.05	ns	0.6	0.0
Bae et al. (1999)	South Korea	MOS	0.10	ns	1.3	4.7
Davis et al. (2002)	USA	MOS	0.2	ns	1.2	-1.3
Campbell et al. (2006)	Ireland	MOS	0.15	ns	-5.4	-7.0
Reynoso-González et al. (2010), Exp. 1	Mexico	Yeast culture	0.75	ns	-7.0	-2.6
			1.5	ns	-3.5	-3.2
Reynoso-González et al. (2010), Exp. 2	Mexico	Yeast culture	0.75[5]	ns	4.8	4.9
			0.75[5]	ns	-2.2	-6.1
Ha et al. (2012)	South Korea	Yeast culture	2.0	ns	-1.4	n/a
Kerr et al. (2013)	USA	Yeast culture	0.1	ns	-13.7	-11.7
Wenner et al. (2013)	USA	MOS	0.2/0.1/0.055	ns	0.8	1.6
Edwards et al. (2014)	Australia	MOS	0.04/0.025	ns	3.6	3.9
Lei and Kim (2014)	South Korea	Yeast culture[6]	0.1	G:F[2]	3.6	4.6
			0.2		2.4	5.2
Giannenas et al. (2016)	Greece	MOS	0.1	ns	3.4	9.1
Szakacs et al. (2016)	Romania	Yeast extract	0.03	ns	4.9	10.4
			0.03	ns	1.6	-2.5
Gong et al. (2018)	China	Yeast culture	0.3	ADG	8.4	5.2
Zhang et al. (2019)	South Korea	Hydrolysate yeast culture	0.05	ADG[2], G:F[2]	2.6	3.8
			0.10		3.7	0.8
			0.50		3.9	4.8
			1.00		7.0	5.0
Bo et al. (2020)	Vietnam	Yeast extract	2.00	G:F	6.0	13.2
			4.00	G:F	9.7	17.7
			6.00	G:F	10.3	16.8
Dávila-Ramírez et al. (2020)	Mexico	Yeast culture	0.2	ADG	5.4	2.0
			0.3	ADG	6.1	-0.5

He et al. (2021)	China	Yeast culture	2.0	ns	-1.6	1.5
Mayorga et al. (2021)	USA	Yeast culture	0.025	ns	3.3	n/a
Namted et al. (2021)	Thailand	Hydrolysate yeast culture	0.5	G:F	-1.6	6.6
			1.0	ns	-2.3	0.4
Tutida et al. (2021)	Brazil	MOS	0.04/0.02	ns	0.8	-0.4

[1]Significant level at $P \leq 0.05$. Difference is calculated as [(treatment value – control value) / control value] * 100%.

[2]Polynomial contrasts were used for statistical analysis.

[3]Significant level at $0.05 < P \leq 0.10$.

[4]Inclusion levels of sequential phases.

[5]For experiments using factorial treatment structures, if the interaction of factors of either interested variable was observed the effect of the feed additive within each level of the other factor is included within the database.

[6]Yeast culture and yeast-derived ingredients (MOS) were produced from strains of *Saccharomyces cerevisiae*, except the yeast culture used in Lei and Kim (2014). Yeast culture used in Lei and Kim (2014) was derived from *Phaffia rhodozyma*.

1.4.2. Carcass Characteristics - Yeasts

All 21 comparisons found no evidence of difference ($P > 0.10$) in BF. Of these, BF was numerically increased ($P > 0.10$) in 12 comparisons (average of 4.1%) and numerically decreased ($P > 0.10$) in 8 comparisons (average of 14.4%) compared to control pigs (Table S8). Percentage lean tended to increase ($0.05 < P \leq 0.10$) in 3 comparisons (average of 0.8%) and tended to decrease ($0.05 < P \leq 0.10$) in 1 comparison (1.2%) compared to control pigs. Half of the studies found no evidence of difference ($P > 0.10$) in percentage lean (4 comparisons). Of these, percentage lean was numerically increased ($P > 0.10$) in 2 comparisons (average of 4.9%) and numerically decreased ($P > 0.10$) in 2 comparisons (average of 1.3%) compared to control pigs.. All the comparisons found no evidence of difference ($P > 0.10$) in LMA/LD. Of these, LMA/LD was numerically increased ($P > 0.10$) in 10 comparisons (average of 3.6%) and numerically decreased ($P > 0.10$) in 7 comparisons (average of 1.9%) compared to control pigs.

Table S8. Studies on the effects of yeasts and yeast-derived ingredients on carcass characteristics.

Author	Country	Yeast form[6]	Inclusion, %	Sig.[1]	Difference, %[1]			
					Yield	BF	percentage lean	LMA/LD
Barber et al. (1971)	UK	Yeast culture	(7.1 and 3.1)/(3.6 and 1.6)[4]	ns	-0.9	0.5	n/a	-0.6
Bowman and Veum (1973)	USA	Yeast culture	2.00[5]	ns	n/a	7.1	-0.9	-3.2
			2.00[5]	ns	n/a	-2.8	-1.7	0.7
Burnett and Neil (1977), Exp. 2	UK	Yeast culture	0.05	ns	0.9	2.5	n/a	n/a
Campbell et al. (2006)	Ireland	MOS	0.15	ns	-0.6	n/a	n/a	n/a
Reynoso-González et al. (2010), Exp. 1	Mexico	Yeast culture	0.75	ns	-2.3	-12.4	n/a	-2.4
			1.5	ns	0.0	-19.4	n/a	6.9
Reynoso-González et al. (2010), Exp. 2	Mexico	Yeast culture	0.75[5]	ns	1.1	-10.6	n/a	0.9
			0.75[5]	ns	-0.8	8.0	n/a	-0.3
Ha et al. (2012)	South Korea	Yeast culture	2.0	ns	n/a	0.0	n/a	n/a
Wenner et al. (2013)	USA	MOS	0.2/0.1/0.055	ns	n/a	-2.6	n/a	1.4
Edwards et al. (2014)	Australia	MOS	0.04/0.025	Yield	2.2	2.7	n/a	n/a
Lei and Kim (2014)	South Korea	Yeast culture[6]	0.1	ns[2]	n/a	n/a	n/a	2.5
			0.2		n/a	n/a	n/a	0.8
Zhang et al. (2019)	South Korea	Hydrolysate yeast culture	0.05	percentage lean[2,3]	n/a	0.8	-1.2	n/a
			0.1		n/a	3.3	0.6	n/a
			0.5		n/a	3.5	1.4	n/a
			1.0		n/a	4.5	0.4	n/a
Bo et al. (2020)	Vietnam	Yeast extract	2.00	ns	n/a	0.0	n/a	1.6
			4.00	ns	n/a	1.3	n/a	0.5
			6.00	ns	n/a	4.0	n/a	-0.3
Dávila-Ramírez et al. (2020)	Mexico	Yeast culture	0.2	ns	0.0	-24.6	n/a	4.7
			0.3	ns	0.5	11.2	n/a	16.6
Namted et al. (2021)	Thailand	Hydrolysate yeast culture	0.5	ns	-0.8	-30.7	6.6	-1.9
			1.0	ns	-0.4	-12.2	3.2	-4.3

[1]Significant level at $P \leq 0.05$. Difference is calculated as [(treatment value – control value) / control value] * 100%.

[2]Polynomial contrasts were used for statistical analysis.

[3]Significant level at $0.05 < P \leq 0.10$.

[4]Inclusion levels of sequential phases.

[5]For experiments using factorial treatment structures, if the interaction of factors of either interested variable was observed the effect of the feed additive within each level of the other factor is included within the database.

[6]Yeast culture and yeast-derived ingredients (MOS) were produced from strains of *Saccharomyces cerevisiae*, except the yeast culture used in Lei and Kim (2014). Yeast culture used in Lei and Kim (2014) was derived from *Phaffia rhodozyma*.

1.5. Copper (Cu)

There were 55 research articles for Cu with 157 comparisons from 11 countries during the grow-finish or finishing period with added dietary levels of 50 to 300 mg/kg with most studies ranged between 120 to 250 mg/kg. Of these, 155 comparisons reported growth performance data and 83 comparisons reported carcass data. The Cu sources used in the studies were in inorganic [$CuSO_4$, Cu_2O, CuO, Tribasic Cu chloride (**TBCC**), CuS] or organic form (Cu-AAs).

1.5.1. Growth Performance - Cu

Average daily gain significantly increased ($P \leq 0.05$) in 30 comparisons (average of 6.2%), tended to increase ($0.05 < P \leq 0.10$) in 3 comparisons (average of 4.1%), and significantly decreased ($P \leq 0.05$) in 1 comparison (0.1%) compared to control pig (Table S9). The greatest proportion of the comparisons found no evidence of difference ($P > 0.10$) in ADG (121 comparisons). Of these, ADG was numerically increased ($P > 0.10$) in 81 comparisons (average of 3.8%) and numerically decreased ($P > 0.10$) in 33 comparisons (average of 3.4%) compared to control pigs. Feed efficiency significantly increased ($P \leq 0.05$) in 30 comparisons (average of 5.1%), tended to increase ($0.05 < P \leq 0.10$) in 3 comparisons (average of 1.0%), and significantly decreased ($P \leq 0.05$) in 2 comparisons (average of 3.7%) compared to control pigs. The greatest proportion of the comparisons found no evidence of difference ($P > 0.10$) in G:F (114 comparisons). Of these, G:F was numerically increased ($P > 0.10$) in 71 comparisons (average of 3.1%) and numerically decreased ($P > 0.10$) in 37 comparisons (average of 2.7%) compared to

control pigs. Overall, the results suggest that Cu positively affected ADG and G:F (74 and 70 % of all the comparisons). Most studies used corn or barley as the major ingredient in basal diets. Copper supplementation in the barley diet had a greater percentage improvement in ADG (2.8%; 60 comparisons) and G:F (3.1%; 59 comparisons) than Cu supplementation in corn-based diets [ADG (2.0%; 89 comparisons) and G:F (0.8%; 84 comparisons)]. Most studies used Cu inclusion from 125 to 250 mg/kg added level (137 comparisons) and increasing the Cu level did not further improve the performance. In summary, the growth-promoting effects of Cu can potentially improve growth performance (2.5 and 1.8% improvement for ADG and G:F).

Table S9. Studies on the effects of Cu on growth performance.

Author	Country	Cu	Inclusion, mg/kg	Sig.	Difference, %	
					ADG	G:F
Lucas and Calder (1957), Exp. 1	UK	$CuSO_4$	200	ns	3.9	0.7
Lucas and Calder (1957), Exp. 2	UK	$CuSO_4$	200	ns	6.5	7.1
King (1960)	UK	$CuSO_4$	0.10%	G:F	2.9	6.1
			0.10%	ADG, G:F	7.7	11.0
Wallace et al. (1960), Exp. 1	USA	$CuSO_4$	100	ns	11.1	-1.8
			150	ns	5.2	0.9
			200	ns	0.7	0.9
Wallace et al. (1960), Exp. 2	USA	$CuSO_4$	200[4]	ns	-3.8	-8.0
			200[4]	ns	3.3	4.5
Wallace et al. (1960), Exp. 3	USA	$CuSO_4$	100[4]	ns	0.0	17.6
			100[4]	ns	-6.5	0.3
Bellis (1961)	UK	$CuSO_4$	125	ns	3.9	3.3
			250	ADG, G:F	7.1	8.0
Lucas et al. (1961), Exp. 1	UK	$CuSO_4$	62	ns	-1.1	-1.4
			125	ns	1.1	0.6
			250	ns	4.5	0.6
Lucas et al. (1961), Exp. 2	UK	$CuSO_4$	62	ns	2.2	0.9
			125	ns	2.2	1.8
			250	ns	3.3	2.1
Barber et al. (1962)	UK	$CuSO_4$	250[4]	ADG, G:F	14.5	5.5
			250[4]	ADG	5.1	2.2
Braude et al. (1962)	UK	$CuSO_4$	250[4]	ADG, G:F	9.7	8.6
			250[4]	ADG, G:F	6.2	5.2
Lucas et al. (1962)	UK	$CuSO_4$	250	ns	2.6	-0.1
Gipp et al. (1967)	USA	CuO	150[4]	ns	-4.7	-2.3
			150[4]	ns	0.4	4.2
Barber et al. (1968), Exp. 1	UK	$CuSO_4$	250	ADG, G:F	11.1	9.0

Barber et al. (1968), Exp. 2	UK	$CuSO_4$	250	ns	7.1	3.9
Barber et al. (1968), Exp. 3	UK	$CuSO_4$	250	G:F	5.3	4.4
Hanrahan and O'Grady (1968)	Ireland	$CuSO_4$	250	ns	-12.2	-6.0
Boyazoglu and Barrett (1970)	South Africa	$CuSO_4$	150	ns	n/a	4.4
			300	ns	n/a	-0.8
Barber et al. (1971), Exp. 1	UK	$CuSO_4$	250[4]	G:F	4.5	5.3
			250[4]	G:F	-1.4	4.1
Barber et al. (1971), Exp. 2	UK	$CuSO_4$	250[4]	ADG, G:F	8.1	6.1
			250[4]	ns	-3.8	-1.3
Barber et al. (1971), Exp. 3	UK	$CuSO_4$	250	ADG, G:F	7.4	5.6
DeGoey et al. (1971)	USA	$CuSO_4$	250	ADG	15.2	1.9
Kline et al. (1971)	USA	$CuSO_4$	150	ADG[2]	7.2	-2.4
			200		-0.1	-7.8
			250		8.4	-0.5
Kline et al. (1972)	USA	$CuSO_4$	250[4]	ns	14.8	5.4
			250[4]	ns	-3.2	10.7
			250[4]	ns	5.5	-6.7
Braude and Ryder (1973)	UK	$CuSO_4$	150	ADG[2], G:F[2]	3.1	3.6
			200		4.4	3.9
			250		5.9	5.5
Elliot and Amer (1973), Exp. 1	Canada	$CuSO_4$	250	ns	-6.8	n/a
Elliot and Amer (1973), Exp. 2	Canada	$CuSO_4$	125	ns	-4.3	10.0
			150	ns	0.0	0.3
			175	ns	1.8	1.9
			200	ns	1.8	10.0
			225	ns	-3.7	1.9
			250	ns	-10.2	1.3
Gipp et al. (1973), Exp. 1	USA	$CuSO_4$	250	ns	4.1	3.3
Gipp et al. (1973), Exp. 2	USA	$CuSO_4$	250	ns	-1.3	-3.3
Gipp et al. (1973), Exp. 3	USA	$CuSO_4$	250	ns	-4.0	-0.7
Kline et al. (1973), Exp. 1	USA	$CuSO_4$	250	ADG, G:F	8.9	6.7
Kline et al. (1973), Exp. 2	USA	$CuSO_4$	250	ns	0.4	-2.9
Kline et al. (1973), Exp. 3	USA	$CuSO_4$	250	ns	-5.5	9.3
NCR-42 Committee on Swine Nutrition (1974), Exp. 1	USA	$CuSO_4$	250	ns	1.8	0.1
NCR-42 Committee on Swine Nutrition (1974), Exp. 2	USA	$CuSO_4$	125.5	ns	2.1	0.3
			187.5	ns	2.3	-0.3
			250	ns	3.5	1.5
Bellis (1975)	UK	$CuSO_4$	175[4]	ns	-1.5	0.0
			175[4]	ADG, G:F	3.1	2.8
Castell et al. (1975), Exp. 1	Canada	$CuSO_4$	125[5]	ns	4.8	-1.5
			200[5]	ns	8.7	4.1
			125[5]	ns	4.3	4.2
			200[5]	ns	6.3	3.5
Castell et al. (1975), Exp. 2	Canada	$CuSO_4$	125[5]	ns	2.0	2.2
			200[5]	ns	1.7	0.9
			125[5]	ns	2.8	5.5
			200[5]	ns	2.6	5.2
Castell et al. (1975), Exp. 3	Canada	$CuSO_4$	125	ns	-0.4	-3.3
			200	ns	0.9	-3.0
Castell et al. (1975), Exp. 4	Canada	$CuSO_4$	125	ns	2.0	3.3
			200	ns	-2.0	2.9
Castell et al. (1975), Exp. 5	Canada	$CuSO_4$	125	ns	3.3	2.1
			200	G:F	3.1	4.3

Hansen and Bresson (1975)	Denmark	$CuSO_4$	125	ADG, G:F	4.9	5.2
			200	ADG	3.9	3.2
Omole et al. (1976)	Nigeria	$CuSO_4$	125	ns	9.3	5.5
			200	G:F	14.8	8.5
Barber et al. (1978)	UK	NA	250	ADG, G:F	2.0	2.9
Cromwell et al. (1978), Exp. 1	USA	$CuSO_4$	125	ns	4.5	0.3
			188	ns	2.8	-2.2
			250	ns	14.0	2.0
Cromwell et al. (1978), Exp. 2	USA	$CuSO_4$	125	G:F	0.4	2.3
			250	G:F	1.3	3.5
Cromwell et al. (1978), Exp. 3	USA	$CuSO_4$	250	ns	2.8	2.5
		CuS	250	ns	-2.7	2.2
Pond et al. (1978)	USA	$CuSO_4$	200	ns	0.0	-3.2
Eisemann et al. (1979)	USA	$CuSO_4$	120	ns	-6.6	-2.0
Prince et al. (1979), Exp. 1	USA	$CuSO_4$	250	ns	1.8	1.5
Prince et al. (1979), Exp. 2	USA	$CuSO_4$	250	ns	5.3	5.4
Barber et al. (1981), Exp. 1	UK	$CuSO_4$	250	ADG, G:F	4.1	3.7
Barber et al. (1981), Exp. 2	UK	$CuSO_4$	250	ADG, G:F	2.6	2.5
Ribeiro de Lima et al. (1981), Exp. 1	USA	$CuSO_4$	250[4]	ns	10.0	1.7
			250[4]	ns	-4.6	0.0
			250[4]	ns	-3.7	-1.4
Ribeiro de Lima et al. (1981), Exp. 2	USA	$CuSO_4$	250	ns	0.8	-4.6
Ribeiro de Lima et al. (1981), Exp. 3	USA	$CuSO_4$	250	ns	0.0	1.5
Braude and Hosking (1982)	UK	$CuSO_4$	125	ADG, G:F	4.4	4.8
			200	ADG, G:F	3.1	2.8
			200/125	ADG, G:F	3.3	3.4
			250/125	ADG, G:F	4.9	4.8
Bradley et al. (1983)	USA	$CuSO_4$	52.5	ns	0.0	n/a
			112.5	ns	-1.8	n/a
			232.5	ns	-1.8	n/a
Prince et al. (1984), Exp. 1	USA	$CuSO_4$	250	ns	0.1	1.4
Prince et al. (1984), Exp. 2	USA	$CuSO_4$	250	ADG[3]	3.0	1.6
Southern and Stewart (1984), Exp. 1	USA	$CuSO_4$	250	ns	3.8	n/a
Southern and Stewart (1984), Exp. 2	USA	$CuSO_4$	250	ns	0.0	n/a
Rowan and Lawrence (1986)	UK	NA	183	ns	-0.1	-0.7
Astrup and Matre (1987)	Norway	$CuSO_4$	63	ns	1.1	6.3
			125	ns	4.8	5.3
			250	ns	3.5	4.6
Lüdke and Schöne (1988), Exp.1	Germany	$CuSO_4$	250	ns	10.7	2.1
Lüdke and Schöne (1988), Exp.2	Germany	$CuSO_4$	250	ns	5.7	2.5
Schöne et al. (1988)	Germany	$CuSO_4$	250	ns	14.2	5.2
Ward et al. (1991)	USA	$CuSO_4$	250	ns	2.5	-5.5
Myer et al. (1992)	USA	$CuSO_4$	250	ns	-1.1	-1.1
Southern et al. (1993)	USA	NA	250	ns	-1.1	0.0
Apgar and Kornegay (1996)	USA	$CuSO_4$	200	ns	-2.8	n/a

		Cu-Lys	200	ns	11.1	n/a
Lauridsen et al. (1999)	Denmark	$CuSO_4$	175	ns	0.3	-5.0
Davis et al. (2002)	USA	$CuSO_4$	175/125	ADG, G:F	6.6	3.6
Hernández et al. (2009)	Australia	Cu-AA	50[4]	G:F	-1.1	-5.4
			50[4]	ns	-3.2	0.4
Coble et al. (2014)	USA	$CuSO_4$	50	G:F	2.0	-1.9
			125	ns	1.5	-1.9
		Cu-AA	50	ns	2.0	-0.4
Feldpausch et al. (2016)	USA	$CuSO_4$	125[4]	ns	0.5	1.3
			125[4]	ns	-0.5	-1.3
Coble et al. (2017)	USA	$CuSO_4$/TBCC	75	ADG[2]	3.9	-2.2
			150		3.9	-1.8
Coble et al. (2018)	USA	TBCC	150	ns	1.7	0.3
Coble et al. (2018), Exp. 1	USA	TBCC	150[4]	ADG[3], GF[3]	0.0	0.3
			150[4]	ADG[3], GF[3]	0.0	1.1
			150[4]	ADG[3], GF[3]	2.4	1.6
Coble et al. (2018), Exp. 2	USA	TBCC	150[6]	ns	0.0	0.3
			150[6]	ns	1.1	0.0
			150[6]	ns	2.2	1.8
			150[6]	ns	1.1	0.6
Carpenter et al. (2019)	USA	$CuSO_4$/Cu-AA	70	ns[2]	1.7	0.8
			100		2.3	1.1
			130		1.1	1.7
Seidu et al. (2020)	China	$CuSO_4$	125	ns	4.2	-1.1
			215	ADG	5.2	-6.2
Blavi et al. (2021)	USA	$CuSO_4$	125	ns	2.2	0.0
			250	ns	1.1	-0.5
		Cu_2O	125	ns	2.2	0.0
			250	ADG[3]	6.7	-2.9

[1]Significant level at $P \le 0.05$. Difference is calculated as [(treatment value – control value) / control value] * 100%.

[2]Polynomial contrasts were used for statistical analysis.

[3]Significant level at $0.05 < P \le 0.10$.

[4]For experiments using factorial treatment structures, if the interaction of factors of either interested variable was observed the effect of the feed additive within each level of the other factor is included within the database.

[5]The top two comparisons were the results of the barrows and the bottom two comparisons were the results of the gilts.

[6]The top two comparisons were the results of the feeding Cu in grow-finish phase and the bottom two comparisons were the results of feeding Cu in the finish phase. The basal diet Lys concentrations from the top to bottom comparisons were at 92.5, 100, 92.5, and 100% of the requirement.

1.5.2. Carcass Characteristics – Cu

Back-fat significantly decreased ($P \le 0.05$) in 3 comparisons (average of 10.3%) and tended to decrease ($0.05 < P \le 0.10$) in 1 comparison (5.4%) compared to control pigs (Table S10). The

greatest proportion of the comparisons found no evidence of difference ($P > 0.10$) in BF (69 comparisons). Of these, BF was numerically increased ($P > 0.10$) in 24 comparisons (average of 3.5%) and numerically decreased ($P > 0.10$) in 36 comparisons (average of 4.1%) compared to control pigs. Percentage lean significantly increased ($P \leq 0.05$) in 2 comparisons (average of 1.1%) compared to control pigs. The greatest proportion of the comparisons found no evidence of difference ($P > 0.10$) in percentage lean (23 comparisons). Of these, percentage lean was numerically increased ($P > 0.10$) in 16 comparisons (average of 2.8%) and numerically decreased ($P > 0.10$) in 7 comparisons (average of 1.1%) compared to control pigs. Loin muscle area/depth significantly increased ($P \leq 0.05$) in 5 comparisons (average of 4.4%) and significantly decreased ($P \leq 0.05$) in 1 comparison (7.5%) compared to control pigs. The greatest proportion of the comparisons found no evidence of difference ($P > 0.10$) in LMA/LD (56 comparisons). Of these, LMA/LD was numerically increased ($P > 0.10$) in 43 comparisons (average of 3.4%) and numerically decreased ($P > 0.10$) in 11 comparisons (average of 1.4%) compared to control pigs.

Table S10. Studies on the effects of Cu on carcass characteristics.

Author	Country	Form	Inclusion, mg/kg	Sig.	Difference, %			
					Yield	BF	percentage lean	LMA/LD
Lucas and Calder (1957), Exp. 1	UK	$CuSO_4$	200	ns	1.3	-7.5	n/a	1.5
Lucas and Calder (1957), Exp. 2	UK	$CuSO_4$	200	ns	-0.1	-9.1	n/a	2.7
Bellis (1961)	UK	$CuSO_4$	125	ns	0.5	n/a	n/a	n/a
			250	ns	0.1	n/a	n/a	n/a
Lucas et al. (1961), Exp. 1	UK	$CuSO_4$	62	ns	1.1	-4.8	n/a	1.4
			125	ns	1.8	0.0	n/a	-0.7
			250	ns	2.0	0.0	n/a	-1.4
Lucas et al. (1961), Exp. 2	UK	$CuSO_4$	62	ns	-0.3	0.0	n/a	5.4
			125	ns	0.4	3.8	n/a	2.5
			250	ns	0.7	3.8	n/a	5.0
Braude et al. (1962)	UK	$CuSO_4$	250[4]	ns	n/a	0.7	n/a	1.4
			250[4]	ns	n/a	1.4	n/a	3.9
Lucas et al. (1962)	UK	$CuSO_4$	250	ns	-0.1	4.4	n/a	5.0
Boyazoglu and Barrett (1970)	South Africa	$CuSO_4$	150	ns	0.0	-7.5	n/a	12.5
			300	ns	0.6	-10.0	n/a	9.5
Barber et al. (1971), Exp. 1	UK	$CuSO_4$	250[4]	ns	1.1	-1.2	n/a	n/a
			250[4]	ns	0.4	-17.0	n/a	n/a
Barber et al. (1971), Exp. 2	UK	$CuSO_4$	250[4]	ns	0.1	-4.0	n/a	n/a
			250[4]	ns	0.3	9.3	n/a	n/a
Barber et al. (1971), Exp. 3	UK	$CuSO_4$	250	ns	-0.5	-1.0	n/a	n/a
Braude and Ryder (1973)	UK	$CuSO_4$	150		0.5	1.9	n/a	1.9
			200	ns[2]	0.7	0.0	n/a	1.9
			250		0.4	0.0	n/a	3.4
Gipp et al. (1973), Exp. 2	USA	$CuSO_4$	250	LMA	n/a	-2.4	-1.3	-7.5
Gipp et al. (1973), Exp. 3	USA	$CuSO_4$	250	ns	n/a	-1.5	0.7	2.1
NCR-42 Committee on Swine Nutrition (1974), Exp. 1	USA	$CuSO_4$	250	ns	n/a	-10.2	n/a	4.3
Bellis (1975)	UK	$CuSO_4$	175	ns	0.1	-2.7	n/a	1.0
			175	ns	0.4	2.2	n/a	1.7
Castell et al. (1975), Exp. 1	Canada	$CuSO_4$	125[5]	ns	1.9	n/a	n/a	0.0
			200[5]	ns	-0.1	n/a	n/a	-2.3
			125[5]	ns	-1.4	n/a	n/a	1.7
			200[5]	ns	1.1	n/a	n/a	5.6

Castell et al. (1975), Exp. 2	Canada	$CuSO_4$	125[5]	ns	0.1	n/a	n/a	7.3
			200[5]	ns	-1.9	n/a	n/a	4.8
			125[5]	ns	-1.9	n/a	n/a	-1.3
			200[5]	ns	0.2	n/a	n/a	2.9
Castell et al. (1975), Exp. 3	Canada	$CuSO_4$	125	ns	0.5	1.7	n/a	3.7
			200	ns	0.5	3.3	n/a	3.0
Castell et al. (1975), Exp. 4	Canada	$CuSO_4$	125	ns	1.0	-1.9	n/a	4.1
			200	ns	0.8	-4.6	n/a	12.0
Castell et al. (1975), Exp. 5	Canada	$CuSO_4$	125	ns	0.4	-1.0	n/a	3.4
			200	ns	0.5	-1.9	n/a	2.7
Hansen and Bresson (1975)	Denmark	$CuSO_4$	125	ns	n/a	0.0	n/a	n/a
			200	ns	n/a	2.9	n/a	n/a
Omole et al. (1976)	Nigeria	$CuSO_4$	125	ns	-0.3	2.6	n/a	3.4
			200	ns	0.8	-5.8	n/a	14.5
Barber et al. (1978)	UK	n/a	250	BF	-0.3	-8.1	n/a	n/a
Barber et al. (1981), Exp. 1	UK	$CuSO_4$	250	BF	-0.8	-21.0	n/a	0.6
Barber et al. (1981), Exp. 2	UK	$CuSO_4$	250	ns	0.0	0.0	n/a	n/a
Braude and Hosking (1982)	UK	$CuSO_4$	125	ns	0.4	-3.2	n/a	n/a
			200	ns	0.7	-1.4	n/a	n/a
			200/125	ns	1.1	-2.7	n/a	n/a
			250/125	ns	0.4	-2.7	n/a	n/a
Rowan and Lawrence (1986)	UK	NA	183	ns	-0.4	0.0	n/a	n/a
Astrup and Matre (1987)	Norway	$CuSO_4$	63	ns	1.1	3.1	n/a	n/a
			125	ns	0.7	2.7	n/a	n/a
			250	ns	0.0	-1.0	n/a	n/a
Ward et al. (1991)	USA	$CuSO_4$	250	ns	-0.7	-5.4	1.4	-0.9
Myer et al. (1992)	USA	$CuSO_4$	250	ns	n/a	2.9	n/a	-3.1
Southern et al. (1993)	USA	NA	250	ns	-0.7	n/a	n/a	n/a
Hernández et al. (2009)	Australia	Cu-AA	50[4]	ns	-0.3	-3.4	n/a	n/a
			50[4]	ns	-0.6	-13.6	n/a	n/a
Coble et al. (2014)	USA	$CuSO_4$	50	ns	-0.8	3.1	-0.4	0.0
			125	ns	-0.1	-1.6	0.1	-0.4
		Cu-AA	50	ns	-0.6	3.1	34.7	1.1
Feldpausch et al. (2016)	USA	$CuSO_4$	125[4]	ns	0.2	-0.3	0.4	0.6
			125[4]	ns	-0.1	0.8	0.4	0.9
Coble et al. (2017)	USA		75		-0.8	-5.8	1.4	2.2

		$CuSO_4$/TBCC	150	percentage lean[2], LD[2]	-0.5	-2.6	0.8	2.1
Coble et al. (2018)	USA	TBCC	150	ns	0.2	0.0	0.0	0.6
Coble et al. (2018), Exp. 1	USA	TBCC	150[4]	ns	0.0	-1.8	0.3	-1.1
			150[4]	ns	-0.5	-3.0	1.3	1.2
			150[4]	ns	0.3	1.9	-0.4	2.6
Coble et al. (2018), Exp. 2	USA	TBCC	150[6]	ns	2.2	-6.0	1.3	0.9
			150[6]	ns	-0.2	7.1	-1.2	-1.2
			150[6]	ns	0.9	-6.5	1.3	0.9
			150[6]	ns	-0.4	7.7	-2.7	-2.6
Carpenter et al. (2019)	USA	$CuSO_4$/Cu-AA	70	ns[2]	0.4	0.9	-0.2	0.2
			100		0.4	-0.6	0.1	-0.5
			130		-0.3	-0.3	0.2	0.9
Blavi et al. (2021)	USA	$CuSO_4$	125	ns	0.3	11.5	-1.2	5.5
			250	ns	0.4	-1.8	0.5	3.6
		Cu_2O	125	ns	0.5	1.8	0.1	1.5
			250	ns	0.5	-9.7	1.3	6.6

[1]Significant level at $P \leq 0.05$. Difference is calculated as [(treatment value – control value) / control value] * 100%.

[2]Polynomial contrasts were used for statistical analysis.

[3]Significant level at $0.05 < P \leq 0.10$.

[4]For experiments using factorial treatment structures, if the interaction of factors of either interested variable was observed the effect of the feed additive within each level of the other factor is included within the database.

[5]The top two comparisons were the results of the barrows and the bottom two comparisons were the results of the gilts.

[6]The top two comparisons were the results of the feeding Cu in grow-finish phase and the bottom two comparisons were the results of feeding Cu in the finish phase. The basal diet Lys concentrations from the top to bottom comparisons were at 92.5, 100, 92.5, and 100% of the requirement.

1.6. Zinc (Zn)

There were 13 research articles for Zn with 30 comparisons from 6 countries during the grow-finish or finishing period which met the requirements for inclusion. Of these, 30 comparisons reported growth performance data, and 21 comparisons reported carcass data. The growth-promotive levels of Zn were close to the control Zn levels used in most research (ranged approximately between 50 to 100 mg/kg); therefore, only trials with the total Zn level above or at approximately 100 mg/kg were used in this literature review. The difference in Zn levels between control diets and the growth-promotive Zn diets ranged between 38 to 400 mg/kg. The Zn sources used in the studies were inorganic (ZnO, $ZnSO_4$, Zn-HCl, Zn hydroxy chloride) or organic form (Zinc glycinate, Zn-AA).

1.6.1. Growth Performance – Zn

Average daily gain significantly increased ($P \leq 0.05$) in 1 comparison (18.7%), tended to increase ($0.05 < P \leq 0.10$) in 1 comparison (1.1%), and significantly decreased ($P \leq 0.05$) in 1 comparison (14.4%) compared to control pigs (Table S11). The greatest proportion of the comparisons found no evidence of difference ($P > 0.10$) in ADG (27 comparisons). Of these, ADG was numerically increased ($P > 0.10$) in 12 comparisons (average of 4.0%) and numerically decreased ($P > 0.10$) in 11 comparisons (average of 3.2%) compared to control pigs. Feed efficiency tended to increase ($0.05 < P \leq 0.10$) in 4 comparisons (average of 1.2%) compared to control pigs. The greatest proportion of the comparisons found no evidence of

difference ($P > 0.10$) in ADG (26 comparisons). Of these, G:F was numerically increased ($P > 0.10$) in 14 comparisons (average of 4.2%) and numerically decreased ($P > 0.10$) in 11 comparisons (average of 2.6%) compared to control pigs. Overall, the results suggest that Zn had positive but relatively small effects on ADG and G:F. Moreover, there were insufficient data to support whether different types of basal diets and inclusion levels affected the response to Zn for ADG and G:F.

Table S11. Studies on the effects of Zn on growth performance.

Author	Country	Zn	Basal, mg/kg	Added, mg/kg	Sig.[1]	Difference, %[1]	
						ADG	G:F
Kline et al. (1972)	USA	$ZnSO_4$	100	100[4]	ns	8.1	-5.1
			100	200[4]	ns	-1.8	14.4
			100	100[4]	ns	-8.8	-0.3
			100	200[4]	ns	-9.7	1.3
			100	100[4]	ns	0.0	-3.7
			100	200[4]	ns	5.7	-2.5
Omole et al. (1976)	Nigeria	Zn powder	50	100[4]	ns	11.9	9.0
			50	100[4]	ns	9.7	5.0
Eisemann et al. (1979)	USA	ZnO	100	400	ns	2.7	4.4
Wedekind et al. (1994)	USA	ZnSO4	52	60	ns	-3.4	0.0
Rupić et al. (1997)	Croatia	ZnSO4	37	84	ADG	18.7	3.8
Hernández et al. (2009)	Australia	Zn-AA	70	40[4]	ns	0.2	-1.5
			70	40[4]	ns	1.8	1.5
			70	40[4]	ns	2.7	2.2
			70	40[4]	ns	-3.0	3.7
Paulk et al. (2014)	USA	ZnO	50	75[5]	ns	0.0	-2.9
			50	75[5]	ns	-1.8	-2.1
Feldpausch et al. (2016)	USA	ZnO	110	150[4]	ns	1.0	1.3
			110	150[4]	ns	0.0	-1.3
Holen et al. (2018)	USA	Zn-AA	70	40	ns	2.2	3.7
			70	80	ns	1.1	3.4
		$ZnSO_4$	70	80	ns	2.2	2.5
Cemin et al. (2019)	USA	Zn hydroxychloride/ $ZnSO_4$	113	50	ADG[2,3]	1.1	-0.3
			113	50		0.0	-0.8
Cemin et al. (2019)	USA	Zn hydroxychloride	50	37.5	G:F[2,3]	-2.1	0.5
			50	75		-1.1	1.9
			50	112.5		-1.1	1.4
			50	150		-1.1	0.8
Villagómez-Estrada et al. (2021)	Spain	$ZnSO_4$/Zn-HCl	60	60	ns	-1.4	1.4
Natalello et al. (2022)	Italy	Zn glycinate	22.3	100	ADG	-14.4	-7.6

[1]Significant level at $P \leq 0.05$. Difference is calculated as [(treatment value – control value) / control value] * 100%.

[2]Polynomial contrasts were used for statistical analysis.

[3]Significant level at $0.05 < P \leq 0.10$.

[4]For experiments using factorial treatment structures, if the interaction of factors of either interested variable was observed the effect of the feed additive within each level of the other factor is included within the database.

[5]High Zn diet was fed for 72 d in the top comparison and 27 d in the below comparison.

1.6.2. Carcass Characteristics - Zn

Back-fat significantly increased (*P* ≤ 0.05) in 1 comparison (13.1%) compared to control pigs. The greatest proportion of the comparisons found no evidence of difference (*P* > 0.10) in BF (18 comparisons; Table S12). Of these, BF was numerically increased (*P* > 0.10) in 5 comparisons (average of 1.3%) and numerically decreased (*P* > 0.10) in 11 comparisons (average of 2.9%) compared to control pigs. All the comparisons found no evidence of difference (*P* > 0.10) in ADG (14 comparisons). Of these, percentage lean was numerically increased (*P* > 0.10) in 12 comparisons (average of 1.1%) and numerically decreased (*P* > 0.10) in 1 comparison (0.4%) compared to control pigs. All the comparisons found no evidence of difference (*P* > 0.10) in LMA/LD (15 comparisons). Of these, LMA/LD was numerically increased (*P* > 0.10) in 11 comparisons (average of 0.9%) and numerically decreased (*P* > 0.10) in 4 comparisons (average of 1.5%) compared to control pigs.

Table S12. Studies on the effects of Zn on carcass characteristics.

Author	Country	Zn	Basal, mg/kg	Added, mg/kg	Sig.[1]	Difference, %[1]			
						Yield	BF	percent age lean	LMA/LD
Omole et al. (1976)	Nigeria	Zn powder	50	100[4]	ns	-0.5	-5.1	n/a	2.6
			50	100[4]	ns	-0.2	0.9	n/a	-2.9
Hernández et al. (2009)	Australia	Zn-AA	70	40[4]	ns	0.0	3.4	n/a	n/a
			70	40[4]	ns	0.9	-6.0	n/a	n/a
			70	40[4]	ns	-0.3	13.1	n/a	n/a
			70	40[4]	ns	-0.3	-7.6	n/a	n/a
Paulk et al. (2014)	USA	ZnO	50	75[5]	ns	-0.1	-1.7	3.9	0.4
			50	75[5]	ns	1.2	0.8	3.2	-1.8
Feldpausch et al. (2016)	USA	ZnO	110	150[4]	ns	0.1	-1.6	0.1	0.1
			110	150[4]	ns	-0.3	-0.5	0.1	0.4
Holen et al. (2018)	USA	Zn-AA	70	40	ns	-0.4	0.9	0.2	0.8
			70	80	ns	0.3	-3.2	0.3	-0.4
		$ZnSO_4$	70	80	ns	0.5	-2.3	1.3	2.7
Cemin et al. (2019)	USA	Zn hydroxychloride/ $ZnSO_4$	113	50	Yield[2]	1.4	0.0	1.4	0.5
			113	100		1.4	0.0	1.5	0.7
Cemin et al. (2019)	USA	Zn hydroxychloride	50	37.5	ns[2]	-0.3	0.0	0.2	0.6
			50	75		-0.5	-1.7	0.2	-0.9
			50	112.5		-0.1	0.6	0.0	0.7
			50	150		-0.1	-1.7	0.4	0.1
Villagómez-Estrada et al. (2021)	Spain	$ZnSO_4$/Zn-HCl	60	60	ns	0.3	n/a	-0.4	n/a
Natalello et al. (2022)	Italy	Zn glycinate	22.3	100	ns	-0.2	n/a	n/a	n/a

[1]Significant level at $P \leq 0.05$. Difference is calculated as [(treatment value – control value) / control value] * 100%.

[2]Polynomial contrasts were used for statistical analysis.

[3]Significant level at $0.05 < P \leq 0.10$.

[4]For experiments using factorial treatment structures, if the interaction of factors of either interested variable was observed the effect of the feed additive within each level of the other factor is included within the database.

[5]High Zn diet was fed for 72 d in the top comparison and 27 d in the below comparison.

2. Feed Additives – Energy and Lipid Metabolism

This section discusses the feed additives that can potentially improve growth performance and carcass characteristics by affecting the energy and lipid metabolism of grow-finish pigs. The feed additives discussed are betaine, Cr, CLA, and L-carnitine.

2.1. Betaine

There were 20 research articles for betaine with 37 comparisons from 9 countries during the grow-finish or finishing period with added dietary levels of 0.02 to 1.05 %. Of these, all comparisons reported growth performance data, and 32 comparisons reported carcass data.

2.1.1. Growth Performance - Betaine

Average daily gain significantly increased ($P \leq 0.05$) in 7 comparisons (average of 10.6%), tended to increase ($0.05 < P \leq 0.10$) in 1 comparison (4.3%), and significantly decreased ($P \leq 0.05$) in 2 comparisons (average of 2.8%) compared to control pigs (Table S13). The greatest proportion of the comparisons found no evidence of difference ($P > 0.10$) in ADG (27 comparisons). Of these, ADG was numerically increased ($P > 0.10$) in 10 comparisons (average of 2.4%) and numerically decreased in 15 comparisons (average of 3.3%) compared to control pigs. Feed efficiency significantly increased ($P \leq 0.05$) in 5 comparisons (average of 13.2%) and significantly decreased ($P \leq 0.05$) in 1 comparison (0.4%) compared to control pigs. The greatest proportion of the comparisons found no evidence of difference ($P > 0.10$) in G:F (29 comparisons). Of these, G:F was numerically increased ($P > 0.10$) in 18 comparisons (average of

2.7%) and numerically decreased in 9 comparisons (average of 2.3%) compared to control pigs. Most comparisons (19 comparisons) had added betaine levels of 0.1 and 0.125%; therefore, the effect of betaine levels is not evaluated. There were insufficient data to support whether different types of basal diets affected the response to betaine for ADG and G:F. However, betaine may have a more beneficial effect on ADG and G:F in limit-fed pigs [176, 177]. In summary, the results suggest that betaine had relatively small positive effects on ADG (1.3% improvement) but may benefit G:F more (2.7% improvement).

Table S13. Studies on the effects of dietary betaine on growth performance.

Authors	Country	Inclusion, %	Sig.[1]	Difference, %[1]	
				ADG	G:F
Smith et al. (1994)	USA	0.100[4]	ADG[3]	5.7	2.4
		0.100[4]	ns	1.1	-1.3
Smith et al. (1994)	USA	0.100	ns	3.3	4.1
Matthews et al. (1998), Exp 1	USA	0.125	ns	-0.5	-3.6
Matthews et al. (1998), Exp 2	USA	0.125	ns	0.0	5.6
Øverland et al. (1999)	Norway	1.050	ns	4.2	2.3
Matthews et al. (2001)	USA	0.250	ns	0.0	0.4
Matthews et al. (2001)	USA	0.125	ns[2]	-3.6	6.7
		0.250		-8.3	3.3
		0.500		-8.3	0.4
Young et al. (2001)	USA	0.140	ns	-1.6	-1.3
Lawrence et al. (2002), Exp 1	USA	0.125[5]	ns	-0.6	2.3
		0.125[5]	ns	3.1	2.7
Lawrence et al. (2002), Exp 2	USA	0.100	ns	0.9	3.8
Siljander-Rasi et al. (2003)	Finland	0.025	ADG[2], GF[2]	-0.5	-0.4
		0.050		6.8	5.6
		0.100		9.7	7.5
Feng et al. (2006)	China	0.125	ns	4.6	1.6
Dunshea et al. (2009)	Australia	0.150	ns	2.1	0.9
Huang et al. (2009)	China	0.125	ADG	5.5	2.6
Nakev et al. (2009)	Bulgaria	0.100[6]	ns	-7.1	n/a
		0.100[6]	ns	-4.5	n/a
Yang et al. (2009)	South Korea	0.200	ADG, G:F	3.3	15.0
		0.400	ADG, G:F	27.5	23.2
		0.600	ADG, G:F	17.6	14.6
Van Heugten (2014), Exp 1	USA	0.200	ns	-2.9	1.2
Van Heugten (2014), Exp 2	USA	0.063	ns[2]	-2.1	-1.3
		0.125		-1.1	-1.6
		0.188		0.8	1.6
Madeira et al. (2015)	USA	0.330	ns	1.1	0.4
Wang et al. (2015)	China	0.100	ns	1.2	0.0

Lothong et al. (2016)	Thailand	0.100	ns	-5.6	-6.3
Mendoza et al. (2017), Exp 1	USA	0.200	ADG	-5.1	-1.1
Mendoza et al. (2017), Exp 2	USA	0.063<br>0.125<br>0.188	ns[2]	-1.2<br>-1.8<br>-0.1	-1.0<br>-3.0<br>0.0
Lan and Kim (2018)	South Korea	0.100	ADG	3.7	2.9

[1]Significant level at $P \leq 0.05$. Difference is calculated as [(treatment value – control value) / control value] * 100%.

[2]Polynomial contrasts were used for statistical analysis.

[3]Significant level at $0.05 < P \leq 0.10$.

[4]The top comparison used a solid form of betaine and the bottom comparison used a liquid form betaine.

[5]Treament diets were fed from 82 to 106 kg in the top comparison and fed from 104 to 116 kg in the bottom comparison.

[6]The top comparison represented male pigs and the bottom comparison represented female pigs.

2.1.2. Carcass Characteristics - Betaine

Back-fat significantly decreased ($P \leq 0.05$) in 3 comparisons (average of 10.7%) compared to control pigs (Table S14). The greatest proportion of the comparisons found no evidence of difference ($P > 0.10$) in BF (29 comparisons). Of these, BF was numerically increased ($P > 0.10$) in 13 comparisons (average of 2.0%) and numerically decreased in 16 comparisons (average of 2.9%) compared to control pigs. Percentage lean significantly increased ($P \leq 0.05$) in 1 comparison (5.2%) compared to control pigs. The greatest proportion of the comparisons found no evidence of difference ($P > 0.10$) in percentage lean (24 comparisons). Of these, percentage lean was numerically increased ($P > 0.10$) in 15 comparisons (average of 3.6%) and numerically decreased in 8 comparisons (average of 1.2%) compared to control pigs. Loin muscle area/depth tended to increase ($0.05 < P \leq 0.10$) in 1 comparison (6.3%) and significantly decreased ($P \leq 0.05$) in 3 comparisons (average of 2.3%) compared to control pigs. The greatest

proportion of the comparisons found no evidence of difference ($P > 0.10$) in LMA/LD (20 comparisons). Of these, LMA/LD was numerically increased ($P > 0.10$) in 10 comparisons (average of 1.9%) and numerically decreased in 10 comparisons (average of 2.2%) compared to control pigs

Table S14. Studies on the effects of dietary betaine on carcass characteristics.

Authors	Country	Inclusion, %	Sig.[1]	Difference, %[1]			
				Yield	BF	percentage lean	LMA/LD
Smith et al. (1994)	USA	0.100[4]	LMA[3]	n/a	-3.2	1.6	6.3
		0.100[4]	ns	n/a	0.8	2.6	8.9
Smith et al. (1994)	USA	0.100	ns	n/a	-3.2	0.5	-1.6
Matthews et al. (1998), Exp 1	USA	0.125	Yield	0.9	1.4	-0.2	0.5
Matthews et al. (1998), Exp 2	USA	0.125	ns	0.0	2.3	-0.6	2.1
Øverland et al. (1999)	Norway	1.050	ns	1.2	0.7	0.8	n/a
Matthews et al. (2001)	USA	0.250	ns	-0.4	4.8	-1.1	-0.4
Matthews et al. (2001)	USA	0.125	BF[2]	-1.5	-4.3	1.2	-3.0
		0.250		1.1	-18.2	5.2	0.7
		0.500		0.1	-12.6	-0.8	-5.6
Lawrence et al. (2002), Exp 1	USA	0.125[5]	ns	0.3	-0.4	0.6	1.5
		0.125[5]	ns	0.0	-0.4	-1.6	-0.4
Lawrence et al. (2002), Exp 2	USA	0.100	BF	n/a	-3.2	n/a	-0.4
Siljander-Rasi et al. (2003)	Finland	0.025	ns[2]	n/a	-3.0	n/a	n/a
		0.050		n/a	1.0	n/a	n/a
		0.100		n/a	-3.0	n/a	n/a
Feng et al. (2006)	China	0.125	ns	0.6	-7.0	2.1	2.2
Dunshea et al. (2009)	Australia	0.150	ns	n/a	4.5	0.9	n/a
Huang et al. (2009)	China	0.125	BF, percentage lean	0.6	-10.3	5.2	n/a
Nakev et al. (2009)	Bulgaria	0.100[6]	ns	ns	0.2	-6.3	2.4
		0.100[6]	ns	ns	5.4	7.4	-4.8
Van Heugten (2014), Exp 1	USA	0.200	ns	-0.4	1.8	-0.2	0.7
Van Heugten (2014), Exp 2	USA	0.063	LD[2]	-0.5	-3.4	0.2	-1.5
		0.125		-0.3	-1.5	0.0	-1.5
		0.188		-0.1	-3.9	0.2	-3.9
Madeira et al. (2015)	USA	0.330	ns	0.2	-1.5	n/a	n/a
Wang et al. (2015)	China	0.100	ns	3.4	0.8	n/a	n/a
Lothong et al. (2016)	Thailand	0.125	BF	n/a	-18.6	n/a	n/a
Mendoza et al. (2017), Exp 1	USA	0.200	ns	0.1	0.9	-0.1	0.2
	USA	0.063	ns[2]	-0.7	0.2	11.4	-2.1

Mendoza et al. (2017), Exp	0.125	-0.4	0.2	11.6	-1.3
2	0.188	-0.3	1.3	12.4	-0.6

[1]Significant level at $P \leq 0.05$. Difference is calculated as [(treatment value – control value) / control value] * 100%.

[2]Polynomial contrasts were used for statistical analysis.

[3]Significant level at $0.05 < P \leq 0.10$.

[5]The top comparison used a solid form of betaine and the bottom comparison used a liquid form betaine.

[4]Treament diets were fed from 82 to 106 kg in the top comparison and fed from 104 to 116 kg in the bottom comparison.

[6]The top comparison represented male pigs and the bottom comparison represented female pigs.

2.2. *Chromium (Cr)*

There were 50 research articles for Cr with 139 comparisons from 9 countries during the grow-finish or finishing period with added dietary levels of 25 to 1,000 μg/kg (1 experiment used 5,000 μg/kg Cr as an overdose trial). Of these, 139 comparisons reported growth performance data, and 133 comparisons reported carcass data. The sources of Cr were Cr picolinate, Cr propionate, Cr nicotinate, Cr methionine, Cr yeast, $CrCl_3$, Cr nanocomposites, Cr sulfate, and Cr bis-glycinate-nicotinamide chelate.

2.2.1. *Growth Performance - Cr*

Average daily gain significantly increased ($P \leq 0.05$) in 14 comparisons (average of 8.9%), tended to increase ($0.05 < P \leq 0.10$) in 4 comparisons (average of 4.6%), significantly decreased ($P \leq 0.05$) in 7 comparisons (average of 7.2%), and tended to decrease ($0.05 < P \leq 0.10$) in 5 comparisons (average of 4.1%) compared to control pigs (Table S15). The greatest proportion of the comparisons found no evidence of difference ($P > 0.10$) in ADG (109 comparisons). Of these, ADG was numerically increased ($P > 0.10$) in 51 comparisons (average of 3.6%) and numerically decreased ($P > 0.10$) in 48 comparisons (average of 2.2%) compared to control pigs. Feed efficiency significantly increased ($P \leq 0.05$) in 14 comparisons (average of 5.2%) and significantly decreased ($P \leq 0.05$) in 7 comparisons (average of 4.3%) compared to control pigs. The greatest proportion of the comparisons found no evidence of difference ($P > 0.10$) in G:F (117 comparisons). Of these, G:F was numerically increased ($P > 0.10$) in 60 comparisons

(average of 3.1%) and numerically decreased ($P > 0.10$) in 41 comparisons (average of 2.1%) compared to control pigs. These studies found no evidence of difference because Cr's effects were not large enough and variation in performance was too great. Also, the basal diets might have provided enough Cr to meet the requirement; therefore, increasing the level of Cr did not have a significant effect on ADG and G:F [195-200]. Overall, the data suggest that Cr positively affected ADG and G:F, but the effects were small and inconsistent. The addition of 50 to 400 μg/kg Cr in diets was most common and had better improvement on ADG and GF compared to the higher levels. However, there were not enough comparisons at greater Cr levels to fully determine the effect of high Cr levels. Moreover, toxicity of Cr at a high inclusion level (5,000 μg/kg) was not observed [201]. Chromium chelated with methionine or in nanoparticle form may provide a more consistently positive effect on ADG; however, Cr form did not seem to affect the consistency of the G:F response. According to our database, Cr minorly improved ADG and G:F of pigs (approximately 1% improvement). This is in agreement with a meta-analysis that analyzed data from 31 studies and found that grow-finish pigs fed 200 to 500 μg/kg Cr had improved ($P \leq 0.05$) ADG and G:F compared to the control pigs [202].

Table S15. Studies on the effects of dietary chromium on growth performance.

Authors	Country	Source	Inclusion, μg/kg	Sig.[1]	Difference, % ADG	Difference, % G:F
Page et al. (1993), Exp. 1	USA	Picolinate	25	ADG[2]	0.0	6.9
			50		4.1	4.5
			100		-5.9	-0.3
			200		7.8	5.5
Page et al. (1993), Exp. 2	USA	Picolinate	100	ADG[2]	-1.2	2.1
			200		-0.7	-0.3
			400		-6.2	5.4
			800		-9.1	-0.9

Page et al. (1993), Exp. 3	USA	$CrCl_3$	200[4]	ns	6.0	6.4
			200[4]	ns	4.2	1.2
	USA	Picolinate	100 and 200	ns	5.9	-2.7
Smith et al. (1994)	USA	Nicotinate	200	ns	2.0	1.7
Boleman et al. (1995)	USA	Picolinate	200[5]	ADG	-6.4	0.0
			200[5]	ns	-5.8	3.2
Lindemann et al. (1995), Exp. 1	USA	Picolinate	250	ns[2]	1.0	8.1
			500		-3.1	6.4
Lindemann et al. (1995), Exp. 2	USA	Picolinate	200[4]	ns	-2.4	6.8
			200[4]	ns	-1.2	-2.3
Lindemann et al. (1995), Exp. 3	USA	Picolinate	100	ns[2]	1.2	-0.3
			500		1.2	-2.9
			1000		-2.4	-1.1
Mooney and Cromwell (1995)	USA	Picolinate	200	ADG[3]	5.4	1.9
Smith et al. (1996)	USA	Nicotinate	200	ns	-1.7	-0.2
Kornegay et al. (1997)	USA	Picolinate	200	ns	2.5	n/a
Min et al. (1997)	South Korea	Picolinate	100	ns	-0.7	0.3
			200	ns	-3.3	0.3
			400	ns	-0.5	2.5
Mooney and Cromwell (1997)	USA	Picolinate	200	ns	2.3	1.4
		$CrCl_3$	5,000	ns	-1.1	-1.4
Ward et al. (1997)	USA	Picolinate	400[4]	ns	1.9	5.7
			400[4]	ns	-2.1	-5.4
			400[4]	ns	4.0	3.9
			400[4]	ns	-1.7	-2.7
Lien et al. (1998)	Taiwan	Picolinate	200	ns	-1.9	10.3
O'Quinn et al. (1998)	USA	Nicotinate	50	ADG[2,3]	4.6	1.1
			100		-2.1	-0.9
			200		-3.6	-1.7
			400		-3.2	0.2
		Picolinate	200	ns	-0.8	-1.6
Lemme et al. (1999)	USA	Yeast	200	ADG[3], G:F	5.9	6.5
			400	ns	0.1	1.8
			800	ns	-0.7	1.1
Mooney and Cromwell (1999), Exp.1	USA	Picolinate	200	ns	-0.6	-2.6
Mooney and Cromwell (1999), Exp.2	USA	Picolinate	200	ns	-1.2	1.1
O'Quinn et al. (1999)	USA	Nicotinate	50[4]	G:F	0.7	4.4
			50[4]	G:F	4.7	6.8
Hanczakowska et al. (1999)	Poland	Yeast	(0.03%)	G:F	2.2	-4.7
		Picolinate	200[4]	ns	-0.5	-0.3
			200[4]	ns	0.0	0.0
Matthews et al. (2001)	USA	Picolinate	200	ns	0.0	-1.4
		Propionate	200	ns	-1.1	2.4
Xi et al. (2001)	USA	Picolinate	200	ns	3.6	3.1
Matthews et al. (2003)	USA	Propionate	200	ns	-0.5	1.4
Shelton et al. (2003), Exp. 1	USA	Propionate	50	ns[2]	-2.5	3.3
			100		0.0	3.3

			200		-2.5	3.3
			200[4]	ns	-2.5	3.3
			200[4]	ns	-2.6	3.6
Shelton et al. (2003), Exp. 2	USA	Propionate	100	ns[2]	1.0	-5.6
			200		-1.0	-2.8
			300		-1.0	-2.8
Waylan et al. (2003)	USA	Nicotinate	50	G:F	2.9	5.4
Groesbeck et al. (2004)	USA	Picolinate	100	ns[2]	-2.6	1.8
			200		-3.7	-1.5
		Propionate	100	ns[2]	0.0	0.3
			200		-1.6	-0.9
Wang and Xu (2004)	China	Nano Cr	200	G:F	5.6	3.7
Matthews et al. (2005)	USA	Propionate	200	ns	0.0	2.6
Amoikon et al. (2006)	USA	Picolinate	200[4]	ns	2.5	-3.4
			200[4]	ns	-5.6	0.0
			200[4]	ns	-2.3	0.0
Khajarern et al. (2006)	Thailand	Bisglycinate-nicotinamide chelate	200	ns	-0.1	0.3
			400	ns	1.1	1.4
Bergstrom et al. (2008)	USA	Propionate	200	ns	1.0	1.2
Lindemann et al. (2008)	USA	Picolinate	5,000	ns	6.0	0.6
		Propionate	5,000	ns	5.8	2.3
		Methionine	5,000	ns	0.3	-2.9
		Yeast	5,000	ADG	8.6	0.6
Wang et al. (2008)	China	Picolinate	200	ADG	9.8	4.8
Jackson et al. (2009)	USA	Propionate	200[4]	ns	-0.8	1.5
			200[4]	ns	-2.8	-5.1
			200[4]	ns	6.2	1.4
Park et al. (2009)	South Korea	$CrCl_3$	200	ns	3.1	2.0
		Picolinate	200	ADG, G:F	7.8	6.2
		Methionine	100	ADG, G:F	4.7	3.2
			200	ADG, G:F	6.3	6.2
Wang et al. (2009)	China	$CrCl_3$	200	ns	-0.3	-2.5
		Picolinate	200	ns	2.3	-2.0
		Nano $CrCl_3$	200	ns	6.4	4.8
Wang et al. (2009)	China	$CrCl_3$	200	ns	-0.3	0.9
		Nano Cr	200	ADG, G:F	6.3	9.5
Li et al. (2013)	China	Methionine	300	ADG[2], G:F[2]	4.1	-1.7
			600		16.0	-3.6
			900		20.5	-4.6
Panaite et al. (2013)	Romania	Picolinate	200	ns	-9.0	-3.3
			400	ADG, G:F	-21.1	-10.3
Hung et al. (2014)	Australia	Nano Picolinate	400	ADG[3]	5.3	-0.4
Peres et al. (2014)	Brazil	Sulfate	200	ns	-0.9	0.4
		Methionine	200	ADG, G:F	5.3	7.3
Wang et al. (2014)	China	Cr chitosan nanoparticles	100	G:F[2]	1.3	3.4
			200		-0.1	4.1
			400		-0.5	3.4
Tian et al. (2015)	China	Methionine	100	ns[2]	2.9	-1.2
			200		4.3	0.3
			400		1.4	-1.5
			800		1.4	0.0

Li et al. (2017)	Taiwan	$CrCl_3$	200	ns	13.6	0.0
		Picolinate	200	ns	13.6	3.6
		Nano $CrCl_3$	200	ns	10.6	0.0
		Nano picolinate	200	ADG	21.2	3.6
Marcolla et al. (2017)	Brazil	Yeast	400[4]	ns	-4.0	-3.9
			400[4]	ns	-4.0	0.0
Xu et al. (2017)	China	Methionine	200	ns	1.4	4.4
Jin et al. (2018)	China	Methionine	200[4]	ns	-3.7	-1.0
			200[4]	ns	-0.9	5.1
Gebhardt et al. (2019), Exp. 1	USA	Propionate	200	ns	0.6	-1.3
Gebhardt et al. (2019), Exp. 2	USA	Propionate	200[4]	ns	0.0	2.8
			200[4]	ns	0.0	0.0
			200[4]	ns	2.3	0.0
Gebhardt et al. (2019), Exp. 1	USA	Propionate	100	G:F[2]	1.1	2.5
			200		0.0	0.0
			100/200	ns	1.1	0.0
			200/100	ns	0.0	0.0
Gebhardt et al. (2019), Exp. 2	USA	Propionate	200/100	ns	1.1	0.0
			200	ADG	2.2	0.0
Lien and Lan (2019)	Taiwan	Picolinate	200	ns	11.3	1.1
		Nano picolinate	200	ns	7.6	-3.7
Mayorga et al. (2019)	USA	Propionate	200	ns	3.4	4.0
da Silva et al. (2021)	Brazil	Yeast	800	ns	2.0	7.1
		Picolinate	480	ns	-4.9	7.5
Santos et al. (2021)	USA	Propionate	200[4]	ADG[3], G:F	-1.0	-2.3
			200[4]	ADG[3], G:F	-3.2	-2.9
Alencar et al. (2022)	Brazil	Yeast	800[4]	ns	-3.9	-1.9
			800[4]	ns	2.0	-1.5

[1]Significant level at $P \le 0.05$. Difference is calculated as [(treatment value – control value) / control value] * 100%.

[2]Polynomial contrasts were used for statistical analysis.

[3]Significant level at $0.05 < P \le 0.10$.

[4]For experiments using factorial treatment structures, if the interaction of factors of either interested variable was observed the effect of the feed additive within each level of the other factor is included within the database.

[5]The comparison was the result of Cr fed in grow-finish phase and bottom comparison was the result of Cr fed in finish phase.

2.2.2. *Carcass Characteristics - Cr*

Back-fat significantly increased ($P \le 0.05$) in 2 comparisons (average of 8.0%), tended to increase ($0.05 < P \le 0.10$) in 5 comparisons (average of 6.3%), significantly decreased ($P \le 0.05$)

in 22 comparisons (average of 14.4%), and tended to decrease ($0.05 < P \leq 0.10$) in 7 comparisons (average of 12.4%) compared to control pigs (Table S16). The greatest proportion of the comparisons found no evidence of difference ($P > 0.10$) in BF (97 comparisons). Of these, BF was numerically increased ($P > 0.10$) in 42 comparisons (average of 4.2%) and numerically decreased ($P > 0.10$) in 53 comparisons (average of 6.4%) compared to control pigs. Percentage lean significantly increased ($P \leq 0.05$) in 20 comparisons (average of 6.6%), tended to increase ($0.05 < P \leq 0.10$) in 1 comparison (5.0%), and significantly decreased ($P \leq 0.05$) in 2 comparisons (average of 4.1%) compared to control pigs. The greatest proportion of the comparisons found no evidence of difference ($P > 0.10$) in percentage lean (82 comparisons). Of these, percentage lean was numerically increased ($P > 0.10$) in 43 comparisons (average of 1.9%) and numerically decreased ($P > 0.10$) in 36 comparisons (average of 1.2%) compared to control pigs. Loin muscle area/depth significantly increased ($P \leq 0.05$) in 23 comparisons (average of 13.9%) and significantly decreased ($P \leq 0.05$) in 1 comparison (11.6%) compared to control pigs. The greatest proportion of the comparisons found no evidence of difference ($P > 0.10$) in LMA/LD (101 comparisons). Of these, LMA/LD was numerically increased ($P > 0.10$) in 61 comparisons (average of 3.2%) and numerically decreased ($P > 0.10$) in 38 comparisons (average of 3.0%) compared to control pigs. According to the database, Cr decreased BF, and increased percentage lean, and LMA/LD of grow-finish pigs in more than 60% of the comparisons, and the effects were observed across all inclusion levels (25 to 5,000 μg/kg). Additionally,

increasing the Cr level improved the carcass characteristics linearly within the 50 to 400 μg/kg inclusion (approximately 80% of all comparisons). Different Cr sources may not have the same effect on carcass characteristics. Contrary to the overall effects, Cr nicotinate increased BF by 2.2% (13 comparisons), and $CrCl_3$ decreased LMA/LD by 2.2% (6 comparisons). The meta-analysis conducted by Sales and Jančík [202] also found Cr reduced ($P < 0.001$) 10th rib BF and increased ($P < 0.001$) percentage lean and LMA.

Table S16. Studies on the effects of dietary chromium on carcass characteristics.

Author	Country	Source	Inclusion, µg/kg	Sig.[1]	Difference, %[1]			
					Yield	BF	percentage lean	LMA/LD
Page et al. (1993), Exp. 1	USA	Picolinate	25	ns[2]	n/a	-14.5	3.4	2.9
			50		n/a	-17.7	3.0	1.1
			100		n/a	-6.0	1.9	-2.0
			200		n/a	-13.8	2.6	6.6
Page et al. (1993), Exp. 2	USA	Picolinate	100	Yield[2,3], BF[2], percentage lean[2], LMA[2]	0.8	-25.7	8.5	18.8
			200		2.3	-16.5	5.8	17.4
			400		3.3	-30.2	11.0	22.6
			800		3.2	-21.9	8.7	18.5
Page et al. (1993), Exp. 3	USA	$CrCl_3$	200[4]	ns	0.0	-5.5	0.0	-1.0
			200[4]	ns	-1.1	5.4	-1.9	-3.5
		Picolinate	100 and 200	BF, percentage lean, LMA	0.1	-19.7	5.6	21.4
Smith et al. (1994)	USA	Nicotinate	200	BF	n/a	-8.8	4.1	3.3
Lindemann et al. (1995)	USA	Picolinate	250	ns[2]	n/a	2.4	n/a	3.3
			500		n/a	9.0	n/a	3.8
Lindemann et al. (1995)	USA	Picolinate	200[4]	BF, percentage lean, LMA	n/a	-17.3	8.9	15.9
			200[4]	BF, percentage lean, LMA	n/a	-10.3	4.7	6.6
Lindemann et al. (1995)	USA	Picolinate	100	BF[2], percentage lean[2], LMA[2]	n/a	-5.5	1.8	3.3
			500		n/a	12.7	-7.4	-11.6
			1,000		n/a	-13.9	5.8	5.3
Mooney and Cromwell (1995)	USA	Picolinate	200	ns	n/a	-0.5	n/a	-2.3
Boleman et al. (1995)	USA	Picolinate	200[5]	ns	0.1	7.6	-0.5	1.7
			200[5]	percentage lean[3]	0.1	-8.9	5.0	7.3
Smith et al. (1996)	USA	Nicotinate	200	ns	0.0	2.1	0.5	-1.1
Kornegay et al. (1997)	USA	Picolinate	200	ns	-0.4	3.9	0.0	6.8
Mooney and Cromwell (1997)	USA	Picolinate	200	LMA[3]	-0.5	0.3	2.1	6.3
		$CrCl_3$	5000	LMA[3]	-0.4	-3.0	1.9	6.2
Ward et al. (1997)	USA	Picolinate	400[4]	ns	0.5	-6.5	-0.2	-1.3

			400[4]	ns	-0.4	0.0	-0.2	-4.3
			400[4]	ns	-0.4	3.8	0.0	2.2
			400[4]	ns	0.1	15.0	-1.4	0.0
Min et al. (1997)	South Korea	Picolinate	100	ns	0.3	-4.9	n/a	1.8
			200	ns	0.4	-15.9	n/a	6.6
			400	ns	-0.3	-11.4	n/a	3.1
Lien et al. (1998)	Taiwan	Picolinate	200	BF, LMA	n/a	-9.4	n/a	12.1
O'Quinn et al. (1998), Exp.1 (barrow)	USA	Nicotinate	50	ns[2]	0.2	2.0	-1.3	-1.3
			100		-1.2	8.1	-2.3	-5.7
			200		0.0	2.0	-0.4	-4.9
			400		0.9	-1.0	0.2	0.6
O'Quinn et al. (1998), Exp. 1 (gilt)	USA	Nicotinate	50	Yield[2]	0.0	5.5	-2.0	-3.9
			100		0.9	6.6	-1.2	1.9
			200		1.5	6.6	-1.6	-2.5
			400		0.3	4.4	1.1	2.7
O'Quinn et al. (1998), Exp. 2[12]	USA	Picolinate	200	ns	0.0	3.8	-1.1	-1.9
Hanczakowska et al. (1999)	Poland	Yeast	(0.03%)	ns	n/a	1.4	-0.3	2.0
		Picolinate	200[4]	ns	n/a	-1.8	1.9	4.9
			200[4]	ns	n/a	4.7	-2.5	-5.1
Mooney and Cromwell (1999), Exp. 1	USA	Picolinate	200	ns	0.2	-0.3	0.9	5.4
Mooney and Cromwell (1999), Exp. 2	USA	Picolinate	200	ns	-0.2	3.8	-1.6	-1.8
O'Quinn et al. (1999)	USA	Nicotinate	50[4]	ns	0.7	-0.5	0.1	2.3
			50[4]	ns	-0.7	1.0	0.5	2.6
Lemme et al. (1999)	USA	Yeast	200	Yield[3]	-1.6	5.0	n/a	n/a
			400	ns	0.2	-1.1	n/a	n/a
			800	ns	0.6	3.9	n/a	n/a
Matthews et al. (2001), Exp. 1	USA	Picolinate	200	ns	0.4	-7.7	-2.8	0.7
		Propionate	200	ns	-0.3	-7.7	-0.6	6.1
Xi et al. (2001)	USA	Picolinate	200	BF, percentage lean, LMA	1.2	-10.9	7.6	15.6
Matthews et al. (2003)	USA	Propionate	200	ns	-0.2	0.6	-1.8	-1.0
Shelton et al. (2003), Exp. 1	USA	Propionate	50	ns[2]	0.3	-3.5	2.4	3.8
			100		0.1	2.0	-1.4	0.7

			200		0.1	-2.3	3.0	9.9
			200[4]	ns	0.1	-2.3	3.0	9.9
			200[4]	ns	-1.5	-4.9	0.7	-6.1
Shelton et al. (2003), Exp. 2	USA	Propionate	100	ns[2]	-0.1	7.8	-2.4	-2.6
			200		-0.2	3.7	0.1	2.3
			300		0.2	1.8	0.1	1.7
Waylan et al. (2003)	USA	Nicotinate	50	ns	0.0	0.2	0.3	2.5
Wang and Xu (2004)	China	Nano Cr	200	BF, percentage lean, LMA	1.2	-18.2	14.1	20.0
Matthews et al. (2005)	USA	Propionate	200	ns	-0.4	10.2	-0.6	-4.3
Amoikon et al. (2006)	USA	Picolinate	200[4]	ns	-1.9	-2.3	-0.5	-0.9
			200[4]	ns	-0.8	7.2	-3.5	-7.0
			200[4]	ns	1.9	-0.3	0.5	-1.1
Khajarern et al. (2006)	Thailand	Bisglycinate-nicotinamide chelate	200	Yield[2], BF[2], LMA[2]	0.0	-4.5	2.2	5.9
			400		0.9	-7.3	3.1	7.3
Bergstrom et al. (2008)	USA	Propionate	200	ns	0.7	1.4	-0.7	-3.3
Wang et al. (2008)	China	Picolinate	200	LMA	2.7	-10.3	n/a	17.3
Lindemann et al. (2008)	USA	Tripicolinate	5,000	ns	0.6	-7.3	n/a	2.0
		Propionate	5,000	ns	1.1	-16.0	n/a	0.2
		Methionine	5,000	ns	0.9	-3.8	n/a	-2.6
		Yeast	5,000	ns	0.3	-10.3	n/a	1.3
Jackson et al. (2009)	USA	Propionate	200[4]	BF[3]	0.5	-6.3	2.6	6.1
			200[4]	BF[3]	0.2	-9.1	-2.4	3.1
			200[4]	BF[3]	1.1	1.6	-1.5	3.2
Park et al. (2009)	South Korea	$CrCl_3$	200	ns	-1.4	-12.6	1.6	-5.6
		Picolinate	200	ns	0.9	-13.7	3.2	1.6
		Methionine	100	ns	1.2	-15.5	3.1	2.1
			200	BF, percentage lean	0.7	-31.4	8.8	2.5
Wang et al. (2009)	China	$CrCl_3$	200	ns	1.3	-2.7	3.5	-5.4
		Picolinate	200	LMA	3.1	-10.3	1.7	17.3
		Nano $CrCl_3$	200	BF, percentage lean, LMA	1.5	-24.3	10.6	20.2
Panaite et al. (2013)	Romania	Picolinate	200	ns	n/a	-3.9	0.9	n/a

			400	ns	n/a	-16.5	2.9	n/a
Li et al. (2013)	China	Methionine	300	BF[2,3], LMA[2]	-1.0	-15.4	3.4	7.2
			600		0.3	-19.1	7.2	13.2
			900		-0.1	-22.8	7.3	13.1
Hung et al. (2014)	Australia	Nano Tripicolinate	400	ns	0.0	0.0	n/a	n/a
Peres et al. (2014)	Brazil	Cr sulfate	200	ns	-0.4	-0.6	n/a	-0.4
		Methionine	200	ns	-0.4	-3.8	n/a	0.1
Wang et al. (2014)	China	Chitosan nanoparticles	100	BF[2], percentage lean[2], LMA[2]	1.1	-5.2	3.2	13.5
			200		1.1	-8.1	3.7	15.8
			400		1.0	-7.6	3.2	11.5
Tian et al. (2015)	China	Methionine	100	BF[2,3]	-1.5	7.4	-0.8	-1.4
			200		-1.2	14.5	1.9	16.3
			400		-0.8	-10.9	3.8	6.1
			800		-0.5	-3.3	2.1	6.8
Li et al. (2017)	Taiwan	$CrCl_3$	200	ns	1.2	-0.4	n/a	-3.7
		Picolinate	200	BF	1.2	-9.6	n/a	-3.7
		Nano $CrCl_3$	200	ns	0.9	-0.9	n/a	-3.3
		Nano Picolinate	200	BF	0.9	-9.6	n/a	0.2
Marcolla et al. (2017)	Brazil	Yeast	400[4]	ns	-0.1	-24.5	n/a	-0.3
			400[4]	ns	-0.3	10.2	n/a	-6.4
Xu et al. (2017)	China	Methionine	200	ns	1.1	-5.2	n/a	2.1
Jin et al. (2018)	China	Methionine	200[4]	Yield	3.2	11.9	n/a	n/a
			200[4]	ns	0.9	-8.2	n/a	n/a
Mayorga et al. (2019)	USA	Propionate	200	ns	n/a	-0.8	n/a	0.4
Gebhardt et al. (2019), Exp. 1	USA	Propionate	200	BF, percentage lean	0.6	3.3	-0.8	-1.0
Gebhardt et al. (2019), Exp. 2	USA	Propionate	200[4]	ns	-0.3	0.8	-0.1	0.0
			200[4]	ns	0.3	0.7	-0.1	0.1
			200[4]	ns	-0.5	1.5	-0.5	-1.9
Gebhardt et al. (2019), Exp. 1	USA	Propionate	100	ns[2]	0.1	0.3	0.1	1.3
			200		-0.5	-0.4	0.2	0.8
			100/200	ns	-0.1	0.6	0.1	1.2
			200/100	ns	-0.3	-0.2	0.2	1.3
Gebhardt et al. (2019), Exp. 2	USA	Propionate	200/100	ns	0.0	-2.2	0.6	1.2
			200/200	Yield	-0.4	1.1	-0.2	0.5
Lien and Lan (2019)	Taiwan	Picolinate	200	ns	-1.5	-9.7	-0.7	0.0
		Nano Picolinate	200	ns	-0.6	-11.1	-1.4	4.5

da Silva et al. (2021)	Brazil	Yeast	800	percentage lean	n/a	0.7	5.9	3.2
		Picolinate	480	percentage lean	n/a	-12.8	7.9	1.7
Santos et al. (2021)	USA	Propionate	200[4]	BF[3]	0.4	6.1	-0.7	1.5
			200[4]	BF[3]	-0.3	1.9	-0.6	-1.4
Alencar et al. (2022)	Brazil	Yeast	800[4]	ns	1.2	-1.4	-0.4	-2.5
			800[4]	ns	-1.0	-1.5	0.2	-1.9

[1]Significant level at $P \leq 0.05$. Difference is calculated as [(treatment value – control value) / control value] * 100%.

[2]Polynomial contrasts were used for statistical analysis.

[3]Significant level at $0.05 < P \leq 0.10$.

[4]For experiments using factorial treatment structures, if the interaction of factors of either interested variable was observed the effect of the feed additive within each level of the other factor is included within the database.

[5]The 2 experimental diets with Cr were fed in grow-finish and finish phase respectively.

2.3. *Conjugated Linoleic Acid (CLA)*

There were 46 research articles for CLA with 73 comparisons from 15 countries during the grow-finish or finishing period with added dietary levels of 0.07 to 2.72%. Of these, 55 comparisons reported growth performance data, and 65 comparisons reported carcass data.

2.3.1. *Growth Performance - CLA*

Average daily gain significantly increased ($P \leq 0.05$) in 5 comparisons (average of 7.2%), tended to increase ($0.05 < P \leq 0.10$) in 1 comparison (3.6%), and significantly decreased ($P \leq 0.05$) in 3 comparisons (average of 7.8%) compared to control pigs (Table S17). The greatest proportion of the comparisons found no evidence of difference ($P > 0.10$) in ADG (53 comparisons). Of these, ADG was numerically increased ($P > 0.10$) in 34 comparisons (average of 3.7%) and numerically decreased ($P > 0.10$) in 17 comparisons (average of 4.1%) compared to control pigs. Feed efficiency significantly increased ($P \leq 0.05$) in 13 comparisons (average of 4.5%), tended to increase ($0.05 < P \leq 0.10$) in 6 comparisons (average of 8.8%), and significantly decreased ($P \leq 0.05$) in 1 comparison (2.8%) compared to control pigs. The greatest proportion of the comparisons found no evidence of difference ($P > 0.10$) in G:F (37 comparisons). Of these, G:F was numerically increased ($P > 0.10$) in 24 comparisons (average of 4.6%) and numerically decreased ($P > 0.10$) in 9 comparisons (average of 2.3%) compared to control pigs. Overall, the results suggest that CLA had positive effects on ADG and G:F (65 and 75% of all the comparisons); however, the effects of CLA were often not significant enough to statistically

improve ($P < 0.10$) ADG (10% of all the comparisons). On the other hand, CLA improved ($P <$ 0.10) G:F in 33% of all the comparisons. Increasing CLA concentrations did not increasingly improve the ADG and G:F response. Contrary to what is concluded herein, in a meta-analysis by Wang et al. [245] found no evidence of difference ($P > 0.05$) in ADG, ADFI, and G:F when pigs were fed CLA or linseed supplementation; however, only seven research articles were included in their meta-analysis. Different basal diets may affect the response to CLA on ADG and G:F. Diets with wheat as the main ingredient (18 comparisons) had a greater percentage of improvement in ADG (3.7%) and G:F (5.4%) compared to diets with corn as the main ingredient [ADG decreased 0.1% (32 comparisons) and G:F increased 2.8% (28 comparisons)].

Table S17. Studies on the effects of dietary CLA on growth performance.

Author	Country	Inclusion, %	Sig.[1]	Difference, %[1]	
				ADG	G:F
Dugan et al. (1997)	Canada	1.00	G:F[3]	0.0	6.2
Ostrowska et al. (1999)	Australia	0.07	ns[2]	10.2	5.8
		0.14		6.3	6.7
		0.28		8.4	8.8
		0.41		1.4	3.0
		0.55		2.8	6.7
O'Quinn et al. (2000)	USA	0.30	ns	-5.8	0.0
Bee (2001)	Switzerland	1.20	ns	7.5	6.8
Eggert et al. (2001)	USA	0.60	ADG	-10.2	-7.4
Dugan et al. (2001)	Canada	0.16[4]	ns	1.5	4.0
		0.33[4]	ns	4.0	2.1
		0.16[4]	ns	5.0	0.3
		0.33[4]	ns	0.7	2.0
Thiel-Cooper et al. (2001)	USA	0.07	ns	-1.3	4.3
		0.15	ns	1.2	6.0
		0.30	ns	3.4	5.1
		0.60	ADG, G:F	8.2	9.1
Wiegand et al. (2001)	USA	0.75	G:F	1.9	6.1
Barowicz et al. (2002)	Poland	1.20	ns	4.3	10.5
		1.20	ns	1.2	0.8
Dunshea et al. (2002), Exp. 1	Australia	0.22	ns	1.4	4.1
Dunshea et al. (2002), Exp. 2	Australia	0.22	ns	-1.4	-0.7
Tischendorf et al. (2002)	Germany	1.08	ns	2.0	0.3
Wiegand et al. (2002)	USA	0.75[5]	G:F[2]	0.5	2.7

		0.75[5]		1.9	3.3
		0.75[5]		-1.3	1.2
Ostrowska et al. (2003)	Australia	0.07	G:F[2,3]	6.1	6.7
		0.14		5.8	10.0
		0.28		6.8	16.7
		0.41		-0.1	3.3
		0.55		-2.0	10.0
Sun et al. (2004)	China	1.36	ADG[2], G:F[2]	7.7	3.9
		2.72		14.1	5.2
Barowicz et al. (2005)	Poland	1.20	ns	1.2	n/a
Lauridsen et al. (2005)	Denmark	0.30	ADG[3], G:F	3.6	4.8
Weber et al. (2006)	USA	0.60	G:F	3.4	4.0
Bee et al. (2008)	Switzerland	0.60	ns	2.5	0.0
Corino et al. (2008)	Italy	0.38	ns	2.2	n/a
Martin et al. (2008)	Spain	0.56	ns	5.1	5.9
		1.12	ns	5.7	5.9
White et al. (2009)	USA	0.60	ns	5.4	-1.4
Jiang et al. (2010)	China	1.00	ADG[2]	-7.9	0.0
		2.00		-5.3	-1.6
Han et al. (2011)	China	0.36	ns[2]	-6.6	n/a
		0.71		-14.5	n/a
		1.09		-9.2	n/a
Lee et al. (2011)	South Korea	0.59	ns	3.7	12.5
Barnes et al. (2012)	USA	0.60	G:F	-3.2	-2.8
Go et al. (2012)	USA	0.80	ns	-3.2	-2.7
Martinez-Aispuro et al. (2012)	Mexico	1.2/0.5/0.2	ns	-9.4	-2.0
Rickard et al. (2012)	USA	0.36	G:F	1.6	5.8
Pompeu et al. (2013)	USA	0.60[4]	ADG, G:F	1.6	2.2
		0.60[4]	ADG, G:F	4.4	2.3
Tous et al. (2013)	Spain	2.51	ns	0.0	-2.5
Martínez-Aispuro et al. (2014)	Mexico	0.60	ns	-1.9	-2.2
Wang et al. (2015)	China	0.60	ns	1.2	0.0
Marcolla et al. (2017)	Brazil	0.28[4]	ns	5.0	3.9
		0.28[4]	G:F	4.9	8.1
Upadhaya et al. (2017)	South Korea	0.28	ns	-0.5	1.2
		0.56	ns	-0.8	-0.3
Panisson et al. (2020)	Brazil	0.18	ns	-3.4	2.0
		0.36	ns	-5.5	2.0

[1]Significant level at $P \le 0.05$. Difference is calculated as [(treatment value – control value) / control value] * 100%.

[2]Polynomial contrasts were used for statistical analysis.

[3]Significant level at $0.05 < P \le 0.10$.

[4]For experiments using factorial treatment structures, if the interaction of factors of either interested variable was observed the effect of the feed additive within each level of the other factor is included within the database.

[5]CLA was fed in the 3 treatment diets during the last 29, 56, or 87 kg of BW gain before slaughter respectively.

2.3.2. Carcass Characteristics - CLA

Back-fat significantly decreased ($P \leq 0.05$) in 22 comparisons (average of 15.4%) and tended to decrease ($0.05 < P \leq 0.10$) in 5 comparisons (average of 6.5%) compared to control pigs (Table S18). Approximately half of the studies found no evidence of difference ($P > 0.10$) in BF (32 comparisons). Of these, BF was numerically increased ($P > 0.10$) in 14 comparisons (average of 4.0%) and numerically decreased ($P > 0.10$) in 16 comparisons (average of 6.1%) compared to control pigs. Percentage lean significantly increased ($P \leq 0.05$) in 14 comparisons (average of 4.9%) compared to control pigs. The greatest proportion of the comparisons found no evidence of difference ($P > 0.10$) in percentage lean (23 comparisons). Of these, percentage lean was numerically increased ($P > 0.10$) in 16 comparisons (average of 1.9%) and numerically decreased ($P > 0.10$) in 7 comparisons (average of 0.6%) compared to control pigs. Loin muscle area/depth significantly increased ($P \leq 0.05$) in 6 comparisons (average of 7.6%), tended to increase ($0.05 < P \leq 0.10$) in 1 comparison (3.7%), significantly decreased ($P \leq 0.05$) in 1 comparison (5.9%), and tended to decrease ($0.05 < P \leq 0.10$) in 1 comparison (4.8%) compared to control pigs. The greatest proportion of the comparisons found no evidence of difference ($P > 0.10$) in LMA/LD (29 comparisons). Of these, LMA/LD was numerically increased ($P > 0.10$) in 14 comparisons (average of 3.0%) and numerically decreased ($P > 0.10$) in 15 comparisons (average of 3.0%) compared to control pigs. The results showed that CLA had significant effects on decreasing BF (73% of all the comparisons and all the significant comparisons) and increasing percentage lean (81% of all the comparisons and all the significant comparisons) of

grow-finish pigs. Increasing CLA concentrations did not increasingly improve the carcass characteristics. Moreover, different basal diets may affect the response to CLA on BF and percentage lean. Diets with wheat as the main ingredient had a bigger percentage of reduction in BF (13.8%; 9 comparisons) and an improvement in percentage lean (3.7%; 11 comparisons) compared to diets with corn as the main ingredient [BF decrease 7.4% (37 comparisons) and percentage lean increased 2.3% (16 comparisons)]. These results suggest that CLA has the potential to reduce BF and increase percentage lean more consistently compared to other feed additives considered in this review.

Table S18. Studies on the effects of dietary CLA on carcass characteristics.

Author	Country	Inclusion, %	Sig.[1]	Difference, %[1]			
				Yield	BF	percentage lean	LMA/LD
Dugan et al. (1997)	Canada	1.00	percentage lean	n/a	n/a	2.3	n/a
Ostrowska et al. (1999)	Australia	0.07	percentage lean[2]	n/a	n/a	0.7	n/a
		0.14		n/a	n/a	6.0	n/a
		0.28		n/a	n/a	7.2	n/a
		0.41		n/a	n/a	5.4	n/a
		0.55		n/a	n/a	9.1	n/a
O'Quinn et al. (2000)	USA	0.30	ns	-1.4	-5.2	0.4	-4.1
Bee (2001)	Switzerland	1.20	BF	n/a	-20.7	-0.4	1.4
Dugan et al. (2001)	Canada	0.16[4]	percentage lean	n/a	n/a	6.0	n/a
		0.33[4]	percentage lean	n/a	n/a	4.4	n/a
		0.16[4]	ns	n/a	n/a	-0.3	n/a
		0.33[4]	ns	n/a	n/a	1.8	n/a
Eggert et al. (2001)	USA	0.60	ns	-0.1	-8.2	n/a	-2.0
Thiel-Cooper et al. (2001)	USA	0.07	BF	n/a	-18.2	n/a	6.4
		0.15	BF	n/a	-18.2	n/a	2.0
		0.30	ns	n/a	-8.7	n/a	-2.8
		0.60	ns	n/a	-10.1	n/a	-4.7
Wiegand et al. (2001)	USA	0.75	BF	n/a	-7.0	n/a	4.9
Averette Gatlin et al. (2002)	USA	0.60	ns	n/a	-2.6	-0.4	-2.9
Barowicz et al. (2002)	Poland	1.20	ns	-0.9	18.0	5.4	n/a
		1.20	ns	-0.1	-5.3	1.2	n/a
Dunshea et al. (2002), Exp 1	Australia	0.22	BF	0.2	-8.0	n/a	n/a
Dunshea et al. (2002), Exp 2	Australia	0.22	ns	0.7	1.0	n/a	n/a
Tischendorf et al. (2002)	Germany	1.08	percentage lean	-0.4	-7.4	2.6	n/a
Wiegand et al. (2002)	USA	0.75[5]	BF[2], percentage lean[2], LMA[2]	n/a	-14.5	5.0	6.6
		0.75[5]		n/a	-14.5	6.4	11.0
		0.75[5]		n/a	-20.6	7.4	9.2
Corino et al. (2003)	Italy	0.16	BF[3]	0.0	-12.8	n/a	n/a
		0.33	BF[3]	-0.1	-8.9	n/a	n/a
Ostrowska et al. (2003)	Australia	0.07	ns[2]	-1.3	n/a	n/a	n/a
		0.14		-0.6	n/a	n/a	n/a
		0.28		-0.3	n/a	n/a	n/a
		0.41		0.1	n/a	n/a	n/a

		0.55		-2.3	n/a	n/a	n/a
Sun et al. (2004)	China	1.36	BF[2], LMA[2]	n/a	-8.6	n/a	4.6
		2.72		n/a	-10.4	n/a	5.7
Barowicz et al. (2005)	Poland	1.20	BF, LMA	-1.0	-9.5	3.0	8.5
Corino et al. (2005)	Italy	0.38	ns	-1.7	1.8	n/a	n/a
Lauridsen et al. (2005)	Denmark	0.30	ns	n/a	0.4	0.4	1.2
Ostrowska et al. (2005)	Australia	0.07	BF[2]	-1.3	-7.1	n/a	n/a
		0.14		-0.6	-17.8	n/a	n/a
		0.28		-0.3	-17.4	n/a	n/a
		0.41		0.1	-19.1	n/a	n/a
		0.55		-2.3	-23.7	n/a	n/a
Rossi et al. (2005)	Italy	0.38	ns	0.2	n/a	0.4	n/a
Weber et al. (2006)	USA	0.60	BF[3], percentage lean, LMA[3]	-0.6	-7.0	1.8	3.7
Bee et al. (2008)	Switzerland	0.60	BF	n/a	-11.0	0.9	n/a
Corino et al. (2008)	Italy	0.38	ns	-0.8	0.4	-0.4	-3.7
Martin et al. (2008)	Spain	0.56	ns	-0.5	0.0	n/a	n/a
		1.12	ns	-0.2	5.2	n/a	n/a
Cechova et al. (2009)	Czech Republic	1.20	ns	n/a	1.2	n/a	-2.6
Larsen et al. (2009)	USA	0.45	ns	n/a	-6.1	n/a	4.4
White et al. (2009)	USA	0.60	ns	n/a	3.6	2.4	-0.4
Cechova et al. (2010)	Czech Republic	1.20	ns	n/a	n/a	-0.6	n/a
Cordero et al. (2010)	Spain	0.60	ns	-0.9	-2.7	n/a	n/a
Cordero et al. (2010)	Spain	0.30	ns[2]	-1.5	n/a	n/a	n/a
		0.60		-0.8	n/a	n/a	n/a
		1.20		0.1	n/a	n/a	n/a
Jiang et al. (2010)	China	1.00	BF, percentage lean	-0.2	-26.8	4.7	9.3
		2.00	BF	-0.5	-8.5	3.5	-2.0
Han et al. (2011)	China	0.36	Yield[2,3]	3.0	n/a	n/a	n/a
		0.71		3.7	n/a	n/a	n/a
		1.09		2.3	n/a	n/a	n/a
Lee et al. (2011)	South Korea	0.59	ns	-1.9	-2.7	n/a	n/a
Barnes et al. (2012)	USA	0.60	BF, LMA	n/a	-16.0	1.8	-5.9
Go et al. (2012)	USA	0.80	ns	0.5	-1.5	n/a	-0.5
Martinez-Aispuro et al. (2012)	Mexico	--	ns	n/a	1.6	-1.8	-7.5
Rickard et al. (2012)	USA	0.36	BF, LMA	n/a	-12.7	n/a	-4.8
Pompeu et al. (2013)	USA	0.60[4]	Yield, BF[3]	-1.0	-3.0	0.9	2.4

		0.60[4]	Yield, BF[3]	-0.6	-0.9	0.2	0.1
Tous et al. (2013)	Spain	2.51	ns	-0.6	-11.6	5.0	0.8
Bothma et al. (2014)	South Africa	0.15	ns	n/a	-4.0	n/a	n/a
		0.30	ns	n/a	-1.0	n/a	n/a
		0.60	ns	n/a	-14.0	n/a	n/a
Martínez-Aispuro et al. (2014)	Mexico	0.60	ns	n/a	-5.8	-0.3	-4.3
Wang et al. (2015)	China	0.60	ns	3.1	0.0	n/a	5.2
Marcolla et al. (2017)	Brazil	0.28[4]	BF	0.0	-27.5	n/a	2.0
		0.28[4]	ns	-0.2	5.8	n/a	-4.2
Upadhaya et al. (2017)	South Korea	0.28	ns	n/a	-2.3	1.0	0.9
		0.56	ns	n/a	-6.4	1.8	0.9
Panisson et al. (2020)	Brazil	0.18	ns	n/a	4.8	n/a	-0.3
		0.36	ns	n/a	5.2	n/a	-3.6

[1]Significant level at $P \leq 0.05$. Difference is calculated as [(treatment value – control value) / control value] * 100%.

[2]Polynomial contrasts were used for statistical analysis.

[3]Significant level at $0.05 < P \leq 0.10$.

[4]For experiments using factorial treatment structures, if the interaction of factors of either interested variable was observed the effect of the feed additive within each level of the other factor is included within the database.

[5]CLA was fed in the 3 treatment diets during the last 29, 56, or 87 kg of BW gain before slaughter respectively.

2.4. *L-carnitine*

There were 12 research articles for L-carnitine with 29 comparisons from 4 countries during the grow-finish or finishing period with added dietary levels of 25 to 250 mg/kg, with most studies feeding 50 mg/kg. Of these, 24 comparisons reported growth performance data, and 22 comparisons reported carcass data.

2.4.1. *Growth Performance – L-carnitine*

Average daily gain significantly increased ($P \leq 0.05$) in 2 comparisons (average of 3.3%), tended to increase ($0.05 < P \leq 0.10$) in 4 comparisons (average of 3.1%), and significantly decreased ($P \leq 0.05$) in 1 comparison (4.8%) compared to control pigs (Table S19). The greatest proportion of the comparisons found no evidence of difference ($P > 0.10$) in ADG (17 comparisons). Of these, ADG was numerically increased ($P > 0.10$) in 13 comparisons (average of 3.4%) and numerically decreased ($P > 0.10$) in 3 comparisons (average of 2.6%) compared to control pigs. Feed efficiency significantly increased ($P \leq 0.05$) in 1 comparison (2.9%) and tended to increase ($0.05 < P \leq 0.10$) in 3 comparisons (average of 3.7%) compared to control pigs. The greatest proportion of the comparisons found no evidence of difference ($P > 0.10$) in G:F (20 comparisons). Of these, G:F was numerically increased ($P > 0.10$) in 13 comparisons (average of 4.4%) and numerically decreased ($P > 0.10$) in 5 comparisons (average of 2%) compared to control pigs. Overall, the results suggest that L-carnitine has the potential to improve ADG and G:F (79 and 71% of all the comparisons) with relatively large improvement.

Moreover, the beneficial effects of L-carnitine were significant ($P < 0.10$) for ADG and G:F in 25 and 23% of all the comparisons, respectively. There were not enough data to support whether different inclusion levels or types of basal diets affected the response to L-carnitine for ADG and G:F. However, results might suggest that pigs fed L-carnitine were more likely to have improved ADG and G:F when fed in diets without DDGS [290-292], but further research is needed to confirm this. Additionally, environmental factors, such as temperature, humidity, and stress level, may affect L-carnitine response [293] due to the change in feed intake and physiological status.

Table S19. Studies on the effects of dietary L-carnitine on growth performance.

Authors	Country	Inclusion, mg/kg	Sig.[1]	Difference, %[1] ADG	Difference, %[1] G:F
Owen et al. (2001)	USA	50	ns[2]	2.2	-3.1
		125		-1.1	0.0
Owen et al. (2001)	USA	25	ns[2]	3.3	3.1
		50		2.2	6.3
		75		3.3	6.3
		100		6.5	6.3
		125		2.2	6.3
Waylan et al. (2003)	USA	50	ns	0.8	1.3
Bertol et al. (2005)	USA	150	ns	-4.7	-3.6
Han and Thacker (2006)	South Korea	50	ns	5.0	7.1
Chen et al. (2008)	South Korea	250	ns	3.9	3.1
Pietruszka et al. (2009)	Poland	100	ns	1.4	1.2
James et al. (2013)	USA	50	ns	0.0	-0.4
James et al. (2013), Exp. 1	USA	25	ADG[2]	-4.8	0.0
		50		0.6	-1.6
James et al. (2013), Exp. 2	USA	25	G:F[2,3]	2.3	5.5
		50		-1.9	4.1
James et al. (2013), Exp. 3	USA	50	ns	9.4	7.7
James et al. (2013), Exp. 4	USA	50	ADG, G:F	6.0	2.9
Ying et al. (2013)	USA	50[4]	ADG[2,3]	4.9	3.5
		100[4]		3.7	0.6
		50[4]	ADG[2,3]	2.4	-1.2
		100[4]		1.2	4.2
Meng et al. (2018)	USA	50	G:F[3]	1.8	1.5

[1]Significant level at $P \leq 0.05$. Difference is calculated as [(treatment value – control value) / control value] * 100%.

[2]Polynomial contrasts were used for statistical analysis.

[3]Significant level at $0.05 < P \leq 0.10$.

[4]The basal diets were corn-SBM based in the top two comparisons. The basal diets were corn-DDGS-SBM based in the bottom two comparisons.

2.4.2. *Carcass Characteristics – L-carnitine*

Back-fat significantly increased ($P \leq 0.05$) in 3 comparisons (average of 4%), tended to increase ($0.05 < P \leq 0.10$) in 2 comparisons (average of 1.4%), significantly decreased ($P \leq 0.05$) in 2 comparisons (average of 12.5%), and tended to decrease ($0.05 < P \leq 0.10$) in 7 comparisons (average of 4.8%) compared to control pigs (Table S20). Eight comparisons found no evidence of difference ($P > 0.10$) in BF. Of these, BF was numerically increased ($P > 0.10$) in 1 comparison (1.9%) and numerically decreased ($P > 0.10$) in 6 comparisons (average of 5.7%) compared to control pigs. Percentage lean significantly increased ($P \leq 0.05$) in 4 comparisons (average of 3.8%), tended to increase ($0.05 < P \leq 0.10$) in 2 comparisons (average of 1.5%), and significantly decreased ($P \leq 0.05$) in 3 comparisons (average of 1.3%) compared to control pigs. Half of the studies found no evidence of difference ($P > 0.10$) in percentage lean (11 comparisons). Of these, percentage lean was numerically increased ($P > 0.10$) in 7 comparisons (average of 1.5%) and numerically decreased ($P > 0.10$) in 3 comparisons (average of 0.7%) compared to control pigs. Loin muscle area/depth significantly increased ($P \leq 0.05$) in 1 comparison (6.3%) compared to control pigs. The greatest proportion of the comparisons found no evidence of difference ($P > 0.10$) in LMA/LD (20 comparisons). Of these, LMA/LD was numerically increased ($P > 0.10$) in 13 comparisons (average of 4.4%) and numerically decreased ($P > 0.10$) in 6 comparisons (average of 2.3%) compared to control pigs. There were not enough data to support whether different inclusion levels or types of basal diets affected the response to L-

carnitine for carcass characteristics. Overall, the results suggest that L-carnitine is a potential feed additive that had relatively large positive effects on BF, percentage lean, and LMA/LD (68, 65, and 67% of all the comparisons) compared to other feed additives in this review, with 23, 30, and 5% of all the comparisons being significant ($P < 0.10$), respectively.

Table S20. Studies on the effects of dietary L-carnitine on carcass characteristics.

Authors	Country	Inclusion, mg/kg	Sig.[1]	Difference, %[1]			
				Yield	BF	percentage lean	LMA/LD
Owen et al. (2001)	USA	50	BF[2,3], percentage lean[2]	-1.3	-2.6	1.8	1.4
		125		-1.2	-4.3	4.1	9.2
Owen et al. (2001)	USA	25	BF[2,3], percentage lean[2]	0.9	0.6	-2.6	-6.4
		50		-0.4	-9.1	7.6	12.6
		75		-0.7	-5.0	1.9	0.2
		100		-0.1	-2.8	-0.3	-1.1
		125		1.0	2.2	-0.9	0.6
Waylan et al. (2003)	USA	50	ns	0.5	1.9	0.1	2.2
Bertol et al. (2005)	USA	150	ns	n/a	-3.8	n/a	-2.2
Han and Thacker (2006)	South Korea	50	ns	-0.1	-10.5	0.8	n/a
Chen et al. (2008)	South Korea	250	BF	n/a	-18.2	1.8	16.2
Pietruszka et al. (2009)	Poland	100	ns	2.7	-7.4	1.4	-3.4
James et al. (2013), Exp.1	USA	25	ns[2]	-0.6	-3.1	2.2	1.8
		50		-0.7	-2.2	1.7	0.2
James et al. (2013), Exp.2	USA	25	BF[2,3], percentage lean [2,3]	3.0	-2.0	1.2	7.5
		50		0.6	-7.6	1.7	2.0
James et al. (2013), Exp.3	USA	50	LMA	n/a	-7.1	2.4	6.3
Ying et al. (2013)[7]	USA	50[4]	Yield[2,3], BF[2]	1.6	4.8	-0.7	0.5
		100[4]		0.4	3.0	-0.5	-0.3
		50[4]	Yield[2,3], BF[2]	0.3	4.2	-0.7	-0.3
		100[4]		0.1	0.0	0.0	0.0
Meng et al. (2018)	USA	50	BF	-0.2	-6.7	n/a	3.3

[1]Significant level at $P \leq 0.05$. Difference is calculated as [(treatment value – control value) / control value] * 100%.
[2]Polynomial contrasts were used for statistical analysis.
[3]Significant level at $0.05 < P \leq 0.10$.
[4]The basal diets were corn-SBM based in the top two comparisons. The basal diets were corn-DDGS-SBM based in the bottom two comparisons.

3. Feed Additives – Enzymes

This section discusses dietary enzymes used as feed additives in classes of carbohydrases, proteases, phytases, and combination of different types of enzymes (multi-enzymes). There were 86 research articles for enzymes with 165 comparisons from 13 countries during the grow-finish or finishing period which met the requirements for inclusion. Of these, 163 comparisons reported growth performance data, and 107 comparisons reported carcass data. For phytases, its effect in low P diets has been well established, thus, only experiments that utilized diets at/above P requirement were included to discuss the other potential benefits of adding phytases.

3.1. *Growth performance - Carbohydrases*

Average daily gain significantly increased ($P \leq 0.05$) in 15 comparisons (average of 5.3%), tended to increase ($0.05 < P \leq 0.10$) in 5 comparisons (average of 4%), and significantly decreased ($P \leq 0.05$) in 4 comparisons (average of 2.7%) compared to control pigs (Table S21). The greatest proportion of the comparisons found no evidence of difference ($P > 0.10$) in ADG (63 comparisons). Of these, ADG was numerically increased ($P > 0.10$) in 35 comparisons (average of 2.9%) and numerically decreased ($P > 0.10$) in 24 comparisons (average of 3.3%) compared to control pigs. Feed efficiency significantly increased ($P \leq 0.05$) in 8 comparisons (average of 8.5%) and tended to increase ($0.05 < P \leq 0.10$) in 2 comparisons (average of 5.9%) compared to control pigs. The greatest proportion of the comparisons found no evidence of

difference ($P > 0.10$) in G:F (74 comparisons). Of these, G:F was numerically increased ($P > 0.10$) in 46 comparisons (average of 2.9%) and numerically decreased ($P > 0.10$) in 19 comparisons (average of 3.8%) compared to control pigs. Overall, the results suggest that carbohydrases had positive effects on ADG and G:F (63 and 67% of all the comparisons), but the magnitude was small, and most comparisons had no statistical differences. Additionally, in a meta-analysis conducted by Aranda-Aguirre et al. [302], the authors found that β-mannanase and xylanase had no effects ($P > 0.10$) on growth performance of finishing pigs. However, in another meta-analysis by Kiarie et al. [303], the authors found β-mannanase improved ($P < 0.0001$) ADG and G:F of grow-finish pigs. Dietary composition and differences in primary ingredients utilized may affect the effect of carbohydrases on ADG and G:F. Corn diets (32 comparisons) had 2.6% improvement in ADG and 2.2% increase in G:F; Barley diet had 0.4% improvement in ADG (23 comparisons) and 1.7% improvement in G:F (21 comparisons); and wheat diet had 0.9% decrease in ADG (16 comparisons) and 0.2% improvement in G:F (15 comparisons). Moreover, with the limited data, carbohydrases appeared to improve ADG and G:F in some trials with diets that were low in energy and/or AAs, which suggest carbohydrases may be beneficial for the economic efficiency in diets formulated below requirements. Moreover, carbohydrases showed no benefit or negative effects in diets with high level of DDGS (above 15%). Because the production of DDGS utilizes carbohydrases

during the fermentation stage to release the nutrients (starch), there may not be enough available substrates for dietary carbohydrases to be beneficial.

Table S21. Studies on the effects of enzymes on growth performance.

Author	Country	Enzyme	Sig.[1]	Difference, %[1] ADG	Difference, %[1] G:F
Thacker et al. (1988)	Canada	Carbohydrases	ns	2.7	0.6
Thacker et al. (1992)	Canada	Carbohydrases[5]	ns	1.2	1.6
			ns	-8.1	-3.7
Baas and Thacker (1996)	Canada	Carbohydrases[a]	ns	0.0	2.4
		Carbohydrases[b]	ns	-3.5	2.9
		Carbohydrases[c]	ns	-1.2	1.6
		Carbohydrases[d]	ns	-3.5	0.8
Kim et al. (1998)	USA	Carbohydrases	ns	3.2	-0.4
Flis et al. (1998)	Poland	Carbohydrases[5]	ns	3.1	3.3
			ns	4.4	4.7
O'Doherty and Forde (1999)	Ireland	Carbohydrases	ns	-2.6	0.3
Thacker and Campbell (1999)	Canada	Carbohydrases[5]	ns	0.0	0.0
			ns	-2.1	2.4
Mavromichalis et al. (2000), Exp. 1	USA	Carbohydrases	ns	-1.7	3.5
Mavromichalis et al. (2000), Exp. 2	USA	Carbohydrases	ns	2.2	0.0
Grandhi (2001)	Canada	Carbohydrases	G:F	1.2	4.2
Pettey et al. (2002)	USA	Carbohydrases	ADG, G:F[3]	3.6	4.2
Park et al. (2003), Exp. 1	USA	Carbohydrases	ns	2.2	2.7
Park et al. (2003), Exp. 2	USA	Carbohydrases[4]	ADG[2,3]	4.4	2.8
				3.3	2.8
				6.7	5.6
Park et al. (2003), Exp. 3	USA	Carbohydrases[4]	ns[2]	0.0	0.0
				1.1	3.1
				1.1	0.0
Park et al. (2003)	USA	Carbohydrases[5]	ns	2.2	5.9
			ns	-5.4	-4.7
			ns	-5.8	-5.4
			ns	1.6	1.0
			ns	-2.9	-0.5
Flis and Sobotka (2005)	Poland	Carbohydrases	ns	2.6	3.2
Thacker (2005), Exp. 1	Canada	Carbohydrases	ns	0.0	1.1
Thacker (2005), Exp. 2	Canada	Carbohydrases	ns	-1.9	0.0
Thacker and Rossnagel (2005)	Canada	Carbohydrases[5]	ns	3.9	3.1
			ns	-1.9	-3.7
			ns	-1.9	0.0
Thacker and Rossnagel (2005)	Canada	Carbohydrases[5]	ns	1.9	1.6
			ns	1.8	2.9
			ns	1.8	0.4
Kim et al. (2006)	USA	Carbohydrases[6]	G:F[3]	14.8	7.7
			G:F[3]	0.8	0.0
Roşu and Falcă (2007)	Romania	Carbohydrases	ns	6.5	9.4
Świątkiewicz and Hanczakowska (2008)	Poland	Carbohydrases[5]	ns	1.7	n/a
			ns	4.6	n/a
Wang et al. (2009)		Carbohydrases[a]	ADG, G:F	9.6	14.3

	South Korea	Carbohydrases[b]	ADG, G:F	8.4	16.5
Widyaratne et al. (2009)	Canada	Carbohydrases[5]	ns	-2.9	2.8
			ns	-1.1	-9.7
Jacela et al. (2010), Exp. 1	USA	Carbohydrases	ns	0.5	-0.6
Jacela et al. (2010), Exp. 4	USA	Carbohydrases	ns	-1.0	0.0
Yoon et al. (2010), Exp. 1[4]	South Korea	Carbohydrases	ADG[2]	0.4<br>4.7<br>2.6	1.1<br>5.2<br>2.1
Yoon et al. (2010), Exp. 2	South Korea	Carbohydrases[5]	ADG	4.1	8.5
			ADG	2.5	1.5
Barnes et al. (2011)	USA	Carbohydrases[5]	ADG	-0.9	1.7
			ADG	-1.4	-1.4
			ADG	-1.3	-1.0
Hanczakowska et al. (2012)	Poland	Carbohydrases[4]	ns	1.5	1.9
			ADG	4.2	3.2
			ADG, G:F	6.2	10.6
Jo et al. (2012), Exp. 1	South Korea	Carbohydrases[a]	ns	1.5	1.5
		Carbohydrases[b]	ADG	2.8	3.0
McAlpine et al. (2012)	Ireland	Carbohydrases	ADG	-7.2	n/a
Cho and Kim (2013)	South Korea	Carbohydrases[a]	ns	9.9	2.4
		Carbohydrases[b]	ADG, G:F	14.7	11.9
Kerr et al. (2013)	USA	Carbohydrases[a]	ns	5.2	-2.4
		Carbohydrases[b]	ns	-6.6	-12.3
		Carbohydrases[c]	ns	-2.0	-4.5
		Carbohydrases[d]	ns	-9.3	-9.3
		Carbohydrases[e]	ns	-2.4	-3.6
Kim et al. (2013)	South Korea	Carbohydrases[a]	ADG	10.3	8.2
		Carbohydrases[b]	ns	3.8	1.9
Lipiński et al. (2013)	Poland	Carbohydrases[4]	ADG	2.6	2.1
			ADG	3.0	2.9
O'Shea et al. (2014)	Ireland	Carbohydrases	ns	-7.3	-4.9
Villca et al. (2016)	Switzerland	Carbohydrases	ns	1.5	2.5
Lindemann (2016)	USA	Carbohydrases	ns	1.0	2.3
Nguyen et al. (2018)	South Korea	Carbohydrases	ADG[3], G:F	3.4	2.7
Torres-Pitarch et al. (2018)	Ireland	Carbohydrases	ns	0.3	0.0
Smit et al. (2019)	Canada	Carbohydrases	ADG3	2.0	2.4
Jang et al. (2020)	South Korea	Carbohydrases	ns	3.2	6.8
Jang et al. (2020)	South Korea	Carbohydrases	ns	1.7	-0.3
Torres-Pitarch et al. (2020)	Ireland	Carbohydrases[5]	G:F	3.1	5.2
			G:F	-1.2	2.3
Torres-Pitarch et al. (2020)	Ireland	Carbohydrases[5]	ns	-1.9	0.4
			ns	2.3	-1.1
Kpogo et al. (2021)	Canada	Carbohydrases	ns	-0.9	-2.6
O'Doherty and Forde (1999)	Ireland	Proteases	ns	0.5	2.4
Thacker (2005), Exp. 2	Canada	Proteases	ns	-3.9	-0.4
Reyna et al. (2006)[4]	Mexico	Proteases	ns	-8.2	-4.3
			ns	-4.2	-4.3
			ns	-1.5	-2.0
McAlpine et al. (2012)	Ireland	Proteases	ADG	-5.4	n/a
O'Shea et al. (2014)	Ireland	Proteases	ADG	-9.8	0.8

Stephenson et al. (2014)	USA	Proteases	ADG[3]	1.7	-0.7
Upadhaya et al. (2016)	South Korea	Proteases	G:F	3.2	2.6
Choe et al. (2017)	South Korea	Proteases	ADG, G:F	6.0	15.1
Lei et al. (2017)	South Korea	Proteases	G:F	2.2	6.5
Nguyen et al. (2018)	South Korea	Proteases[a]	ns	2.4	2.9
		Proteases[b]	ns	1.7	2.1
Figueroa et al. (2019)	Mexico	Proteases	ns	2.9	-2.2
Liu et al. (2019)[4]	South Korea	Proteases	ADG[2,3], G:F[2]	1.8<br>5.5<br>3.9	1.0<br>3.1<br>1.5
Min et al. (2019)	South Korea	Proteases	ADG	5.2	2.8
Lee et al. (2020)	South Korea	Proteases	G:F[3]	4.3	7.6
Kim et al. (2021)	South Korea	Proteases[a]	ns	1.3	0.0
		Proteases[b]	ADG, G:F	4.4	4.9
Perez-Palencia et al. (2021)	USA	Proteases	ns	-0.7	-0.2
Cromwell et al. (1993), Exp. 1	USA	Phytases	ns	4.6	6.0
Cromwell et al. (1993), Exp. 2	USA	Phytases	ns	1.1	-0.3
Cromwell et al. (1995)	USA	Phytases	ns	1.6	-2.1
Helander and Partanen (1997)	Finland	Phytases	ns	-1.7	-4.5
O'Doherty et al. (1999)	Ireland	Phytases[4]	ns	1.3	0.0
			ns	1.8	2.0
Gebert et al. (1999)	Switzerland	Phytases	ADG, G:F	10.6	8.2
Gagné et al. (2002)	Canada	Phytases	ns	-4.6	1.9
Brady et al. (2002)	Ireland	Phytases	ns	5.6	1.5
Thacker and Rossnagel (2006)	Canada	Phytases	ns	2.5	-3.2
Thacker et al. (2006)	Canada	Phytases	ns	5.2	3.3
Varley et al. (2010), Exp. 1	Ireland	Phytases	ns	4.2	1.6
Varley et al. (2010), Exp. 2	Ireland	Phytases	ns	-3.0	0.0
Kerr et al. (2013)	USA	Phytases	ns	-3.8	-6.6
Langbein et al. (2013)	USA	Phytases [a]	ns	-3.3	0.3
		Phytases [b]	ns	-2.3	-0.3
		Phytases [c]	ns	-2.3	-0.3
Patience (2015)	USA	Phytases[4]	ns	1.6	0.0
			ns	0.0	0.0
			ns	1.6	0.0
Pérez Alvarado et al. (2015)	Mexico	Phytases [a]	ns	0.0	6.9
		Phytases [b]	ns	-3.0	2.3
Lindemann (2016)	USA	Phytases[4]	ADG[2], G:F[2,3]	7.5<br>8.8<br>5.5	2.9<br>5.0<br>5.9
Holloway et al. (2019)	USA	Phytases[4]	ns	1.5	0.9
			ns	0.1	1.2
			ns	1.3	1.2
Dang and Kim (2021)	South Korea	Phytases	ADG, G:F	5.7	3.3
Dang and Kim (2021)	South Korea	Phytases	ADG, G:F[3]	4.1	2.9
Baas and Thacker (1996)	Canada	Multi-enzymes	ns	-2.3	2.4
Thacker (2005), Exp. 2	Canada	Multi-enzymes	ns	-2.9	0.4
Domaćinović et al. (2006)	Croatia	Multi-enzymes[5]	ns	0.7	0.3

			ns	1.6	1.3
Feoli et al. (2008)	USA	Multi-enzymes	ns	-0.3	-2.1
Benz et al. (2009)	USA	Multi-enzymes	ns	0.5	1.9
Thacker (2009)	Canada	Multi-enzymes	ns	5.7	2.8
Thacker and Haq (2009)	Canada	Multi-enzymes	ns	-1.2	0.7
Jacela et al. (2010), Exp. 2	USA	Multi-enzymes	ns	-0.1	-0.8
Jacela et al. (2010), Exp. 3	USA	Multi-enzymes[a]	ns	1.7	0.0
		Multi-enzymes[b]	ns	0.1	0.0
Ao et al. (2011)	South Korea	Multi-enzymes[4]	ADG[2], G:F[2]	6.3<br>7.6	2.8<br>5.6
Lee et al. (2011)	South Korea	Multi-enzymes	G:F	2.3	9.2
Jo et al. (2012), Exp. 1	South Korea	Multi-enzymes[a]	ADG	3.2	3.4
		Multi-enzymes[b]	ADG, G:F	5.0	4.9
Jo et al. (2012), Exp. 2	South Korea	Multi-enzymes	ADG, G:F	2.9	3.6
Kerr et al. (2013)	USA	Multi-enzymes	ns	-1.6	-6.6
Sitanaka et al. (2018)	Brazil	Multi-enzymes	ns	8.3	0.8
			ADG, G:F	24.9	11.2
			ns	0.8	3.8
			ns	7.2	1.3
Lawlor et al. (2019)	Ireland	Multi-enzymes	ADG, G:F	11.3	2.6
Balasubramanian et al. (2020)	South Korea	Multi-enzymes	ADG, G:F	8.5	16.9
Coelho et al. (2020)	Portugal	Multi-enzymes[a]	ns	-6.5	-5.5
		Multi-enzymes[b]	ns	-3.7	-1.5
Jerez-Bogota et al. (2020)	USA	Multi-enzymes[5]	G:F	0.0	30.8
			ADG	5.6	2.4
Huang et al. (2021)	China	Multi-enzymes	G:F	4.0	2.6

[1]Significant level at $P \leq 0.05$. Difference is calculated as [(treatment value – control value) / control value] * 100%.

[2]Polynomial contrasts were used for statistical analysis.

[3]Significant level at $0.05 < P \leq 0.10$.

[4]Same enzyme of each experiment was used with different inclusion levels. The inclusion level of each comparison increases from top to bottom.

[5]For experiments using factorial treatment structures, if the interaction of factors of either interested variable
was observed the effect of the feed additive within each level of the other factor is included within the database.

[6]The top comparison was the result of barrow and the bottom comparison was the results of gilts.

[a,b,c,d] Enzyme compositions within an experiment with different superscripts differ.

3.2. *Carcass Characteristics - Carbohydrases*

Back-fat significantly increased ($P \leq 0.05$) in 2 comparisons (average of 4.1%) and tended to increase ($0.05 < P \leq 0.10$) in 1 comparison (4.8%) compared to control pigs (Table S22). The

greatest proportion of the comparisons found no evidence of difference ($P > 0.10$) in BF (54 comparisons). Of these, BF was numerically increased ($P > 0.10$) in 18 comparisons (average of 4.0%) and numerically decreased ($P > 0.10$) in 29 comparisons (average of 3.7%) compared to control pigs. Percentage lean significantly increased ($P \leq 0.05$) in 1 comparison (5.6%) and significantly decreased ($P \leq 0.05$) in 2 comparisons (average of 0.7%) compared to control pigs. The greatest proportion of the comparisons found no evidence of difference ($P > 0.10$) in percentage lean (52 comparisons). Of these, percentage lean was numerically increased ($P > 0.10$) in 28 comparisons (average of 1.1%) and numerically decreased ($P > 0.10$) in 20 comparisons (average of 0.8%) compared to control pigs. All studies found no evidence of difference ($P > 0.10$) in LMA/LD (38 comparisons). Of these, LMA/LD was numerically increased ($P > 0.10$) in 20 comparisons (average of 3.3%) and numerically decreased ($P > 0.10$) in 17 comparisons (average of 1.5%) compared to control pigs.

Table S22. Studies on the effects of enzymes on carcass characteristics.

Author	Country	Enzyme	Sig.[1]	Difference, %[1]			
				Yield	BF	percentage lean	LMA/LD
Thacker et al. (1992)	Canada	Carbohydrases[5]	ns	0.9	n/a	-1.0	n/a
			ns	-0.3	n/a	-0.2	n/a
Kim et al. (1998)	USA	Carbohydrases	ns	0.6	-3.1	0.9	n/a
O'Doherty and Forde (1999)	Ireland	Carbohydrases	ns	-0.6	2.7	-0.5	0.1
Thacker and Campbell (1999)	Canada	Carbohydrases[5]	ns	1.7	0.5	0.2	-0.7
			ns	-0.4	-1.4	0.0	-2.7
Mavromichalis et al. (2000), Exp.1	USA	Carbohydrases	ns	0.1	-2.2	0.2	n/a
Mavromichalis et al. (2000), Exp.2	USA	Carbohydrases	ns	-0.1	-1.2	0.1	n/a
Grandhi (2001)	Canada	Carbohydrases	ns	0.1	-0.7	n/a	n/a
Pettey et al. (2002)	USA	Carbohydrases	ns	n/a	-4.9	1.9	5.9
Park et al. (2003), Exp.1	USA	Carbohydrases	ns	-0.3	0.4	-0.4	n/a
Park et al. (2003), Exp.2	USA	Carbohydrases[4]	ns[2]	2.6	-5.1	1.5	n/a
				0.4	-1.6	0.4	n/a
				0.7	-3.5	0.9	n/a
Park et al. (2003), Exp.3	USA	Carbohydrases[4]	ns[2]	-0.5	-1.6	0.2	n/a
				-2.9	-4.4	0.6	n/a
				0.0	-2.7	0.6	n/a
Park et al. (2003)	USA	Carbohydrases[5]	ns	-1.2	2.1	-0.2	n/a
			ns	-0.1	-6.9	1.6	n/a
			ns	-0.1	-7.6	2.0	n/a
			ns	-0.3	2.8	-0.4	n/a
			ns	-1.2	0.0	0.0	n/a
Flis and Sobotka (2005)	Poland	Carbohydrases	ns	n/a	n/a	-0.1	n/a
Thacker (2005), Exp. 2	Canada	Carbohydrases	ns	0.0	-5.3	0.7	-3.1
Thacker and Rossnagel (2005)	Canada	Carbohydrases[5]	ns	0.9	0.0	-0.3	-3.5
			ns	0.9	-1.6	0.3	3.1
			ns	-0.1	-2.1	0.0	1.6

Thacker and Rossnagel (2005)	Canada	Carbohydrases[5]	ns	0.5	-1.2	0.0	1.0
			ns	0.1	-5.9	0.8	3.2
			ns	0.9	-1.8	-0.2	-2.8
Świątkiewicz and Hanczakowska (2008)	Poland	Carbohydrases[5]	ns	8.1	-2.5	0.9	n/a
			ns	8.3	-1.9	-0.7	n/a
Wang et al. (2009)	South Korea	Carbohydrases[a]	ns	n/a	0.8	0.7	6.5
		Carbohydrases[b]	ns	n/a	2.5	1.1	9.8
Yoon et al. (2010), Exp. 1[4]	South Korea	Carbohydrases	ns[2]	1.0	-0.6	0.8	1.1
				0.2	-2.5	1.8	2.3
				0.3	-1.9	1.2	1.4
Yoon et al. (2010), Exp. 2	South Korea	Carbohydrases[5]	ns	4.0	16.1	-4.8	-2.6
			ns	1.8	6.7	8.1	12.7
Barnes et al. (2011)	USA	Carbohydrases[5]	ns	0.8	0.0	0.2	0.0
			ns	-1.5	-1.2	-0.4	-3.0
			ns	0.0	0.0	0.8	2.6
Pauly et al. (2011)	Ireland	Carbohydrases[5]	ns	-1.8	6.0	-1.0	n/a
			percentage lean	5.1	-17.0	5.6	n/a
Hanczakowska et al. (2012)	Poland	Carbohydrases[4]	ns	-0.2	-6.0	-0.4	-0.3
			ns	-0.3	0.0	-0.7	-0.3
			ns	0.2	0.5	0.6	0.1
Cho and Kim (2013)	South Korea	Carbohydrases[a]	ns	n/a	2.1	n/a	4.1
		Carbohydrases[b]	ns	n/a	3.4	n/a	5.1
O'Shea et al. (2014)	Ireland	Carbohydrases	ns	-0.4	-6.3	1.5	n/a
Lindemann (2016)	USA	Carbohydrases	ns	n/a	0.1	-0.1	-1.1
Villca et al. (2016)	Switzerland	Carbohydrases	ns	-0.1	-0.7	-0.2	-0.9
Nguyen et al. (2018)	South Korea	Carbohydrases	ns	n/a	n/a	n/a	0.1
Torres-Pitarch et al. (2018)	Ireland	Carbohydrases	ns	0.0	0.0	0.5	-0.2
Smit et al. (2019)	Canada	Carbohydrases	ns	0.3	0.0	0.2	0.8

Jang et al. (2020)	South Korea	Carbohydrases	ns	-0.2	-5.5	n/a	n/a
Torres-Pitarch et al. (2020)	Ireland	Carbohydrases[5]	ns	-0.5	6.2	-0.5	3.2
			ns	0.4	1.7	-0.3	-0.6
Torres-Pitarch et al. (2020)	Ireland	Carbohydrases[5]	BF, percentage lean	0.3	1.6	-0.2	-1.2
			BF, percentage lean	0.0	6.6	-1.2	-0.8
Kpogo et al. (2021)	Canada	Carbohydrases	BF[3]	0.2	4.8	n/a	-1.0
O'Doherty and Forde (1999)	Ireland	Proteases	ns	0.2	10.8	-2.1	-4.0
Thacker (2005), Exp. 2	Canada	Proteases	ns	0.4	-8.3	1.5	5.4
Reyna et al. (2006)	Mexico	Proteases[4]	ns	n/a	n/a	-1.2	-6.1
			ns	n/a	n/a	-1.1	-5.6
			ns	n/a	n/a	-1.9	-6.5
O'Shea et al. (2014)	Ireland	Proteases	ns	-0.4	-4.0	0.4	n/a
Stephenson et al. (2014)	USA	Proteases	Yield	-1.2	0.0	0.0	0.8
Choe et al. (2017)	South Korea	Proteases	Yield[3]	0.2	-2.7	-0.2	n/a
Torres-Pitarch et al. (2018)	Ireland	Proteases	ns	-0.7	0.8	-0.3	-2.0
Figueroa et al. (2019)	Mexico	Proteases	ns	n/a	1.9	n/a	-0.7
Liu et al. (2019)	South Korea	Proteases[4]	BF[2,3]	n/a<br>n/a<br>n/a	1.1<br>6.2<br>3.7	0.7<br>2.4<br>0.9	n/a<br>n/a<br>n/a
Min et al. (2019)	South Korea	Proteases	ns	-0.4	-5.4	n/a	n/a
Lee et al. (2020)	South Korea	Proteases	ns	-0.1	-4.2	n/a	n/a
Perez-Palencia et al. (2021)	USA	Proteases	ns	n/a	-1.7	0.4	1.1
Helander and Partanen (1997)	Finland	Phytases	ns	-1.7	n/a	n/a	n/a
O'Doherty et al. (1999)4	Ireland	Phytases[4]	ns	1.1	-6.5	1.3	0.9
			ns	3.1	0.0	-0.2	3.5
Brady et al. (2002)	Ireland	Phytases	Yield	-1.0	2.0	-0.6	n/a
Thacker et al. (2006)	Canada	Phytases	ns	0.0	-6.7	0.0	-11.6
Thacker and Rossnagel (2006)	Canada	Phytases	ns	-0.1	2.7	-0.8	-11.3
Varley et al. (2010), Exp. 1	Ireland	Phytases	ns	0.7	-0.8	-0.2	n/a

Varley et al. (2010), Exp. 2	Ireland	Phytases	ns	-0.7	1.6	-0.5	n/a
Langbein et al. (2013)	USA	Phytases [a]	ns	0.5	1.3	0.5	3.3
		Phytases [b]	ns	0.3	0.0	0.3	2.1
		Phytases [c]	ns	0.3	1.3	0.1	1.7
Pérez Alvarado et al. (2015)	Mexico	Phytases [a]	ns	n/a	-2.6	n/a	-1.8
		Phytases [b]	ns	n/a	-4.4	n/a	-3.0
Lindemann (2016)	USA	Phytases[4]	BF[2,3], percentage lean[2]	n/a	-6.1	1.4	2.3
				n/a	-13.4	2.9	4.0
				n/a	-10.8	2.8	6.4
Holloway et al. (2019)	USA	Phytases[4]	ns	0.3	n/a	n/a	n/a
			ns	0.0	n/a	n/a	n/a
			ns	-0.4	n/a	n/a	n/a
Dang and Kim (2021)	South Korea	Phytases	BF	n/a	8.3	n/a	0.4
Dang and Kim (2021)	South Korea	Phytases	ns	n/a	1.2	n/a	0.3
Thacker (2005), Exp. 2	Canada	Multi-enzymes	ns	0.8	-1.0	0.2	-1.4
Domaćinović et al. (2006)	Croatia	Multi-enzymes[5]	ns	n/a	n/a	1.1	n/a
			percentage lean	n/a	n/a	4.4	n/a
Feoli et al. (2008)	USA	Multi-enzymes	ns	0.1	-1.6	0.0	-1.1
Benz et al. (2009)	USA	Multi-enzymes	ns	-0.4	-0.5	-0.4	-2.2
Thacker (2009)	Canada	Multi-enzymes	ns	-1.9	-3.9	0.2	-3.2
Thacker and Haq (2009)	Canada	Multi-enzymes	ns	0.1	11.3	-1.3	3.5
Lee et al. (2011)	South Korea	Multi-enzymes	ns	0.2	-12.3	n/a	n/a
Ao et al. (2011)	South Korea	Multi-enzymes[4]	ns[2]	n/a	1.9	0.7	0.3
				n/a	1.4	1.2	1.7
Balasubramanian et al. (2020)	South Korea	Multi-enzymes	BF	n/a	-10.2	n/a	-6.0
Coelho et al. (2020)	Portugal	Multi-enzymes[a]	ns	-0.3	29.4	n/a	n/a
		Multi-enzymes[b]	ns	-0.4	15.5	n/a	n/a

Huang et al. (2021)	China	Multi-enzymes	LMA	1.7	3.1	n/a	11.3

[1]Significant level at $P \leq 0.05$. Difference is calculated as [(treatment value – control value) / control value] * 100%.

[2]Polynomial contrasts were used for statistical analysis.

[3]Significant level at $0.05 < P \leq 0.10$.

[4]Same enzyme of each experiment was used with different inclusion levels. The inclusion level of each comparison increases from top to bottom.

[5]For experiments using factorial treatment structures, if the interaction of factors of either interested variable was observed the effect of the feed additive within each level of the other factor is included within the database.

[a,b,c] Enzyme compositions within an experiment with different superscripts differ.

3.3. *Growth performance - Proteases*

Average daily gain significantly increased ($P \leq 0.05$) in 3 comparisons (average of 5.2%), tended to increase ($0.05 < P \leq 0.10$) in 4 comparisons (average of 3.2%), and significantly decreased ($P \leq 0.05$) in 2 comparisons (average of 7.6%) compared to control pigs (Table S21). The greatest proportion of the comparisons found no evidence of difference ($P > 0.10$) in ADG (14 comparisons). Of these, ADG was numerically increased ($P > 0.10$) in 9 comparisons (average of 2.1%) and numerically decreased ($P > 0.10$) in 5 comparisons (average of 3.7%) compared to control pigs. Feed efficiency significantly increased ($P \leq 0.05$) in 7 comparisons (average of 4.9%) and tended to increase ($0.05 < P \leq 0.10$) in 1 comparison (7.6%) compared to control pigs. The greatest proportion of the comparisons found no evidence of difference ($P > 0.10$) in G:F (14 comparisons). Of these, G:F was numerically increased ($P > 0.10$) in 5 comparisons (average of 2.2%) and numerically decreased ($P > 0.10$) in 7 comparisons (average of 2%) compared to control pigs. Overall, the results suggest that proteases had positive effects on ADG and G:F (70 and 59% of all the comparisons), but the effects were small for ADG. Moreover, in a meta-analysis conducted by Aranda-Aguirre et al. [302], the authors found that proteases had no effects ($P > 0.10$) on growth performance of finishing pigs. There were not enough data to support whether different basal diets affected the response to proteases for ADG and G:F. The lack of substantial positive effects of exogenous proteases may be due to the high digestibility of dietary protein with the endogenous proteases of the mature grow-

finish pig. Even though the digestibility of CP or N was improved ($P \leq 0.05$) in some studies [332, 345, 352, 372], the improvements may not be large enough to improve growth performance.

3.4. *Carcass Characteristics - Proteases*

Back-fat tended to increase ($0.05 < P \leq 0.10$) in 3 comparisons (average of 3.7%) compared to control pigs (Table S22). The greatest proportion of the comparisons found no evidence of difference ($P > 0.10$) in BF (10 comparisons). Of these, BF was numerically increased ($P > 0.10$) in 3 comparisons (average of 4.5%) and numerically decreased ($P > 0.10$) in 6 comparisons (average of 4.4%) compared to control pigs. All the comparisons found no evidence of difference ($P > 0.10$) in percentage lean. Of these, percentage lean was numerically increased ($P > 0.10$) in 6 comparisons (average of 1.1%) and numerically decreased ($P > 0.10$) in 6 comparisons (average of 1.1%) compared to control pigs. All the comparisons found no evidence of difference ($P > 0.10$) in LMA/LD. Of these, LMA/LD was numerically increased ($P > 0.10$) in 3 comparisons (average of 2.4%) and numerically decreased ($P > 0.10$) in 6 comparisons (average of 4.1%) compared to control pigs.

3.5. *Growth performance - Phytases*

Average daily gain significantly increased ($P \leq 0.05$) in 3 comparisons (average of 6.8%) compared to control pigs (Table S21). The greatest proportion of the comparisons found no evidence of difference ($P > 0.10$) in ADG (21 comparisons). Of these, ADG was numerically

increased ($P > 0.10$) in 12 comparisons (average of 2.6%) and numerically decreased ($P > 0.10$) in 8 comparisons (average of 3.0%) compared to control pigs. Feed efficiency significantly increased ($P \leq 0.05$) in 2 comparisons (average of 5.7%) and tended to increase ($0.05 < P \leq 0.10$) in 1 comparison (2.9%) compared to control pigs. The greatest proportion of the comparisons found no evidence of difference ($P > 0.10$) in G:F (21 comparisons). Of these, G:F was numerically increased ($P > 0.10$) in 13 comparisons (average of 2.3%) and numerically decreased ($P > 0.10$) in 7 comparisons (average of 2.5%) compared to control pigs. Overall, the results suggest that phytases had positive effects on ADG (63% of all comparisons) and G:F (67% of all comparisons), but most comparisons were not statistically significant (88% of all comparisons). Moreover, in a meta-analysis conducted by Aranda-Aguirre et al. [302], the authors found that phytases had no effects ($P > 0.10$) on growth performance of finishing pigs. There was not enough data to support whether different phytase inclusion levels and basal diets affected the response to phytases for ADG and G:F in grow-finish pig diets with adequate P levels.

3.6. *Carcass Characteristics - Phytases*

Back-fat significantly increased ($P \leq 0.05$) in 1 comparison (8.3%) compared to control pigs (Table S22). The greatest proportion of the comparisons found no evidence of difference ($P > 0.10$) in BF (13 comparisons). Of these, BF was numerically increased ($P > 0.10$) in 6 comparisons (average of 1.7%) and numerically decreased ($P > 0.10$) in 5 comparisons (average

of 4.2%) compared to control pigs. All the comparisons found no evidence of difference (*P* > 0.10) in percentage lean. Of these, percentage lean was numerically increased (*P* > 0.10) in 4 comparisons (average of 0.6%) and numerically decreased (*P* > 0.10) in 5 comparisons (average of 0.5%) compared to control pigs. All the comparisons found no evidence of difference (*P* > 0.10) in LMA/LD. Of these, LMA/LD was numerically increased (*P* > 0.10) in 7 comparisons (average of 1.7%) and numerically decreased (*P* > 0.10) in 4 comparisons (average of 6.9%) compared to control pigs.

3.7. *Growth performance - Multi-enzymes*

Average daily gain significantly increased (*P* ≤ 0.05) in 10 comparisons (average of 7.9%) compared to control pigs (Table S21). The greatest proportion of the comparisons found no evidence of difference (*P* > 0.10) in ADG (19 comparisons). Of these, ADG was numerically increased (*P* > 0.10) in 10 comparisons (average of 2.9%) and numerically decreased (*P* > 0.10) in 8 comparisons (average of 2.3%) compared to control pigs. Feed efficiency significantly increased (*P* ≤ 0.05) in 10 comparisons (average of 9.0%) compared to control pigs. The greatest proportion of the comparisons found no evidence of difference (*P* > 0.10) in G:F (19 comparisons). Of these, G:F was numerically increased (*P* > 0.10) in 12 comparisons (average of 1.8%) and numerically decreased (*P* > 0.10) in 5 comparisons (average of 3.3%) compared to control pigs. Overall, the results suggest that multi-enzymes have positive effects on ADG and G:F (69 and 76% of all the comparisons), and multi-enzymes significantly improved (*P* ≤ 0.05)

ADG and G:F in 34% of all the comparisons. Moreover, the combination of multiple enzymes provided greater improvement than adding any single type of enzyme (carbohydrase, protease, and phytase) alone, which suggests that different types of enzymes may have a synergetic effect. However, most comparisons showed little or negative effects in US-based research; therefore, the utilization of multi-enzymes in US-based diets should be evaluated further. There are not enough data to support whether different basal diets affected the response to multi-enzymes for ADG or G:F. Nevertheless, similar to the results with carbohydrases, multi-enzymes improved pig performance when diets were marginal in nutrient concentrations. In summary, there was a low chance of negative effects by feeding multi-enzymes and they can potentially improve growth performance (approximately 3% improvement for ADG and G:F).

3.8. *Carcass Characteristics - Multi-enzymes*

Back-fat significantly decreased ($P \leq 0.05$) in 1 comparison (10.2%) compared to control pigs (Table S22). The greatest proportion of the comparisons found no evidence of difference ($P > 0.10$) in BF (11 comparisons). Of these, BF was numerically increased ($P > 0.10$) in 6 comparisons (average of 10.4%) and numerically decreased ($P > 0.10$) in 5 comparisons (average of 3.8%) compared to control pigs. Percentage lean significantly increased ($P \leq 0.05$) in 1 comparison (4.4%) compared to control pigs. The greatest proportion of the comparisons found no evidence of difference ($P > 0.10$) in percentage lean (8 comparisons). Of these,

percentage lean was numerically increased ($P > 0.10$) in 6 comparisons (average of 0.6%) and numerically decreased ($P > 0.10$) in 2 comparisons (average of 0.9%) compared to control pigs. Loin muscle area/depth significantly increased ($P \leq 0.05$) in 1 comparison (11.3%) compared to control pigs. The greatest proportion of the comparisons found no evidence of difference ($P > 0.10$) in LMA/LD (7 comparisons). Of these, LMA/LD was numerically increased ($P > 0.10$) in 3 comparisons (average of 1.8%) and numerically decreased ($P > 0.10$) in 5 comparisons (average of 2.8%) compared to control pigs.

4. Conclusion

In conclusion, this literature review collected available research on finishing pig feed additives to provide a descriptive analysis of the effects on growth and carcass performance and provides a database that can be further analyzed with advanced statistical methods, such as meta-analysis, in the hope of better understanding the effect of feed additives to improve the efficiency of swine production.

Author Contributions: All authors participated in conceptualization, writing, and review and editing of the manuscript. All authors have read and agreed to the published version of the manuscript.

Funding: This article, wholly or in part, was funded by the National Pork Board.

Institutional Review Board Statement: Not applicable

Informed Consent Statement: Not applicable

Data Availability Statement: Not applicable

Acknowledgments: Contribution no. 23-016-J from the Kansas Agric. Exp. Stn., Manhattan KS, 66506-0210 USA.

Conflict of Interest: The authors declare no conflict of interest.

References

1. Thacker, P.A.; J.P. Bowland. Influence of Graded Levels of Dietary Propionic Acid on Performance and Carcass Traits of Swine Fed Diets Supplemented with Soybean Meal or Canola Meal. *Can. J. Anim. Sci.* **1980**, 60(4), 971-978. doi:10.4141/cjas80-115
2. Thacker, P.A.; J.P. Bowland. Effects of Vitamin B12 on Performance and Carcass Traits of Pigs Fed Diets Supplemented with Propionic Acid or Calcium Propionate. *Can. J. Anim. Sci.* **1981**, 61(3), 775-782. doi:10.4141/cjas81-094
3. Thacker, P.A.; M.O. Salomons; F.X. Aherne; L.P. Milligan; J.P. Bowland. Influence of Propionic Acid on the Cholesterol Metabolism of Pigs Fed Hypercholesterolemic Diets. *Can. J. Anim. Sci.* **1981**, 61(4), 969-975. doi:10.4141/cjas81-119
4. Giesting, D.W.; R.A. Easter. Response of Starter Pigs to Supplementation of Corn-Soybean Meal Diets with Organic Acids. *J. Anim. Sci.* **1985**, 60(5), 1288-1294. doi:10.2527/jas1985.6051288x
5. Thacker, P.A.; G.L. Campbell; J. GrootWassink. The Effect of Organic Acids and Enzyme Supplementation on the Performance of Pigs Fed Barley-Based Diets. *Can. J. Anim. Sci.* **1992**, 72(2), 395-402. doi:10.4141/cjas92-047
6. Baustad, B. Effects of Formic Acid on Performance in Growing Pigs. *Norw. J. Agric. Sci.* **1993**, 7(1), 61-69.

7. Krause, D.O.; P.C. Harrison; R.A. Easter. Characterization of the Nutritional Interactions between Organic Acids and Inorganic Bases in the Pig and Chick. *J. Anim. Sci.* **1994**, 72(5), 1257-1262. doi:10.2527/1994.7251257x

8. Siljander-Rasi, H.; T. Alaviuhkola; K. Suomi. Carbadox, Formic Acid and Potato Fibre as Feed Additives for Growing Pigs. *J. Anim. Feed Sci.* **1998**, 7(Supp 1), 205-209. doi:10.22358/jafs/69977/1998

9. Partanen, K.; H. Siljader-Rasi; T. Alaviuhkola; K. Suomi; M. Fossi. Performance of Growing-Finishing Pigs Fed Medium- or High-Fibre Diets Supplemented with Avilamycin, Formic Acid or Formic Acid-Sorbate Blend. *Livest. Prod. Sci.* **2002**, 73(2/3), 139-152. doi:10.1016/S0301-6226(01)00255-X

10. Canibe, N.; O. Højberg; S. Højsgaard; B.B. Jensen. Feed Physical Form and Formic Acid Addition to the Feed Affect the Gastrointestinal Ecology and Growth Performance of Growing Pigs. *J. Anim. Sci.* **2005**, 83(6), 1287-1302. doi:10.2527/2005.8361287x

11. Jansons, I.; J. Nudiens. Acidifiers Additive Projection on Pigs Metabolic Processes and Digestive Tract Microflora. *Res. Rural Dev.* **2005**, 23-25.

12. Bühler, K.; C. Wenk; J. Broz; S. Gebert. Influence of Benzoic Acid and Dietary Protein Level on Performance, Nitrogen Metabolism and Urinary Ph in Growing-Finishing Pigs. *Arch. Anim. Nutr.* **2006**, 60(5), 382-9. doi:10.1080/17450390600884369

13. Campbell, A.J.; G.E. Gardiner; F.C. Leonard; P.B. Lynch; C. Stanton; R.P. Ross; P.G. Lawlor. The Effect of Dietary Supplementation of Finishing Pigs with Organic Acids or Mannan-Oligosaccharide on the Coliform, Lactobacillus and Bifidobacterium Flora on the Intestinal Contents and Faeces. *Pig J.* **2006**, 57, 90-104.

14. Partanen, K.; M. Karhapää; H. Siljander-Rasi; E. Virtanen; B. Nilsson. Performance, Carcass Quality, and Gastric Alterations in Fattening Pigs Fed Additives Containing Formic Acid Either Coated with Sorbate or Mixed with Lactic Acid. *Agric. Food Sci.* **2006**, 15(3), 324-339. doi:10.2137/145960606779216308

15. Eisemann, J.H.; E.v. Heugten. Response of Pigs to Dietary Inclusion of Formic Acid and Ammonium Formate. *J. Anim. Sci.* **2007**, 85(6), 1530-1539. doi:10.2527/jas.2006-464

16. Øverland, M.; N.P. Kjos; M. Borg; H. Sørum. Organic Acids in Diets for Entire Male Pigs. *Livest. Sci.* **2007**, 109(1/3), 170-173. doi:10.1016/j.livsci.2007.02.002

17. Guy, J.H.; H.L. Edge; P.J. Blanchard; S.E. Ilsley; C. Coonan; D. Feuerstein. A Note on the Effect of Supplementation with Microbial Phytase and Organic Acids on Feed Intake and Growth Performance of Growing Pigs. *Irish J. Agric. Food Res.* **2008**, 47(1), 93-97.

18. Kijparkorn, S.; U. Jamikorn; S. Wangsoonean; P. Ittitanawong. Antioxidant and Acidifier Properties of Roselle (Hibicus Sabdariffa Linn.) Calyx Powder on Lipid Peroxidation, Nutrient Digestibility and Growth Performance in Fattening Pigs. *Thai. J. Vet. Med* **2009**, 39(1), 41-51.

19. Thacker, P.A.; I. Haq. Effect of Enzymes, Flavor and Organic Acids on Nutrient Digestibility, Performance and Carcass Traits of Growing-Finishing Pigs Fed Diets Containing Dehydrated Lucerne Meal. *J. Sci. Food Agric.* **2009**, 89(1), 101-108. doi:10.1002/jsfa.3415

20. Jansons, I.; A. Jemeljanovs; I.H. Konosonoka; V. Sterna; B. Lujane. The Influence of Organic Acid Additive, Phytoadditive and Complex of Organic Acid Additive Phytoadditive on Pig Productivity, Meat Quality. *Agron. Res.* **2011**, 9(Special Issue II), 389-394.

21. Upadhaya, S.D.; K.Y. Lee; I.H. Kim. Protected Organic Acid Blends as an Alternative to Antibiotics in Finishing Pigs. *Asian Australas. J. Anim. Sci.* **2014**, 27(11), 1600-1607. doi:10.5713/ajas.2014.14356

22. Cho, J.H.; S.I. Lee; I.H. Kim. Effects of Different Levels of Fibre and Benzoic Acid on Growth Performance, Nutrient Digestibility, Reduction of Noxious Gases, Serum Metabolites and Meat Quality in Finishing Pigs. *J. Appl. Anim. Res.* **2015**, 43(3), 336-344. doi:10.1080/09712119.2014.978772

23. Giannenas, I.; D. Doukas; A. Karamoutsios; A. Tzora; E. Bonos; I. Skoufos; A. Tsinas; E. Christaki; D. Tontis; P. Florou-Paneri. Effects of Enterococcus Faecium, Mannan Oligosaccharide, Benzoic Acid and Their Mixture on Growth Performance, Intestinal Microbiota, Intestinal Morphology and Blood Lymphocyte Subpopulations of Fattening Pigs. *Anim. Feed Sci. Technol.* **2016**, 220, 159-167. doi:10.1016/j.anifeedsci.2016.08.003

24. Zhai, H.; W. Ren; S. Wang; J. Wu; P. Guggenbuhl; A.M. Kluenter. Growth Performance of Nursery and Grower-Finisher Pigs Fed Diets Supplemented with Benzoic Acid. *Anim. Nutr.* **2017**, 3(3), 232-235. doi:10.1016/j.aninu.2017.05.001

25. Lei, X.J.; S.I. Lee; K.Y. Lee; D.H. Nguyen; I.H. Kim. Effects of a Blend of Organic Acids and Medium-Chain Fatty Acids with and without Enterococcus Faecium on Growth Performance, Nutrient Digestibility, Blood Parameters, and Meat Quality in Finishing Pigs. *Can. J. Anim. Sci.* **2018**, 98(4), 852-859. doi:10.1139/cjas-2017-0126

26. Nguyen Thi, T. Probiotic and Organic Acids Improve Growth Performance of Pigs Fed Diets Containing Catfish (Pangasius Hypophthalmus) Protein Hydrolysate. *Livest. Res. Rural. Dev.* **2018**, 30(8), 143.

27. Morel, P.C.H.; K.L. Chidgey; C.M.C. Jenkinson; I. Lizarraga; N.M. Schreurs. Effect of Benzoic Acid, Sodium Butyrate and Sodium Butyrate Coated with Benzoic Acid on Growth Performance, Digestibility, Intestinal Morphology and Meat Quality in Grower-Finisher Pigs. *Livest. Sci.* **2019**, 226, 107-113. doi:10.1016/j.livsci.2019.06.009

28. Nguyen, D.H.; K.Y. Lee; H.N. Tran; I.H. Kim. Effect of a Protected Blend of Organic Acids and Medium-Chain Fatty Acids on Growth Performance, Nutrient Digestibility, Blood Profiles, Meat Quality, Faecal Microflora, and Faecal Gas Emission in Finishing Pigs. *Can. J. Anim. Sci.* **2019**, 99(3), 448-455. doi:10.1139/cjas-2016-0174

29. O' Meara, F.M.; G.E. Gardiner; J.V. O' Doherty; P.G. Lawlor. Effect of Dietary Inclusion of Benzoic Acid (Vevovitall®) on the Microbial Quality of Liquid Feed and the Growth and Carcass Quality of Grow-Finisher Pigs. *Livest. Sci.* **2020**, 237. doi:10.1016/j.livsci.2020.104043

30. Tran Thi Bich, N.; O. Duong Thi; L. Pineda; S. Ayudhya; N.d. Groot; Y. Han. The Effects of Synergistic Blend of Organic Acid or Antibiotic Growth Promoter on Performance and Antimicrobial Resistance of Bacteria in Grow-Finish Pigs. *Transl. Anim. Sci.* **2020**, 4(4). doi:10.1093/tas/txaa211

31. Muniyappan, M.; T. Palanisamy; I.H. Kim. Effect of Microencapsulated Organic Acids on Growth Performance, Nutrient Digestibility, Blood Profile, Fecal Gas Emission, Fecal Microbial, and Meat-Carcass Grade Quality of Growing-Finishing Pigs. *Livest. Sci.* **2021**, 252. doi:10.1016/j.livsci.2021.104658

32. Tutida, Y.H.; J.H. Montes; K.K. Borstnez; H.A. Siqueira; M.F. Güths; F. Moreira; V. Peripolli; R. Irgang; N. Morés; I. Bianchi; J.D. Kich. Effects of in Feed Removal of Antimicrobials in Comparison to Other Prophylactic Alternatives in Growing and Finishing Pigs. *Arq. Bras. Med. Vet. Zootec.* **2021**, 73(6), 1381-1390. doi:10.1590/1678-4162-12450

33. Onibala, J.S.I.T.; K.D. Gunther; U.t. Meulen. Effects of Essential Oil of Spices as Feed Additives on the Growth and Carcass Characteristics of Growing-Finishing Pigs. *Tropenlandwirt* **2001**(No.73), 179-184.

34. Yan, L.; J.P. Wang; H.J. Kim; Q.W. Meng; X. Ao; S.M. Hong; I.H. Kim. Influence of Essential Oil Supplementation and Diets with Different Nutrient Densities on Growth Performance, Nutrient Digestibility, Blood Characteristics, Meat Quality and Fecal Noxious Gas Content in Grower-Finisher Pigs. *Livest. Sci.* **2010**, 128(1/3), 115-122. doi:10.1016/j.livsci.2009.11.008

35. Simitzis, P.E.; G.K. Symeon; M.A. Charismiadou; J.A. Bizelis; S.G. Deligeorgis. The Effects of Dietary Oregano Oil Supplementation on Pig Meat Characteristics. *Meat Sci.* **2010**, 84(4), 670-676. doi:10.1016/j.meatsci.2009.11.001

36. Zhou, Y.; Z. Ruan; X.L. Li; S.M. Mi; M. Jiang; W.H. Liu; H.S. Yang; X. Wu; G.L. Jiang; Y.L. Yin. Eucommia Ulmoides Oliver Leaf Polyphenol Supplementation Improves Meat Quality and Regulates Myofiber Type in Finishing Pigs. *J. Anim. Sci.* **2016**, 94(Suppl. 3), 164-168. doi:10.2527/jas.2015-9551

37. Zou, Y.; Q. Xiang; J. Wang; H. Wei; J. Peng. Effects of Oregano Essential Oil or Quercetin Supplementation on Body Weight Loss, Carcass Characteristics, Meat Quality and Antioxidant Status in Finishing Pigs under Transport Stress. *Livest. Sci.* **2016**, 192, 33-38. doi:10.1016/j.livsci.2016.08.005

38. Li, T.S.; W.C. Liu; P.Y. Zhao; I.H. Kim. Evaluation of Essential Oil or/and Emulsifier in Low Energy Density Diets on Growth Performance, Nutrient Digestibility, Blood Cholesterol and Meat Quality in Finishing Pigs. *Ital. J. Anim. Sci.* **2017**, 16(4), 624-630. doi:10.1080/1828051X.2017.1325718

39. Soto, J.; M.D. Tokach; S.S. Dritz; J.C. Woodworth; J.M. DeRouchey; R.D. Goodband. Evaluation of Dietary Phytogenics on Growth Performance and Carcass Characteristics of Pigs During the Growing-Finishing Phase. *Kansas Agricultural Experiment Station Research Reports* **2017**, 3(7). doi:10.4148/2378-5977.7498

40. Zou, Y.; X. Hu; T. Zhang; H. Wei; Y. Zhou; Z. Zhou; J. Peng. Effects of Dietary Oregano Essential Oil and Vitamin E Supplementation on Meat Quality, Stress Response and Intestinal Morphology in Pigs Following Transport Stress. *J. Vet. Med. Sci.* **2017**, 79(2), 328-335. doi:10.1292/jvms.16-0576

41. Cheng, C.; M. Xia; X. Zhang; C. Wang; S. Jiang; J. Peng. Supplementing Oregano Essential Oil in a Reduced-Protein Diet Improves Growth Performance and Nutrient Digestibility by Modulating Intestinal Bacteria, Intestinal Morphology, and Antioxidative Capacity of Growing-Finishing Pigs. *Animals* **2018**, 8(9), 159. doi:10.3390/ani8090159

42. Lan, R.; I. Kim. Effects of Feeding Diets Containing Essential Oils and Betaine to Heat-Stressed Growing-Finishing Pigs. *Arch. Anim. Nutr.* **2018**, 72(5), 368-378. doi:10.1080/1745039x.2018.1492806

43. Lowell, J.E.; B.M. Bohrer; K.B. Wilson; M.F. Overholt; B.N. Harsh; H.H. Stein; A.C. Dilger; D.D. Boler. Growth Performance, Carcass Quality, Fresh Belly Characteristics, and Commercial Bacon Slicing Yields of Growing-Finishing Pigs Fed a Subtherapeutic Dose of an Antibiotic, a Natural Antimicrobial, or Not Fed an Antibiotic or Antimicrobial. *Meat Sci.* **2018**, 136, 93-103. doi:10.1016/j.meatsci.2017.10.011

44. Huang, C.; D. Chen; G. Tian; J. He; P. Zheng; J. Yu; X. Mao; Z. Huang; H. Yan; Q. Wang; H. Wang; B. Yu. Effects of Dietary Plant Essential Oil Supplementation on Growth Performance, Nutrient Digestibility and Meat Quality in Finishing Pigs. *J. Anim. Physiol. Anim. Nutr. (Berl.)* **2021**. doi:10.1111/jpn.13673

45. Cho, J.H.; M.H. Song; I.H. Kim. Effect of Microencapsulated Blends of Organic Acids and Essential Oils Supplementation on Growth Performance and Nutrient Digestibility in Finishing Pigs. *Rev. Colomb. Cienc. Pecu.* **2014**, 27(4), 264-272.

46. Walia, K.; H. Argüello; H. Lynch; J. Grant; G. Duffy. Effect of Strategic Administration of an Encapsulated Blend of Formic Acid, Citric Acid, and Essential Oils on Salmonella Carriage, Seroprevalence, and Growth of Finishing Pigs. *Prev. Vet. Med.* **2017**, 137, 28-35. doi:10.1016/j.prevetmed.2016.12.007

47. Oh, H.J.; I.H. Kim; M.H. Song; W.G. Kwak; W. Yun; J.H. Lee; C.H. Lee; S.Y. Oh; S. Liu; J.S. An; H.B. Kim; J.H. Cho. Effects of Microencapsulated Complex of Organic Acids and Essential Oils on Growth Performance, Nutrient Retention, Blood Profiles, Fecal Microflora, and Lean Meat Percentage in Weaning to Finishing Pigs. *Can. J. Anim. Sci.* **2019**, 99(1), 41-49. doi:10.1139/cjas-2018-0006

48. Resende, M.; R.F. Chaves; R.M. Garcia; J.A. Barbosa; A.S. Marques; L.R. Rezende; A.P. Peconick; C.A.P. Garbossa; D. Mesa; C.C. Silva; V.B. Fascina; F.T.F. Dias; V.d.S. Cantarelli. Benzoic Acid and Essential Oils Modify the Cecum Microbiota Composition in Weaned Piglets and Improve Growth Performance in Finishing Pigs. *Livest. Sci.* **2020**, 242. doi:10.1016/j.livsci.2020.104311

49. Hutchens, W.M.; M.D. Tokach; J.C. Woodworth; J.M. Derouchey; R.D. Goodband; J.T. Gebhardt; H.I. Calderon; K. Keppy; P. Maynard; E. Grilli. Evaluation of Microencapsulated Organic Acids and Botanicals on Growth Performance of Nursery and Growing-Finishing Pigs. *Transl. Anim. Sci.* **2021**, 5(4). doi:10.1093/tas/txab205

50. Zimmermann, J.A.; M.L. Fusari; E. Rossler; J.E. Blajman; A. Romero-Scharpen; D.M. Astesana; C.R. Olivero; A.P. Berisvil; M.L. Signorini; M.V. Zbrun; L.S. Frizzo; L.P. Soto. Effects of Probiotics in Swines Growth Performance: A

Meta-Analysis of Randomised Controlled Trials. *Anim. Feed Sci. Technol.* **2016**, 219, 280-293. doi:10.1016/j.anifeedsci.2016.06.021

51. Pollmann, D.S.; D.M. Danielson; E.R. Peo, Jr. Effects of Microbial Feed Additives on Performance of Starter and Growing-Finishing Pigs. *J. Anim. Sci.* **1980**, 51(3), 577-581. doi:10.2527/jas1980.513577x

52. Harper, A.F.; E.T. Kornegay; K.L. Bryant; H.R. Thomas. Efficacy of Virginiamycin and a Commercially-Available Lactobacillus Probiotic in Swine Diets. *Anim. Feed Sci. Technol.* **1983**, 8(1), 69-76. doi:10.1016/0377-8401(83)90044-5

53. Kim, I.H.; J.D. Hancock; R.H. Hines; C.S. Kim. Effects of Cellulase Enzymes and Bacterial Feed Additives on the Nutritional Value of Sorghum Grain for Finishing Pigs. *Asian Australas. J. Anim. Sci.* **1998**, 11(5), 538-544. doi:10.5713/ajas.1998.538

54. Kyriakis, S.C.; I. Georgoulakis; A. Spais; C. Alexopoulos; C.C. Miliotis; S.K. Kritas. Evaluation of Toyocerin, a Probiotic Containing Bacillus Toyoi Spores, on Health Status and Productivity of Weaned, Growing and Finishing Pigs. *Asian Australas. J. Anim. Sci.* **2003**, 16(9), 1326-1331. doi:10.5713/ajas.2003.1326

55. Alexopoulos, C.; I.E. Georgoulakis; A. Tzivara; C.S. Kyriakis; A. Govaris; S.C. Kyriakis. Field Evaluation of the Effect of a Probiotic-Containing Bacillus Licheniformis and Bacillus Subtilis Spores on the Health Status, Performance, and Carcass Quality of Grower and Finisher Pigs. *J. Vet. Med.* **2004**, 51(6), 306-312. doi:10.1111/j.1439-0442.2004.00637.x

56. Rekiel, A.; J. Więcek; M. Dziuba. Effect of Feed Additives on the Results of Fattening and Selected Slaughter and Quality Traits of Pork Meat of Pigs with Different Genotypes. *Czech J. Anim. Sci.* **2005**, 50(12), 561-567. doi:10.17221/4262-CJAS

57. Jukna, Č.; V. Jukna; A. Šimkus. The Effect of Probiotics and Phytobiotics on Meat Properties and Quality of Pigs. *Vet. ir Zootech.* **2005**, 29(51), 80-84.

58. Shon, K.S.; J.W. Hong; O.S. Kwon; B.J. Min; W.B. Lee; I.H. Kim; Y.H. Park; I.S. Lee. Effects of Lactobacillus Reuteri-Based Direct-Fed Microbial Supplementation for Growing-Finishing Pigs. *Asian Australas. J. Anim. Sci.* **2005**, 18(3), 370-374. doi:10.5713/ajas.2005.370

59. Chen, Y.J.; B.J. Min; J.H. Cho; O.S. Kwon; K.S. Son; H.J. Kim; I.H. Kim. Effects of Dietary Bacillus-Based Probiotic on Growth Performance, Nutrients Digestibility, Blood Characteristics and Fecal Noxious Gas Content in Finishing Pigs. *Asian Australas. J. Anim. Sci.* **2006**, 19(4), 587-592. doi:10.5713/ajas.2006.587

60. Chen, Y.J.; B.J. Min; J.H. Cho; O.S. Kwon; K.S. Son; I.H. Kim; S.J. Kim. Effects of Dietary Enterococcus Faecium Sf68 on Growth Performance, Nutrient Digestibility, Blood Characteristics and Faecal Noxious Gas Content in Finishing Pigs. *Asian Australas. J. Anim. Sci.* **2006**, 19(3), 406-411. doi:10.5713/ajas.2006.406

61. Davis, M.E.; T. Parrott; D.C. Brown; B.Z.d. Rodas; Z.B. Johnson; C.V. Maxwell; T. Rehberger. Effect of a Bacillus-Based Direct-Fed Microbial Feed Supplement on Growth Performance and Pen Cleaning Characteristics of Growing-Finishing Pigs. *J. Anim. Sci.* **2008**, 86(6), 1459-1467. doi:10.2527/jas.2007-0603

62. Ko, S.Y.; I.H. Bae; S.T. Yee; S.S. Lee; D. Uuganbayar; J.I. Oh; C.J. Yang. Comparison of the Effect of Green Tea by-Product and Green Tea Probiotics on the Growth Performance, Meat Quality, and Immune Response of Finishing Pigs. *Asian Australas. J. Anim. Sci.* **2008**, 21(10), 1486-1494. doi:10.5713/ajas.2008.70604

63. Ko, S.Y.; C.J. Yang. Effect of Green Tea Probiotics on the Growth Performance, Meat Quality and Immune Response in Finishing Pigs. *Asian Australas. J. Anim. Sci.* **2008**, 21(9), 1339-1347. doi:10.5713/ajas.2008.70597

64. Černauskienė, J.; Z. Bartkevičiūtė; J. Hammerer; K. Kozłowski; H. Jeroch. The Effect of "Bonvital", a Probiotic Product Containing Enterococcus Faecium on the Fattening Performance, Carcass Characteristics and Meat Quality of Pigs under Production Conditions. *Vet. ir Zootech.* **2010**, 54(76), 20-25.

65. Meng, Q.W.; L. Yan; X. Ao; T.X. Zhou; J.P. Wang; J.H. Lee; I.H. Kim. Influence of Probiotics in Different Energy and Nutrient Density Diets on Growth Performance, Nutrient Digestibility, Meat Quality, and Blood Characteristics in Growing-Finishing Pigs. *J. Anim. Sci.* **2010**, 88(10), 3320-3326. doi:10.2527/jas.2009-2308

66. Giang, H.H.; T.Q. Viet; B. Ogle; J.E. Lindberg. Effects of Supplementation of Probiotics on the Performance, Nutrient Digestibility and Faecal Microflora in Growing-Finishing Pigs. *Asian Australas. J. Anim. Sci.* **2011**, 24(5), 655-661. doi:10.5713/ajas.2011.10238

67. Nitikanchana, S.; M.D. Tokach; J.M. DeRouchey; R.D. Goodband; J.L. Nelssen; S.S. Dritz, *The Effect of Bacillus Probiotic on Growth Performance and Fecal Consistency of Growing-Finishing Pigs*, in *Kansas State University Swine Day*. 2011, Kansas State University: Kansas. p. 240-246.

68. Hossain, M.E.; S.Y. Ko; K.W. Park; J.D. Firman; C.J. Yang. Evaluation of Green Tea by-Product and Green Tea Plus Probiotics on the Growth Performance, Meat Quality and Immunity of Growing-Finishing Pigs. *Anim. Prod. Sci.* **2012**, 52(9), 857-866. doi:10.1071/AN11141

69. Cui, C.; C.J. Shen; G. Jia; K.N. Wang. Effect of Dietary Bacillus Subtilis on Proportion of Bacteroidetes and Firmicutes in Swine Intestine and Lipid Metabolism. *Genet. Mol. Res.* **2013**, 12(2), 1766-1776. doi:10.4238/2013.May.23.1

70. Kerr, B.J.; T.E. Weber; G.C. Shurson. Evaluation of Commercially Available Enzymes, Probiotics, or Yeast on Apparent Total-Tract Nutrient Digestion and Growth in Nursery and Finishing Pigs Fed Diets Containing Corn Dried Distillers Grains with Solubles. *Prof. Anim. Sci.* **2013**, 29(5), 508-517. doi:10.15232/S1080-7446(15)30272-2

71. Liu, T.; B. Su; J. Wang; C. Zhang; A. Shan. Effects of Probiotics on Growth, Pork Quality and Serum Metabolites in Growing-Finishing Pigs. *J. Northeast Agric. Univ.* **2013**, 20(4), 57-63. doi:10.1016/S1006-8104(14)60048-9

72. Balasubramanian, B.; T. Li; I.H. Kim. Effects of Supplementing Growing-Finishing Pig Diets with Bacillus Spp. Probiotic on Growth Performance and Meat-Carcass Grade Quality Traits. *Rev. Bras. Zootec.* **2016**, 45(3), 93-100. doi:10.1590/S1806-92902016000300002

73. Dowarah, R.; A.K. Verma; A. Neeta; S. Putan. Effect of Swine-Based Probiotic on Growth Performance, Nutrient Utilization and Immune Status of Early-Weaned Grower-Finisher Crossbred Pigs. *Anim. Nutr. Feed Technol.* **2016**, 16(3), 451-461. doi:10.5958/0974-181X.2016.00042.1

74. Jørgensen, J.N.; J.S. Laguna; C. Millán; O. Casabuena; M.I. Gracia. Effects of a Bacillus-Based Probiotic and Dietary Energy Content on the Performance and Nutrient Digestibility of Wean to Finish Pigs. *Anim. Feed Sci. Technol.* **2016**, 221, 54-61. doi:10.1016/j.anifeedsci.2016.08.008

75. Sarker, M.S.K.; A.B.M.R. Bostami; G.M. Kim; H. Ji; C. Yang. Potential of Bamboo Vinegar with Liquid Probiotics on Growth Performance, Fecal Microbiology and Fecal Odorous Gas Emissions from Finishing Pigs. *Afr. J. Microbiol. Res.* **2016**, 10(11), 363-369. doi:10.5897/AJMR2016.7936

76. Nguyen, D.H.; K.Y. Lee; H.N. Tran; S.D. Upadhaya; Y.J. Jeong; I.H. Kim. Influence of Enterococcus Faecium and Endo-1,4-B-Xylanase Supplementation on Growth Performance, Nutrient Digestibility, Fecal Microflora, Fecal Gas

Emission, and Meat Quality in Finishing Pigs Fed with Diets Based on Corn–Soybean Meal. *Can. J. Anim. Sci.* **2017**, 98(1), 126-134. doi:10.1139/cjas-2016-0197

77. Tufarelli, V.; A.M. Crovace; G. Rossi; V. Laudadio. Effect of a Dietary Probiotic Blend on Performance, Blood Characteristics, Meat Quality and Faecal Microbial Shedding in Growing-Finishing Pigs. *S. Afr. J. Anim. Sci.* **2017**, 47(6), 875-882. doi:10.4314/sajas.v47i6.15

78. Balasubramanian, B.; S.I. Lee; I.H. Kim. Inclusion of Dietary Multi-Species Probiotic on Growth Performance, Nutrient Digestibility, Meat Quality Traits, Faecal Microbiota and Diarrhoea Score in Growing–Finishing Pigs. *Ital. J. Anim. Sci.* **2018**, 17(1), 100-106. doi:10.1080/1828051X.2017.1340097

79. Bučko, O.; A. Lehotayová; I. Imrich; I. Bahelka. Carcass Characteristics and Meat Quality of Pigs Fed Diets Containing Probiotic Preparation Based on Lactobacillus Plantarum. *Res. Pig Breed.* **2018**, 12(1), 1-4.

80. Samolińska, W.; E. Kowalczuk-Vasilev; E.R. Grela. Comparative Effect of Different Dietary Inulin Sources and Probiotics on Growth Performance and Blood Characteristics in Growing–Finishing Pigs. *Arch. Anim. Nutr.* **2018**, 72(5), 379-395. doi:10.1080/1745039X.2018.1505147

81. Shi, D.; J. Zhou; L. Zhao; X. Rong; Y. Fan; H. Hamid; W. Li; C. Ji; Q. Ma. Alleviation of Mycotoxin Biodegradation Agent on Zearalenone and Deoxynivalenol Toxicosis in Immature Gilts. *J. Anim. Sci. Biotechnol.* **2018**, 9, 42. doi:10.1186/s40104-018-0255-z

82. Lan, R.; I. Kim. Effects of Bacillus Licheniformis and Bacillus Subtilis Complex on Growth Performance and Faecal Noxious Gas Emissions in Growing-Finishing Pigs. *J. Sci. Food Agric.* **2019**, 99(4), 1554-1560. doi:10.1002/jsfa.9333

83. Wang, H.; I. Kim. Influence of the Efficacy of a Probiotic Complex Containing Bacillus Subtilis and Pichia Farinosa on the Growth Performance and Fecal Microbiota of Finishing Pigs. *Can. J. Anim. Sci.* **2019**, 99(4), 966-970. doi:10.1139/cjas-2018-0226

84. Peet-Schwering, C.M.C.v.d.; R. Verheijen; L. Jørgensen; L. Raff. Effects of a Mixture of Bacillus Amyloliquefaciens and Bacillus Subtilis on the Performance of Growing-Finishing Pigs. *Anim. Feed Sci. Technol.* **2020**, 261. doi:10.1016/j.anifeedsci.2020.114409

85. Reszka, P.; A. Dunislawska; A. Slawinska; M. Siwek; W. Kapelański; J. Bogucka. Influence of the Effective Microorganisms (Em) on Performance, Intestinal Morphology and Gene Expression in the Jejunal Mucosa of Pigs Fed Different Diets. *J. Anim. Physiol. Anim. Nutr. (Berl.)* **2020**, 104(5), 1444-1453. doi:10.1111/jpn.13404

86. Rybarczyk, A.; E. Boguslawska-Was; A. Lupkowska. Effect of Em® Probiotic on Gut Microbiota, Growth Performance, Carcass and Meat Quality of Pigs. *Livest. Sci.* **2020**, 241. doi:10.1016/j.livsci.2020.104206

87. Frimpong, Y.O.; M. Boateng; K.O. Amoah; P.Y. Atuahene; S.O. Okungbowa; J. Baah; D.B. Okai. Response of Large White Gilts to Diets Containing Differing Probiotic Products. *Sci. Afr.* **2021**, 13(4). doi:10.1016/j.sciaf.2021.e00878

88. Grela, E.R.; M. Świątkiewicz; M. Florek; M. Bąkowski; G. Skiba. Effect of Inulin Source and a Probiotic Supplement in Pig Diets on Carcass Traits, Meat Quality and Fatty Acid Composition in Finishing Pigs. *Animals* **2021**, 11(8). doi:10.3390/ani11082438

89. Kwak, M.J.; P.L. Tan; J.K. Oh; K.S. Chae; J. Kim; S.H. Kim; J.S. Eun; S.W. Chee; D.K. Kang; S.H. Kim; K.Y. Whang. The Effects of Multispecies Probiotic Formulations on Growth Performance, Hepatic Metabolism, Intestinal Integrity and Fecal Microbiota in Growing-Finishing Pigs. *Anim. Feed Sci. Technol.* **2021**, 274. doi:10.1016/j.anifeedsci.2021.114833

90. Pomorska-Mól, M.; H. Turlewicz-Podbielska; J. Wojciechowski. Effects of the Microencapsulated Feed Additive of Lactic Acid Bacteria on Production Parameters and Post-Vaccinal Immune Response in Pigs. *Pol. J. Vet. Sci.* **2021**, 24(3), 335-343. doi:10.24425/pjvs.2021.137670

91. Rybarczyk, A.; E. Bogusławska-Wąs; A. Dłubała. Effect of Bioplus Yc Probiotic Supplementation on Gut Microbiota, Production Performance, Carcass and Meat Quality of Pigs. *Animals* **2021**, 11(6). doi:10.3390/ani11061581

92. Shen, W.; Y. Liu; X. Zhang; X. Zhang; X. Rong; L. Zhao; C. Ji; Y. Lei; Q. Ma; J. Chen; F. Li. Comparison of Ameliorative Effects between Probiotic and Biodegradable Bacillus Subtilis on Zearalenone Toxicosis in Gilts. *Toxins* **2021**, 13(12). doi:10.3390/toxins13120882

93. Runjun, D.; A.K. Verma; A. Neeta; S. Putan. Efficacy of Species-Specific Probiotic Pediococcus Acidilactici Ft28 on Blood Biochemical Profile, Carcass Traits and Physicochemical Properties of Meat in Fattening Pigs. *Res. Vet. Sci.* **2018**, 117, 60-64. doi:10.1016/j.rvsc.2017.11.011

94. Reszka, P.; D. Cygan-Szczegielniak; H. Jankowiak; A. Cebulska; B. Mikołajczak; J. Bogucka. Effects of Effective Microorganisms on Meat Quality, Microstructure of the Longissimus Lumborum Muscle, and Electrophoretic Protein Separation in Pigs Fed on Different Diets. *Animals* **2020**, 10(10). doi:10.3390/ani10101755

95. Tian, Z.; Y. Cui; H. Lu; G. Wang; X. Ma. Effect of Long-Term Dietary Probiotic Lactobacillus Reuteri 1 or Antibiotics on Meat Quality, Muscular Amino Acids and Fatty Acids in Pigs. *Meat Sci.* **2021**, 171. doi:10.1016/j.meatsci.2020.108234

96. Barber, R.S.; R. Braude; K.G. Mitchell; A.W. Myres. The Value of Hydrocarbon-Grownyeast S a Source of Protein for Growing Pigs. *Br. J. Nutr.* **1971**, 25(2), 285-94. doi:10.1079/bjn19710089

97. Bowman, G.L.; T.L. Veum. Saccharomyces Cerevisiae Yeast Culture in Growing-Finishing Swine Diets. *J. Anim. Sci.* **1973**, 37(1), 72-74.

98. Burnett, G.S.; E.L. Neil. A Note on the Effect of Probioticum Feed Additive on the Live-Weight Gain, Feed Conversion and Carcass Quality of Bacon Pigs. *Anim. Prod.* **1977**, 25(1), 95-98. doi:10.1017/S0003356100039088

99. Bae, K.H.; T.G. Ko; J.H. Kim; W.T. Cho; Y.K. Han; I.K. Han. Use of Metabolically Active Substances to Substitute for Antibiotics in Finishing Pigs. *Korean J. Anim. Sci.* **1999**, 41(1), 23-30.

100. Davis, M.E.; C.V. Maxwell; D.C. Brown; B.Z.d. Rodas; Z.B. Johnson; E.B. Kegley; D.H. Hellwig; R.A. Dvorak. Effect of Dietary Mannan Oligosaccharides and(or) Pharmacological Additions of Copper Sulfate on Growth Performance and Immunocompetence of Weanling and Growing/Finishing Pigs. *J. Anim. Sci.* **2002**, 80(11), 2887-2894. doi:10.2527/2002.80112887x

101. Reynoso-González, E.; M. Cervantes-Ramírez; J.L. Figueroa-Velasco; A. Morales-Trejo; A. Araiza-Piña; J. Yáñez-Hernández. Levels of Protein, Fiber, and Yeast Saccharomyces Cerevisiae in Wheat-Based Diets for Pigs. *Agrociencia* **2010**, 44(7), 753-762.

102. Ha, S.; B. Park; S. Son; D. Ha; C.Y. Lee. Effects of the Low-Crude Protein and Lysine (Low Cp/Lys) Diet and a Yeast Culture Supplemented to the Low Cp/Lys Diet on Growth and Carcass Characteristics in Growing-Finishing Pigs. *J. Anim. Sci. Technol.* **2012**, 54(6), 427-433. doi:10.5187/JAST.2012.54.6.427

103. Wenner, B.A.; H.N. Zerby; D.D. Boler; W.A. Gebreyes; S.J. Moeller. Effect of Mannan Oligosaccharides (Bio-Mos) and Outdoor Access Housing on Pig Growth, Feed Efficiency and Carcass Composition. *J. Anim. Sci.* **2013**, 91(10), 4936-4944. doi:10.2527/jas.2013-6582

104. Edwards, M.V.; A.C. Edwards; P. Millard; A. Kocher. Mannose Rich Fraction of Saccharomyces Cerevisiae Promotes Growth and Enhances Carcass Yield in Commercially Housed Grower-Finisher Pigs. *Anim. Feed Sci. Technol.* **2014**, 197, 227-232. doi:10.1016/j.anifeedsci.2014.08.004

105. Lei, Y.; I.H. Kim. Effect of Phaffia Rhodozyma on Performance, Nutrient Digestibility, Blood Characteristics, and Meat Quality in Finishing Pigs. *J. Anim. Sci.* **2014**, 92(1), 171-176. doi:10.2527/jas.2013-6749

106. Szakacs, A.R.; S. Matei; L. Ștefănuț; Z. Moni; A. Macri. Effects of Pre and Probiotic on Growth Performance and Haematological Parameters in Pigs. *Bull. Univ. Agric. Sci. Vet. Med. Cluj. Napoca.* **2016**, 73(2), 295-300. doi:10.15835/buasvmcn-vm:12166

107. Gong, Y.L.; J.B. Liang; M.F. Jahromi; Y.B. Wu; A.G. Wright; X.D. Liao. Mode of Action of Saccharomyces Cerevisiae in Enteric Methane Mitigation in Pigs. *Animal* **2018**, 12(2), 239-245. doi:10.1017/S1751731117001732

108. Zhang, J.; J. Park; I. Kim. Effect of Supplementation with Brewer's Yeast Hydrolysate on Growth Performance, Nutrients Digestibility, Blood Profiles and Meat Quality in Growing to Finishing Pigs. *Asian Australas. J. Anim. Sci.* **2019**, 32(10), 1565-1572. doi:10.5713/ajas.18.0837

109. Bo, H.X.; H.T. Anh; P.X. Hao; P.T. Tuoi; D.D. Luc. Effects of Replacement of Fish Meal and Soybean Meal by Brewers' Yeast Extract on Growth and Feed Conversion of Landrace X Yorkshire Pigs. *Livest. Res. Rural. Dev.* **2020**, 32(6), 1-7.

110. Dávila-Ramírez, J.L.; M.R. Carvajal-Nolazco; M.J. López-Millanes; H. González-Ríos; H. Celaya-Michel; J. Sosa-Castañeda; S.M. Barrales-Heredia; S.F. Moreno-Salazar; M.A. Barrera-Silva. Effect of Yeast Culture (Saccharomyces Cerevisiae) Supplementation on Growth Performance, Blood Metabolites, Carcass Traits, Quality, and Sensorial Traits of Meat from Pigs under Heat Stress. *Anim. Feed Sci. Technol.* **2020**, 267. doi:10.1016/j.anifeedsci.2020.114573

111. He, W.; Y. Gao; Z. Guo; Z. Yang; X. Wang; H. Liu; H. Sun; B. Shi. Effects of Fermented Wheat Bran and Yeast Culture on Growth Performance, Immunity, and Intestinal Microflora in Growing-Finishing Pigs. *J. Anim. Sci.* **2021**, 99(11). doi:10.1093/jas/skab308

112. Mayorga, E.J.; S.K. Kvidera; E.A. Horst; M. Al-Qaisi; C.S. McCarthy; M.A. Abeyta; S. Lei; T.H. Elsasser; S. Kahl; T.G. Kiros; L.H. Baumgard. Effects of Dietary Live Yeast Supplementation on Growth Performance and Biomarkers of Metabolism and Inflammation in Heat-Stressed and Nutrient-Restricted Pigs. *Transl. Anim. Sci.* **2021**, 5(2). doi:10.1093/tas/txab072

113. Namted, S.; K. Poungpong; W. Loongyai; C. Rakangthong; C. Bunchasak. Improving Growth Performance and Blood Profile by Feeding Autolyzed Yeast to Improve Pork Carcass and Meat Quality. *Anim. Sci. J.* **2021**, 92(1). doi:10.1111/asj.13666

114. Lucas, I.A.M.; A.F.C. Calder. Antibiotics and a High Level of Copper Sulphate in Rations for Growing Bacon Pigs. *J. Agric. Sci.* **1957**, 49, 184-199. doi:10.1017/S0021859600036170

115. King, J.O.L. The Effect of Environmental Temperature on the Response of Growing Pigs to Dietary Supplements of an Antibiotic and Copper Sulphate. *Vet. Rec.* **1960**, 72, 304-306.

116. Wallace, H.D.; J.T. McCall; B. Bass; G.E. Combs. High Level Copper for Growing-Finishing Swine. *J. Anim. Sci.* **1960**, 19, 1153-1163. doi:10.2527/jas1960.1941153x

117. Bellis, D.B. Supplementation of Bacon Pig Rations by Aureomycin and Two Levels of Copper Sulphate. *Anim. Prod.* **1961**, 3(1), 89-95. doi:10.1017/S0003356100033808

118. Lucas, I.A.M.; R.M. Livingstone; I. McDonald. Copper Sulphate as a Growth Stimulant for Pigs: Effect of Level and Purity. *Anim. Prod.* **1961**, 3(2), 111-119. doi:10.1017/S0003356100033869

119. Barber, R.S.; R. Braude; K.G. Mitchell. Copper Sulphate and Molasses Distillers Dried Solubles as Dietary Supplements for Growing Pigs. *Anim. Prod.* **1962**, 4(2), 233-238. doi:10.1017/S0003356100034243

120. Braude, R.; M.J. Townsend; G. Harrington; J.G. Rowell. Effects of Oxytetracycline and Copper Sulphate, Separately and Together, in the Rations of Growing Pigs. *J. Agric. Sci.* **1962**, 58, 251-256. doi:10.1017/S0021859600010212

121. Lucas, I.A.M.; R.M. Livingstone; A.W. Boyne. Copper Sulphate as a Growth Stimulant for Pigs: Effect of Composition of Diet and Level of Protein. *Anim. Prod.* **1962**, 4(2), 177-183. doi:10.1017/S0003356100034176

122. Gipp, W.F.; W.G. Pond; S.E. Smith. Effects of Level of Dietary Copper, Molybdenum, Sulfate and Zinc on Bodyweight Gain, Hemoglobin and Liver Copper Storage of Growing Pigs. *J. Anim. Sci.* **1967**, 26(4), 727-30. doi:10.2527/jas1967.264727x

123. Barber, R.S.; R. Braude; K.G. Mitchell; J.D. Harding; G. Lewis; R.M. Loosmore. The Effects of Feeding Toxic Groundnut Meal to Growing Pigs and Its Interaction with High-Copper Diets. *Br. J. Nutr.* **1968**, 22(4), 535-54. doi:10.1079/bjn19680064

124. Hanrahan, T.J.; J.F. O'Grady. Copper Supplementation of Pig Diets. The Effect of Protein Level and Zinc Supplementation on the Response to Added Copper. *Anim. Prod.* **1968**, 10(4), 423-432. doi:10.1017/S0003356100026441

125. Boyazoglu, P.A.; E.L. Barrett. Effects of High Level Copper Supplementation on Growing-Finishing Pigs. *J. S. Afr. Vet. Med. Assoc.* **1970**, 41(3), 201-204.

126. Barber, R.S.; R. Braude; K.G. Mitchell. Arsanilic Acid, Sodium Salicylate and Bromide Salts as Potential Growth Stimulants for Pigs Receiving Diets with and without Copper Sulphate. *Br. J. Nutr.* **1971**, 25(3), 381-9. doi:10.1079/bjn19710103

127. DeGoey, L.W.; R.C. Wahlstrom; R.J. Emerick. Studies of High Level Copper Supplementation to Rations for Growing Swine. *J. Anim. Sci.* **1971**, 33(1), 52-57. doi:10.2527/jas1971.33152x

128. Kline, R.D.; V.W. Hays; G.L. Cromwell. Effects of Copper, Molybdenum and Sulfate on Performance, Hematology and Copper Stores of Pigs and Lambs. *J. Anim. Sci.* **1971**, 33(4), 771-779. doi:10.2527/jas1971.334771x

129. Kline, R.D.; V.W. Hays; G.L. Cromwell. Related Effects of Copper, Zinc and Iron on Performance, Hematology and Copper Stores of Pigs. *J. Anim. Sci.* **1972**, 34(3), 393-6. doi:10.2527/jas1972.343393x

130. Braude, R.; K. Ryder. Copper Levels in Diets for Growing Pigs. *J. Agric. Sci.* **1973**, 80(3), 489-493. doi:10.1017/S0021859600058135

131. Elliot, J.I.; M.A. Amer. Influence of Level of Copper Supplement and Removal of Supplemental Copper from the Diet on the Performance of Growing-Finishing Pigs and Accumulation of Copper in the Liver. *Can. J. Anim. Sci.* **1973**, 53(1), 133-138. doi:10.4141/cjas73-020

132. Gipp, W.F.; W.G. Pond; E.F. Walker, Jr. Influence of Diet Composition and Mode of Copper Administration on the Response of Growing-Finishing Swine to Supplemental Copper. *J. Anim. Sci.* **1973**, 36(1), 91-99. doi:10.2527/jas1973.36191x

133. Kline, R.D.; M.A. Corzo; V.W. Hays; G.L. Cromwell. Related Effects of Copper, Molybdenum and Sulfide on Performance, Hematology and Copper Stores of Growing Pigs. *J. Anim. Sci.* **1973**, 37(4), 936-941. doi:10.2527/jas1973.374936x

134. NCR-42 Committee on Swine Nutrition. Cooperative Regional Studies with Growing Swine: Effects of Vitamin E and Levels of Supplementary Copper During the Growing-Finishing Period on Gain, Feed Conversion and Tissue Copper Storage in Swine. *J. Anim. Sci.* **1974**, 39(3), 512-20. doi:10.2527/jas1974.393512x

135. Bellis, D.B. A Note on Copper Supplementation of Bacon Pig Diets Based on Fishmeal or on Extracted Soyabean Meal. *Rhod. J. Agric. Res.* **1975**, 13(1), 9-13.

136. Castell, A.G.; R.D. Allen; R.M. Beames; J.M. Bell; R. Belzile; J.P. Bowland; J.I. Elliot; M. Ihnat; E. Larmond; T.M. Mallard; D.T. Spurr; S.C. Stothers; S.B. Wilton; L.G. Young. Copper Supplementation of Canadian Diets for Growing-Finishing Pigs. *Can. J. Anim. Sci.* **1975**, 55(1), 113-134. doi:10.4141/cjas75-014

137. Hansen, V.; S. Bresson. Copper Sulphate as a Feed Additive to Bacon Pigs. *Acta Agric. Scand.* **1975**, 25(1), 30-32.

138. Omole, T.A.; J.O. Ilori; A.O. Leigh. Effects of Dietary Copper and Zinc on Performance Carcass Characteristics and Tissue Copper Stores of Market Pigs. *Malays. Agric. Res.* **1976**, 5(1), 67-73.

139. Barber, R.S.; R. Braude; K.G. Mitchell; R.J. Pittman. The Value of Virginiamycin (Eskalin) as a Feed Additive for Growing Pigs in Diets with or without a High Copper Supplement. *Anim. Prod.* **1978**, 26(2), 151-155. doi:10.1017/S0003356100039568

140. Cromwell, G.L.; V.W. Hays; T.L. Clark. Effects of Copper Sulfate, Copper Sulfide and Sodium Sulfide on Performance and Copper Stores of Pigs. *J. Anim. Sci.* **1978**, 46(3), 692-698. doi:10.2527/jas1978.463692x

141. Pond, W.G.; E.F. Walker Jr; D. Kirtland; T. Rounsaville. Effect of Dietary Ca, Cu and Zn Level on Body Weight Gain and Tissue Mineral Concentrations of Growing Pigs and Rats. *J. Anim. Sci.* **1978**, 47(5), 1128-1134. doi:10.2527/jas1978.4751128x

142. Eisemann, J.H.; W.G. Pond; M.L. Thonney. Effect of Dietary Zinc and Copper on Performance and Tissue Mineral and Cholesterol Concentrations in Swine. *J. Anim. Sci.* **1979**, 48(5), 1123-1128. doi:10.2527/jas1979.4851123x

143. Prince, T.J.; V.W. Hays; G.L. Cromwell. Effects of Copper Sulfate and Ferrous Sulfide on Performance and Liver Copper and Iron Stores of Pigs. *J. Anim. Sci.* **1979**, 49(2), 507-513. doi:10.2527/jas1979.492507x

144. Barber, R.S.; R. Braude; K.G. Mitchell. Copper Supplementation of Isonitrogenous Diets for Growing Pigs Containing White-Fish Meal or Soya Bean Meal as the Protein Supplement. *Anim. Prod.* **1981**, 33(1), 81-86. doi:10.1017/S000335610002523X

145. Ribeiro de Lima, F.; T.S. Stahly; G.L. Cromwell. Effects of Copper, with and without Ferrous Sulfide, and Antibiotics on the Performance of Pigs. *J. Anim. Sci.* **1981**, 52(2), 241-7. doi:10.2527/jas1981.522241x

146. Braude, R.; Z.D. Hosking. Copper in Diets for Growing Pigs. *J. Agric. Sci.* **1982**, 99(2), 365-371. doi:10.1017/S002185960003015X

147. Bradley, B.D.; G. Graber; R.J. Condon; L.T. Frobish. Effects of Graded Levels of Dietary Copper on Copper and Iron Concentrations in Swine Tissues. *J. Anim. Sci.* **1983**, 56(3), 625-30. doi:10.2527/jas1983.563625x

148. Prince, T.J.; V.W. Hays; G.L. Cromwell. Interactive Effects of Dietary Calcium, Phosphorus and Copper on Performance and Liver Stores of Pigs. *J. Anim. Sci.* **1984**, 58(2), 356-61. doi:10.2527/jas1984.582356x

149. Southern, L.L.; T.B. Stewart. Performance and Tissue Copper Concentrations of Control and Ascaris Suum-Infected Pigs Fed Excess Dietary Copper. *J. Parasitol.* **1984**, 70(5), 668-670. doi:10.2307/3281747

150. Rowan, T.G.; T.L.J. Lawrence. Growth, Tissue Deposition and Metabolism Studies in Growing Pigs Given Low Glucosinolate Rapeseed Meal Diets Containing Different Amounts of Copper and Polyethylene Glycol. *J. Agric. Sci.* **1986**, 107(3), 505-513. doi:10.1017/S0021859600069653

151. Astrup, H.N.; T. Matre. Feed Conversion, Pork Fat Softening and Liver Malondialdehyde Reactivity in Pigs Supplemented with Copper. *Norw. J. Agric. Sci.* **1987**, 1(2), 81-86.

152. Lüdke, H.; F. Schöne. Copper and Iodine in Pig Diets with High Glucosinolate Rapeseed Meal. I. Performance and Thyroid Hormone Status of Growing Pigs Fed on a Diet with Rapeseed Meal Treated with Copper Sulphate Solution or Untreated and Supplements of Iodine, Copper or a Quinoxaline Derivative. *Anim. Feed Sci. Technol.* **1988**, 22(1-2), 33-43. doi:10.1016/0377-8401(88)90072-7

153. Schöne, F.; H. Lüdke; A. Hennig; G. Jahreis. Copper and Iodine in Pig Diets with High Glucosinolate Rapeseed Meal. Ii. Influence of Iodine Supplements for Rations with Rapeseed Meal Untreated or Treated with Copper Ions on Performance and Thyroid Hormone Status of Growing Pigs. *Anim. Feed Sci. Technol.* **1988**, 22(1-2), 45-59. doi:10.1016/0377-8401(88)90073-9

154. Ward, T.L.; K.L. Watkins; L.L. Southern; P.G. Hoyt; D.D. French. Interactive Effects of Sodium Zeolite-a and Copper in Growing Swine: Growth, and Bone and Tissue Mineral Concentrations. *J. Anim. Sci.* **1991**, 69(2), 726-733. doi:10.2527/1991.692726x

155. Myer, R.O.; J.W. Lamkey; W.R. Walker; J.H. Brendemuhl; G.E. Combs. Performance and Carcass Characteristics of Swine When Fed Diets Containing Canola Oil and Added Copper to Alter the Unsaturated:Saturated Ratio of Pork Fat. *J. Anim. Sci.* **1992**, 70(5), 1417-1423. doi:10.2527/1992.7051417x

156. Southern, L.L.; K.L. Watkins; D.D. French. Effect of Dietary Sodium Bicarbonate on Growth, Liver Copper Concentration and Incidence of Gastric Ulceration in Pigs Fed Excess Dietary Copper. *Int. J. Vitam. Nutr. Res.* **1993**, 63(1), 45-47.

157. Apgar, G.A.; E.T. Kornegay. Mineral Balance of Finishing Pigs Fed Copper Sulfate or a Copper-Lysine Complex at Growth-Stimulating Levels. *J. Anim. Sci.* **1996**, 74(7), 1594-1600. doi:10.2527/1996.7471594x.

158. Lauridsen, C.; S. Højsgaard; M.T. Sørensen. Influence of Dietary Rapeseed Oil, Vitamin E, and Copper on the Performance and the Antioxidative and Oxidative Status of Pigs. *J. Anim. Sci.* **1999**, 77(4), 906-16. doi:10.2527/1999.774906x

159. Hernández, A.; J.R. Pluske; D.N. D'Souza; B.P. Mullan. Minimum Levels of Inclusion of Copper and Zinc Proteinate Amino Acid Chelates in Growing and Finishing Pig Diets. *Anim. Prod. Sci.* **2009**, 49(4), 340-349. doi:10.1071/EA08237

160. Coble, K.F.; J.M. DeRouchey; M.D. Tokach; S.S. Dritz; B.V. Lawrence; J. Escobar; J.C. Woodworth; R.D. Goodband; N. Boettger, *Effects of Copper Sources (Copper Sulfate and Mintrex Cu) on Growth Performance, Carcass Characteristics, Barn Cleaning, and Economics in Finishing Pigs*, in *Kansas State University Swine Day*. 2014, Kansas State University: Kansas. p. 155-163.

161. Feldpausch, J.A.; R.G. Amachawadi; M.D. Tokach; H.M. Scott; T.G. Nagaraja; S.S. Dritz; R.D. Goodband; J.C. Woodworth; J.M. DeRouchey. Effects of Dietary Copper, Zinc, and Ractopamine Hydrochloride on Finishing Pig

Growth Performance, Carcass Characteristics, and Antimicrobial Susceptibility of Enteric Bacteria. *J. Anim. Sci.* **2016**, 94(8), 3278-3293. doi:10.2527/jas.2016-0340

162. Coble, K.F.; J.M. DeRouchey; M.D. Tokach; S.S. Dritz; R.D. Goodband; J.C. Woodworth; J.L. Usry. The Effects of Copper Source and Concentration on Growth Performance, Carcass Characteristics, and Pen Cleanliness in Finishing Pigs. *J. Anim. Sci.* **2017**, 95(9), 4052-4059. doi:10.2527/jas2017.1624

163. Coble, K.F.; D.D. Burnett; J.M. Derouchey; M.D. Tokach; J.M. Gonzalez; F. Wu; S.S. Dritz; R.D. Goodband; J.C. Woodworth; J.R. Pluske. Effect of Diet Type and Added Copper on Growth Performance, Carcass Characteristics, Energy Digestibility, Gut Morphology, and Mucosal Mrna Expression of Finishing Pigs. *J. Anim. Sci.* **2018**, 96(8), 3288-3301. doi:10.1093/jas/sky196

164. Coble, K.F.; F. Wu; J.M. DeRouchey; M.D. Tokach; S.S. Dritz; R.D. Goodband; J.C. Woodworth; J.L. Usry. Effect of Standardized Ileal Digestible Lysine and Added Copper on Growth Performance, Carcass Characteristics, and Fat Quality of Finishing Pigs. *J. Anim. Sci.* **2018**, 96(8), 3249-3263. doi:10.1093/jas/sky184

165. Carpenter, C.B.; J.C. Woodworth; J.M. Derouchey; M.D. Tokach; R.D. Goodband; S.S. Dritz; F. Wu; Z.J. Rambo. Effects of Increasing Copper from Either Copper Sulfate or Combinations of Copper Sulfate and a Copper-Amino Acid Complex on Finishing Pig Growth Performance and Carcass Characteristics. *Transl. Anim. Sci.* **2019**, 3(4), 1263-1269. doi:10.1093/tas/txz112

166. Seidu, A.; H. Meng; D. Che; H. Jiang; R. Han; B. Zhao; D. Kofi; G. Qin. Effect of Dietary Copper Levels on the Growth Performance and Nutrient Utilization in Fattening Pigs. *Indian J. Anim. Res* **2020**, 54(5), 573-577. doi:10.18805/ijar.B-956

167. Blavi, L.; D. Solà; A. Monteiro; J.F. Pérez; H.H. Stein. Inclusion of Dicopper Oxide Instead of Copper Sulfate in Diets for Growing-Finishing Pigs Results in Greater Final Body Weight and Bone Mineralization, but Reduced Accumulation of Copper in the Liver. *J. Anim. Sci.* **2021**, 99(6). doi:10.1093/jas/skab127

168. Wedekind, K.J.; A.J. Lewis; M.A. Giesemann; P.S. Miller. Bioavailability of Zinc from Inorganic and Organic Sources for Pigs Fed Corn-Soybean Meal Diets. *J. Anim. Sci.* **1994**, 72(10), 2681-9. doi:10.2527/1994.72102681x

169. Rupić, V.; L. Ivandija; S. Luterotti; M. Dominis-Kramarić. Influence of Inorganic and Organic Dietary Zinc on Its Concentration in Blood Serum, Bones and Hair and on Catalytical Activity of Some Serum Enzymes in Pigs. *Acta Vet. Brno* **1997**, 66(2), 75-85. doi:10.2754/avb199766020075

170. Paulk, C.B.; M.D. Tokach; S.S. Dritz; J.M. Gonzalez; J.M. Derouchey; R.D. Goodband, *Effects of Added Zinc During the Grower and/or Finisher Phase on Growth Performance and Carcass Characteristics of Finishing Pigs Fed Diets with or without Ractopamine Hcl.*, in *Kansas State University Swine Day*. 2014, Kansas State University: Kansas. p. 164-171.

171. Holen, J.P.; Z. Rambo; A.M. Hilbrands; L.J. Johnston. Effects of Dietary Zinc Source and Concentration on Performance of Growing-Finishing Pigs Reared with Reduced Floor Space. *Prof. Anim. Sci.* **2018**, 34(2), 133-143. doi:10.15232/pas.2017-01684

172. Cemin, H.S.; C.B. Carpenter; J.C. Woodworth; M.D. Tokach; S.S. Dritz; J.M. DeRouchey; R.D. Goodband; J.L. Usry. Effects of Zinc Source and Level on Growth Performance and Carcass Characteristics of Finishing Pigs. *Transl. Anim. Sci.* **2019**, 3(2), 742-748. doi:10.1093/tas/txz071

173. Cemin, H.S.; J.C. Woodworth; M.D. Tokach; S.S. Dritz; J.M. DeRouchey; R.D. Goodband; J.L. Usry. Effects of Increasing Dietary Zinc on Growth Performance and Carcass Characteristics of Pigs Raised under Commercial Conditions. *Transl. Anim. Sci.* **2019**, 3(2), 731-736. doi:10.1093/tas/txz054

174. Villagómez-Estrada, S.; J.F. Pérez; S. van Kuijk; D. Melo-Durán; R. Karimirad; D. Solà-Oriol. Effects of Two Zinc Supplementation Levels and Two Zinc and Copper Sources with Different Solubility Characteristics on the Growth Performance, Carcass Characteristics and Digestibility of Growing-Finishing Pigs. *J. Anim. Physiol. Anim. Nutr. (Berl.)* **2021**, 105(1), 59-71. doi:10.1111/jpn.13447

175. Natalello, A.; H. Khelil-Arfa; G. Luciano; M. Zoon; R. Menci; M. Scerra; A. Blanchard; F. Mangano; L. Biondi; A. Priolo. Effect of Different Levels of Organic Zinc Supplementation on Pork Quality. *Meat Sci.* **2022**, 186. doi:10.1016/j.meatsci.2021.108731

176. Siljander-Rasi, H.; S. Peuranen; K. Tiihonen; E. Virtanen; H. Kettunen; T. Alaviuhkola; P.H. Simmins. Effect of Equi-Molar Dietary Betaine and Choline Addition on Performance, Carcass Quality and Physiological Parameters of Pigs. *Anim. Sci.* **2003**, 76(1), 55-62. doi:10.1017/s1357729800053315

177. Dunshea, F.R.; D.J. Cadogan; G.G. Partridge. Dietary Betaine and Ractopamine Combine to Increase Lean Tissue Deposition in Finisher Pigs, Particularly Gilts. *Anim. Prod. Sci.* **2009**, 49(1), 65-70. doi:10.1071/EA08014

178. Smith, J.W.; J.L. Nelssen; R.D. Goodband; M.D. Tokach; B.T. Richert; K.Q. Owen; J.R. Bergstrom; S.A. Blum, *The Effects of Supplementing Growing-Finishing Swine Diets with Betaine and (or) Choline on Growth and Carcass Characteristics*, in *Kansas State University Swine Day*. 1994, Kansas State University: Kansas. p. 162-164.

179. Smith, J.W.; K.Q. Owen; K.G. Friesen; J.L. Nelssen; R.D. Goodband; M.D. Tokach; T.T. Lohrmann; S.A. Blum, *The Effects of Supplemental Dietary Carnitine, Betaine, and Chromium Nicotinate on Growth and Carcass Characteristics in Growing-Finishing Swine*, in *Kansas State University Swine Day*. 1994, Kansas State University: Kansas. p. 158-161.

180. Matthews, J.O.; L.L. Southern; J.E. Pontif; A.D. Higbie; T.D. Bidner. Interactive Effects of Betaine, Crude Protein, and Net Energy in Finishing Pigs. *J. Anim. Sci.* **1998**, 76(9), 2444-2455. doi:10.2527/1998.7692444x

181. Øverland, M.; K.A. Rørvik; A. Skrede. Effect of Trimethylamine Oxide and Betaine in Swine Diets on Growth Performance, Carcass Characteristics, Nutrient Digestibility, and Sensory Quality of Pork. *J. Anim. Sci.* **1999**, 77(8), 2143-2153. doi:10.2527/1999.7782143x

182. Matthews, J.O.; L.L. Southern; T.D. Bidner; M.A. Persica. Effects of Betaine, Pen Space, and Slaughter Handling Method on Growth Performance, Carcass Traits, and Pork Quality of Finishing Barrows. *J. Anim. Sci.* **2001**, 79(4), 967-974. doi:10.2527/2001.794967x

183. Matthews, J.O.; L.L. Southern; A.D. Higbie; M.A. Persica; T.D. Bidner. Effects of Betaine on Growth, Carcass Characteristics, Pork Quality, and Plasma Metabolites of Finishing Pigs. *J. Anim. Sci.* **2001**, 79(3), 722-728. doi:10.2527/2001.793722x

184. Young, M.G.; S.S. Dritz; M.D. Tokach; R.D. Goodband; J.L. Nelssen, *The Influence of Dietary Energy Level on the Response to Betaine*, in *Kansas State University Swine Day*. 2001, Kansas State University: Kansas. p. 101-104.

185. Lawrence, B.V.; A.P. Schinckel; O. Adeola; K. Cera. Impact of Betaine on Pig Finishing Performance and Carcass Composition. *J. Anim. Sci.* **2002**, 80(2), 475-482. doi:10.2527/2002.802475x

186. Feng, J.; X. Liu; Y.Z. Wang; Z.R. Xu. Effects of Betaine on Performence, Carcass Characteristics and Hepatic Betaine-Homocysteine Methyltransferase Activity in Finishing Barrows. *Asian Australas. J. Anim. Sci.* **2006**, 19(3), 402-405. doi:10.5713/ajas.2006.402

187. Huang, Q.C.; X.Y. Han; Z.R. Xu; X.Y. Yang; T. Chen; X.T. Zheng. Betaine Suppresses Carnitine Palmitoyltransferase I in Skeletal Muscle but Not in Liver of Finishing Pigs. *Livest. Sci.* **2009**, 126(1-3), 130-135. doi:10.1016/j.livsci.2009.06.015

188. Nakev, J.; T. Popova; V. Vasileva. Influence of Dietary Betaine Supplementation on the Growth Performance and Carcass Characteristics in Male and Female Growing-Finishing Pigs. *Bulg. J. Agric. Sci.* **2009**, 15(3), 263-268.

189. Yang, H.S.; J.I. Lee; S.T. Joo; G.B. Park. Effects of Dietary Glycine Betaine on Growth and Pork Quality of Finishing Pigs. *Asian Australas. J. Anim. Sci.* **2009**, 22(5), 706-711. doi:10.5713/ajas.2009.80645

190. Van Heugten, E., *Improving Production Efficiency and Carcass Weight of Finishing Pigs Housed under Heat Stress Conditions by Heat Abatement with Dietary Betaine*. 2014, North Carolina State University.

191. Madeira, M.S.; C.M. Alfaia; P. Costa; P.A. Lopes; S.V. Martins; J.P. Lemos; O. Moreira; J. Santos-Silva; R.J. Bessa; J.A. Prates. Effect of Betaine and Arginine in Lysine-Deficient Diets on Growth, Carcass Traits, and Pork Quality. *J. Anim. Sci.* **2015**, 93(10), 4721-33. doi:10.2527/jas.2015-9117

192. Wang, L.S.; Z. Shi; R. Gao; B.C. Su; H. Wang; B.M. Shi; A.S. Shan. Effects of Conjugated Linoleic Acid or Betaine on the Growth Performance and Fatty Acid Composition in Backfat and Belly Fat of Finishing Pigs Fed Dried Distillers Grains with Solubles. *Animal* **2015**, 9(4), 569-575. doi:10.1017/S1751731114002699

193. Lothong, M.; K. Tachampa; P. Assavacheep; K. Angkanaporn. Effects of Dietary Betaine Supplementation on Back Fat Thickness and Serum Igf-1 in Late Finishing Pigs. *Thai. J. Vet. Med* **2016**, 46(3), 427-434.

194. Mendoza, S.M.; R.D. Boyd; C.E. Zier-Rush; P.R. Ferket; K.D. Haydon; E.v. Heugten. Effect of Natural Betaine and Ractopamine Hcl on Whole-Body and Carcass Growth in Pigs Housed under High Ambient Temperatures. *J. Anim. Sci.* **2017**, 95(7), 3047-3056. doi:10.2527/jas.2017.1622

195. Groesbeck, C.N.; M.D. Tokach; R.D. Goodband; S.S. Dritz; J.L. Nelssen; K.R. Lawrence; F. Valdez. Influence of Chromium Source on Plasma Non-Esterified Fatty Acid Concentrations in Growing-Finishing Pigs. *Kansas State University Swine Day* **2004**, 154-157.

196. Lindemann, M.D.; C.M. Wood; A.F. Harper; E.T. Kornegay; R.A. Anderson. Dietary Chromium Picolinate Additions Improve Gain:Feed and Carcass Characteristics in Growing-Finishing Pigs and Increase Litter Size in Reproducing Sows. *J. Anim. Sci.* **1995**, 73(2), 457-465. doi:10.2527/1995.732457x

197. O'Quinn, P.R.; J.W. Smith, II; J.L. Nelssen; M.D. Tokach; R.D. Goodband; K.Q. Owen; S.A. Blum, *Effects of Source and Level of Added Chromium on Growth Performance and Carcass Characteristics of Growing-Finishing Pigs*, in *Kansas State University Swine Day*. 1998, Kansas State University: Kansas. p. 166-171.

198. Page, T.G.; L.L. Southern; T.L. Ward; D.L. Thompson Jr. Effect of Chromium Picolinate on Growth and Serum and Carcass Traits of Growing-Finishing Pigs. *J. Anim. Sci.* **1993**, 71(3), 656-662. doi:10.2527/1993.713656x

199. Shelton, J.L.; R.L. Payne; S.L. Johnston; T.D. Bidner; L.L. Southern; R.L. Odgaard; T.G. Page. Effect of Chromium Propionate on Growth, Carcass Traits, Pork Quality, and Plasma Metabolites in Growing-Finishing Pigs. *J. Anim. Sci.* **2003**, 81(10), 2515-2524. doi:10.2527/2003.81102515x.

200. Tian, Y.; L. Gong; J. Xue; J. Cao; L. Zhang. Effects of Graded Levels of Chromium Methionine on Performance, Carcass Traits, Meat Quality, Fatty Acid Profiles of Fat, Tissue Chromium Concentrations, and Antioxidant Status in Growing-Finishing Pigs. *Biol. Trace Elem. Res.* **2015**, 168(1), 110-121. doi:10.1007/s12011-015-0352-1

201. Lindemann, M.D.; G.L. Cromwell; H.J. Monegue; K.W. Purser. Effect of Chromium Source on Tissue Concentration of Chromium in Pigs. *J. Anim. Sci.* **2008**, 86(11), 2971-2978. doi:10.2527/jas.2008-0888

202. Sales, J.; F. Jančík. Effects of Dietary Chromium Supplementation on Performance, Carcass Characteristics, and Meat Quality of Growing-Finishing Swine: A Meta-Analysis. *J. Anim. Sci.* **2011**, 89(12), 4054-4067. doi:10.2527/jas.2010-3495

203. Boleman, S.L.; S.J. Boleman; T.D. Bidner; L.L. Southern; T.L. Ward; J.E. Pontif; M.M. Pike. Effect of Chromium Picolinate on Growth, Body Composition, and Tissue Accretion in Pigs. *J. Anim. Sci.* **1995**, 73(7), 2033-2042. doi:10.2527/1995.7372033x

204. Mooney, K.W.; G.L. Cromwell. Effects of Dietary Chromium Picolinate Supplementation on Growth, Carcass Characteristics, and Accretion Rates of Carcass Tissues in Growing-Finishing Swine. *J. Anim. Sci.* **1995**, 73(11), 3351-3357. doi:10.2527/1995.73113351x

205. Smith, J.W., II; J.L. Nelssen; R.D. Goodband; M.D. Tokach; B.T. Richert; K.Q. Owen; J.R. Bergstrom; W.B. Nessmith, Jr.; S.A. Blum, *The Effects of Supplementing Growing-Finishing Pig Diets with Carnitine and(or) Chromium on Growth and Carcass Characteristics*, in *Kansas State University Swine Day*. 1996, Kansas State University: Kansas. p. 111-115.

206. Kornegay, E.T.; Z. Wang; C.M. Wood; M.D. Lindemann. Supplemental Chromium Picolinate Influences Nitrogen Balance, Dry Matter Digestibility, and Carcass Traits in Growing-Finishing Pigs. *J. Anim. Sci.* **1997**, 75(5), 1319-1323. doi:10.2527/1997.7551319x

207. Min, J.K.; W.Y. Kim; B.J. Chae; I.B. Chung; I.S. Shin; Y.J. Choi; I.K. Han. Effects of Chromium Picolinate (Crp) on Growth Performance, Carcass Characteristics and Serum Traits in Growing-Finishing Pigs. *Asian Australas. J. Anim. Sci.* **1997**, 10(1), 8-14. doi:10.5713/ajas.1997.8

208. Mooney, K.W.; G.L. Cromwell. Efficacy of Chromium Picolinate and Chromium Chloride as Potential Carcass Modifiers in Swine. *J. Anim. Sci.* **1997**, 75(10), 2661-2671. doi:10.2527/1997.75102661x.

209. Ward, T.L.; L.L. Southern; T.D. Bidner. Interactive Effects of Dietary Chromium Tripicolinate and Crude Protein Level in Growing-Finishing Pigs Provided Inadequate and Adequate Pen Space. *J. Anim. Sci.* **1997**, 75(4), 1001-1008. doi:10.2527/1997.7541001x

210. Lien, T.; C. Wu; B. Lin; B. Wang; J. Lu; T. Shiao. Effect of Different Protein and Limiting Amino Acid Levels Coupled with a Supplement of Chromium Picolinate on Lipid Metabolism and Carcass Characteristics of Pigs. *Anim. Sci.* **1998**, 67(3), 601-607. doi:10.1017/S135772980003304X

211. Lemme, A.; G. Wenk; M. Lindemann; G. Bee. Chromium Yeast Affects Growth Performance but Not Whole Carcass Composition of Growing-Finishing Pigs. *Ann. Zootech.* **1999**, 48(6), 457-468. doi:10.1051/animres:19990605

212. Mooney, K.W.; G.L. Cromwell. Efficacy of Chromium Picolinate on Performance and Tissue Accretion in Pigs with Different Lean Gain Potential. *J. Anim. Sci.* **1999**, 77(5), 1188-1198. doi:10.2527/1999.7751188x

213. O'Quinn, P.R.; A.T. Waylan; R.D. Goodband; J.A. Unruh; J.L. Nelssen; J.C. Woodworth; M.D. Tokach; K.Q. Owen, *Effects of Modified Tall Oil, Chromium Nicotinate, and L-Carnitine on Growth Performance and Carcass Characteristics of Growing-Finishing Gilts*, in *Kansas State University Swine Day*. 1999, Kansas State University: Kansas. p. 123-128.

214. Hanczakowska, E.; J. Urbańczyk; M. Świątkiewicz. The Efficiency of Betaine and Organic Compounds of Chromium in Fattening of Pigs with Ad Libitum or Restricted Feeding. *Rocz. Nauk. Zootech.* **1999**, 26(4), 263-274.

215. Matthews, J.O.; L.L. Southern; J.M. Fernandez; J.E. Pontif; T.D. Bidner; R.L. Odgaard. Effect of Chromium Picolinate and Chromium Propionate on Glucose and Insulin Kinetics of Growing Barrows and on Growth and Carcass Traits of Growing-Finishing Barrows. *J. Anim. Sci.* **2001**, 79(8), 2172-2178. doi:10.2527/2001.7982172x.

216. Xi, G.; Z. Xu; S. Wu; S. Chen. Effect of Chromium Picolinate on Growth Performance, Carcass Characteristics, Serum Metabolites and Metabolism of Lipid in Pigs. *Asian Australas. J. Anim. Sci.* **2001**, 14(2), 258-262. doi:10.5713/ajas.2001.258

217. Matthews, J.O.; A.D. Higbie; L.L. Southern; D.F. Coombs; T.D. Bidner; R.L. Odgaard. Effect of Chromium Propionate and Metabolizable Energy on Growth, Carcass Traits, and Pork Quality of Growing-Finishing Pigs. *J. Anim. Sci.* **2003**, 81(1), 191-196. doi:10.2527/2003.811191x.

218. Waylan, A.T.; P.R. O'Quinn; R.D. Goodband; J.A. Unruh; J.L. Nelssen; J.C. Woodworth; M.D. Tokach. Effects of Dietary Additions of Modified Tall Oil, Chromium Nicotinate, and L-Carnitine on Growth Performance, Carcass Characteristics, and Bacon Characteristics of Growing-Finishing Pigs. *Can. J. Anim. Sci.* **2003**, 83(3), 459-467. doi:10.4141/A02-036

219. Wang, M.Q.; Z.R. Xu. Effect of Chromium Nanoparticle on Growth Performance, Carcass Characteristics, Pork Quality and Tissue Chromium in Finishing Pigs. *Asian Australas. J. Anim. Sci.* **2004**, 17(8), 1118-1122. doi:10.5713/ajas.2004.1118

220. Matthews, J.O.; A.C. Guzik; F.M. LeMieux; L.L. Southern; T.D. Bidner. Effects of Chromium Propionate on Growth, Carcass Traits, and Pork Quality of Growing-Finishing Pigs. *J. Anim. Sci.* **2005**, 83(4), 858-862. doi:10.2527/2005.834858x.

221. Amoikon, K.E.; T.L. Ward; L.L. Southern. Effects of Dietary Chromium Tripicolinate and Lysine on Growth Performance, Carcass Traits and Plasma Metabolite Levels in Pigs. *Agron. Afr.* **2006**, 18(1), 75-83. doi:10.4314/aga.v18i1.1681

222. Khajarern, J.; S. Khajarern; H.D. Ashmead; S.D. Ashmead. The Effect of Chromium Bisglycinate-Nicotinamide Chelate Supplementation on Growth and Carcass Quality in Growing and Finishing Pigs. *Int. J. Appl. Res. Vet. Med.* **2006**, 4(3), 193-199.

223. Bergstrom, J.R.; M.D. Tokach; S.S. Dritz; J.L. Nelssen; J.M. DeRouchey; R.D. Goodband. Effects of 200 Ppb Added Chromium from Chromium Propionate on the Growth Performance and Carcass Characteristics of Finishing Pigs. *Kansas Agricultural Experiment Station Research Reports* **2008**, 226-230. doi:10.4148/2378-5977.7061

224. Wang, M.Q.; Y.D. He; Z.R. Xu; W.F. Li. Effects of Chromium Picolinate Supplementation on Growth Hormone Secretion and Pituitary Mrna Expression in Finishing Pigs. *Asian Australas. J. Anim. Sci.* **2008**, 21(7), 1033-1037. doi:10.5713/ajas.2008.70692

225. Jackson, A.R.; S. Powell; S.L. Johnston; J.O. Matthews; T.D. Bidner; F.R. Valdez; L.L. Southern. The Effect of Chromium as Chromium Propionate on Growth Performance, Carcass Traits, Meat Quality, and the Fatty Acid Profile of Fat from Pigs Fed No Supplemented Dietary Fat, Choice White Grease, or Tallow. *J. Anim. Sci.* **2009**, 87(12), 4032-4041. doi:10.2527/jas.2009-2168

226. Park, J.K.; J.Y. Lee; B.J. Chae; S.J. Ohh. Effects of Different Sources of Dietary Chromium on Growth, Blood Profiles and Carcass Traits in Growing-Finishing Pigs. *Asian Australas. J. Anim. Sci.* **2009**, 22(11), 1547-1554. doi:10.5713/ajas.2009.80633

227. Wang, M.Q.; Y.D. He; M.D. Lindemann; Z.G. Jiang. Efficacy of Cr (Iii) Supplementation on Growth, Carcass Composition, Blood Metabolites, and Endocrine Parameters in Finishing Pigs. *Asian Australas. J. Anim. Sci.* **2009**, 22(10), 1414-1419. doi:10.5713/ajas.2009.90111

228. Wang, M.Q.; Z.R. Xu; W.F. Li; Z.G. Jiang. Effect of Chromium Nanocomposite Supplementation on Growth Hormone Pulsatile Secretion and Mrna Expression in Finishing Pigs. *J. Anim. Physiol. Anim. Nutr. (Berl.)* **2009**, 93(4), 520-525. doi:10.1111/j.1439-0396.2008.00836.x

229. Li, Y.S.; N.H. Zhu; P.P. Niu; F.X. Shi; C.L. Hughes; G.X. Tian; R.H. Huang. Effects of Dietary Chromium Methionine on Growth Performance, Carcass Composition, Meat Colour and Expression of the Colour-Related Gene Myoglobin of Growing-Finishing Pigs. *Asian Australas. J. Anim. Sci.* **2013**, 26(7), 1021-1029. doi:10.5713/ajas.2013.13012.

230. Panaite, T.; A. Untea; R.D. Criste; C. Papuc; M. Ropota; N.C. Predescu. Effect of the Dietary Chrome Picolinate Supplements Given to Fattening Pigs on the Quality Parameters of the Pig Leg. *Lucrări Științifice - Universitatea de Științe Agricole și Medicină Veterinară, Seria Zootehnie* **2013**, 59, 66-71.

231. Hung, T.; B.J. Leury; M.A. Sabin; C.L. Collins; F.R. Dunshea. Dietary Nano-Chromium Tripicolinate Increases Feed Intake and Decreases Plasma Cortisol in Finisher Gilts During Summer. *Trop. Anim. Health Prod.* **2014**, 46(8), 1483-1489. doi:10.1007/s11250-014-0673-7

232. Peres, L.M.; A.M. Bridi; C.A.d. Silva; N. Andreo; C.C.P. Barata; J.G.N. Dário. Effect of Supplementing Finishing Pigs with Different Sources of Chromium on Performance and Meat Quality. *Rev. Bras. Zootec.* **2014**, 43(7), 369-375. doi:10.1590/S1516-35982014000700005

233. Wang, M.Q.; C. Wang; Y.J. Du; H. Li; W.J. Tao; S.S. Ye; Y.D. He; S.Y. Chen. Effects of Chromium-Loaded Chitosan Nanoparticles on Growth, Carcass Characteristics, Pork Quality, and Lipid Metabolism in Finishing Pigs. *Livest. Sci.* **2014**, 161, 123-129. doi:10.1016/j.livsci.2013.12.029

234. Li, T.Y.; C.M. Fu; T.F. Lien. Effects of Nanoparticle Chromium on Chromium Absorbability, Growth Performance, Blood Parameters and Carcass Traits of Pigs. *Anim. Prod. Sci.* **2017**, 57(6), 1193-1200. doi:10.1071/AN15142

235. Marcolla, C.S.; D.M. Holanda; S.V. Ferreira; G.C. Rocha; N.V.L. Serão; M.S. Duarte; M.L.T. Abreu; A. Saraiva. Chromium, Cla, and Ractopamine for Finishing Pigs. *J. Anim. Sci.* **2017**, 95(10), 4472-4480. doi:10.2527/jas2017.1753

236. Xu, X.; L. Liu; S. Long; X. Piao; T.L. Ward; F. Ji. Effects of Chromium Methionine Supplementation with Different Sources of Zinc on Growth Performance, Carcass Traits, Meat Quality, Serum Metabolites, Endocrine Parameters, and the Antioxidant Status in Growing-Finishing Pigs. *Biol. Trace Elem. Res.* **2017**, 179(1), 70-78. doi:10.1007/s12011-017-0935-0

237. Jin, C.; Q. Wang; Z. Zhang; Y. Xu; H. Yan; H. Li; C. Gao; X. Wang. Dietary Supplementation with Pioglitazone Hydrochloride and Chromium Methionine Improves Growth Performance, Meat Quality, and Antioxidant Ability in Finishing Pigs. *J. Agric. Food. Chem.* **2018**, 66(17), 4345-4351. doi:10.1021/acs.jafc.8b01176

238. Gebhardt, J.T.; J.C. Woodworth; M.D. Tokach; J.M. Derouchey; R.D. Goodband; J.A. Loughmiller; A.L.P. De Souza; M.J. Rincker; S.S. Dritz. Determining the Influence of Chromium Propionate and Yucca Schidigera on Growth

Performance and Carcass Composition of Pigs Housed in a Commercial Environment. *Transl. Anim. Sci.* **2019**, 3(4), 1275-1285. doi:10.1093/tas/txz117

239. Gebhardt, J.T.; J.C. Woodworth; M.D. Tokach; J.M. DeRouchey; R.D. Goodband; J.A. Loughmiller; A.L.P.d. Souza; S.S. Dritz. Influence of Chromium Propionate Dose and Feeding Regimen on Growth Performance and Carcass Composition of Pigs Housed in a Commercial Environment. *Transl. Anim. Sci.* **2019**, 3(1), 384-392. doi:10.1093/tas/txy104

240. Lien, T.F.; Y.S. Lan. Effects of Nanoparticle Chromium Mixed with Γ-Polyglutamic Acid on the Chromium Bioavailability, Growth Performance, Serum Parameters and Carcass Traits of Pigs. *Anim. Prod. Sci.* **2019**, 59(12), 2222-2229. doi:10.1071/AN18441

241. Mayorga, E.J.; S.K. Kvidera; J.T. Seibert; E.A. Horst; M. Abuajamieh; M. Al-Qaisi; S. Lei; J.W. Ross; C.D. Johnson; B. Kremer; L. Ochoa; R.P. Rhoads; L.H. Baumgard. Effects of Dietary Chromium Propionate on Growth Performance, Metabolism, and Immune Biomarkers in Heat-Stressed Finishing Pigs. *J. Anim. Sci.* **2019**, 97(3), 1185-1197. doi:10.1093/jas/sky484

242. da Silva, J.L.; A.P. Dos Santos; T.V.A. Farias; C. Kiefer; K.M.R. de Souza Nascimento; A. Corassa; S.A. da Silva Alencar; G.P. Rodrigues. Chromium and Energy Restriction as Substitutes for Ractopamine in Finishing Gilts Diet. *Cienc. Rural* **2021**, 52(2). doi:10.1590/0103-8478cr20200736

243. Santos, A.P.; M.D. Tokach; C. Kiefer; R.D. Goodband; J.C. Woodworth; J.M. Derouchey; S.S. Dritz; J.T. Gebhardt. Effects of Dietary Chromium Propionate and Space Allowance on Performance and Carcass Responses of Growing-Finishing Pigs. *Transl. Anim. Sci.* **2021**, 5(3). doi:10.1093/tas/txab112

244. Alencar, S.A.D.S.; D.S. Lima; A.P. Dos Santos; C. Kiefer; K.M.R.S. Nascimento; A. Corassa; J.L. da Silva; T.V.A. Farias. Yeast Chromium and Digestible Lysine Levels in Finishing Pigs Subjected to High Ambient Temperatures. *Cienc. Rural* **2022**, 52(3). doi:10.1590/0103-8478cr20200753

245. Wang, L.; Y. Huang; Y. Wang; T. Shan. Effects of Polyunsaturated Fatty Acids Supplementation on the Meat Quality of Pigs: A Meta-Analysis. *Front. Nutr.* **2021**, 8, 746765. doi:10.3389/fnut.2021.746765

246. Dugan, M.E.R.; J.L. Aalhus; A.L. Schaefer; J.K.G. Kramer. The Effect of Conjugated Linoleic Acid on Fat to Lean Repartitioning and Feed Conversion in Pigs. *Can. J. Anim. Sci.* **1997**, 77(4), 723-725. doi:10.4141/A97-084

247. Ostrowska, E.; M. Muralitharan; R.F. Cross; D.E. Bauman; F.R. Dunshea. Dietary Conjugated Linoleic Acids Increase Lean Tissue and Decrease Fat Deposition in Growing Pigs. *J. Nutr.* **1999**, 129(11), 2037-2042. doi:10.1093/jn/129.11.2037

248. O'Quinn, P.R.; J.L. Nelssen; R.D. Goodband; J.A. Unruh; J.C. Woodworth; J.S. Smith; M.D. Tokach. Effects of Modified Tall Oil Versus a Commercial Source of Conjugated Linoleic Acid and Increasing Levels of Modified Tall Oil on Growth Performance and Carcass Characteristics of Growing-Finishing Pigs. *J. Anim. Sci.* **2000**, 78(9), 2359-2368. doi:10.2527/2000.7892359x.

249. Bee, G. Dietary Conjugated Linoleic Acids Affect Tissue Lipid Composition but Not De Novo Lipogenesis in Finishing Pigs. *Anim. Res.* **2001**, 50(5), 383-399. doi:10.1051/animres:2001114

250. Eggert, J.M.; M.A. Belury; A. Kempa-Steczko; S.E. Mills; A.P. Schinckel. Effects of Conjugated Linoleic Acid on the Belly Firmness and Fatty Acid Composition of Genetically Lean Pigs. *J. Anim. Sci.* **2001**, 79(11), 2866-2872. doi:10.2527/2001.79112866x

251. Dugan, M.E.R.; J.L. Aalhus; K.A. Lien; A.L. Schaefer; J.K.G. Kramer. Effects of Feeding Different Levels of Conjugated Linoleic Acid and Total Oil to Pigs on Live Animal Performance and Carcass Composition. *Can. J. Anim. Sci.* **2001**, 81(4), 505-510. doi:10.4141/A00-101

252. Thiel-Cooper, R.L.; F.C. Parrish Jr; J.C. Sparks; B.R. Wiegand; R.C. Ewan. Conjugated Linoleic Acid Changes Swine Performance and Carcass Composition. *J. Anim. Sci.* **2001**, 79(7), 1821-1828. doi:10.2527/2001.7971821x

253. Wiegand, B.R.; F.C. Parrishx Jr; J.E. Swan; S.T. Larsen; T.J. Baas. Conjugated Linoleic Acid Improves Feed Efficiency, Decreases Subcutaneous Fat, and Improves Certain Aspects of Meat Quality in Stress-Genotype Pigs. *J. Anim. Sci.* **2001**, 79(8), 2187-2195. doi:10.2527/2001.7982187x

254. Barowicz, T.; M. Pieszka; M. Pietras; W. Migdał; W. Kędzior. Conjugated Linoleic Acid Utilization for Improvement of Chemical Composition and Dietetic Value of Pork Meat. *Ann. Anim. Sci.* **2002**, 2(2), 123-130.

255. Dunshea, F.R.; E. Ostrowska; B. Luxford; R.J. Smits; R.G. Campbell; D.N. D'Souza; B.P. Mullan. Dietary Conjugated Linoleic Acid Can Decrease Backfat in Pigs Housed under Commercial Conditions. *Asian Australas. J. Anim. Sci.* **2002**, 15(7), 1011-1017. doi:10.5713/ajas.2002.1011

256. Tischendorf, F.; F. Schöne; U. Kirchheim; G. Jahreis. Influence of a Conjugated Linoleic Acid Mixture on Growth, Organ Weights, Carcass Traits and Meat Quality in Growing Pigs. *J. Anim. Physiol. Anim. Nutr. (Berl.)* **2002**, 86(3/4), 117-128. doi:10.1046/j.1439-0396.2002.00366.x

257. Wiegand, B.R.; J.C. Sparks; F.C. Parrish Jr; D.R. Zimmerman. Duration of Feeding Conjugated Linoleic Acid Influences Growth Performance, Carcass Traits, and Meat Quality of Finishing Barrows. *J. Anim. Sci.* **2002**, 80(3), 637-643. doi:10.2527/2002.803637x

258. Ostrowska, E.; D. Suster; M. Muralitharan; R.F. Cross; B.J. Leury; D.E. Bauman; F.R. Dunshea. Conjugated Linoleic Acid Decreases Fat Accretion in Pigs: Evaluation by Dual-Energy X-Ray Absorptiometry. *Br. J. Nutr.* **2003**, 89(2), 219-229. doi:10.1079/BJN2002765

259. Sun, D.; X. Zhu; S. Qiao; S. Fan; D. Li. Effects of Conjugated Linoleic Acid Levels and Feeding Intervals on Performance, Carcass Traits and Fatty Acid Composition of Finishing Barrows. *Arch. Anim. Nutr.* **2004**, 58(4), 277-286. doi:10.1080/00039420412331273286

260. Barowicz, T.; M. Pieszka; M.P. Pietras; W. Migdal. Influence of Dietary Conjugated Linoleic Acid on Lipid Metabolism and Serum Leptin Concentrations in Finishing Pigs. *Biotechnol. Anim. Husb.* **2005**, 21(5/6), 119-122. doi:10.2298/BAH0502119B

261. Lauridsen, C.; H. Mu; P. Henckel. Influence of Dietary Conjugated Linoleic Acid (Cla) and Age at Slaughtering on Performance, Slaughter- and Meat Quality, Lipoproteins, and Tissue Deposition of Cla in Barrows. *Meat Sci.* **2005**, 69(3), 393-399. doi:10.1016/j.meatsci.2004.08.009

262. Weber, T.E.; B.T. Richert; M.A. Belury; Y. Gu; K. Enright; A.P. Schinckel. Evaluation of the Effects of Dietary Fat, Conjugated Linoleic Acid, and Ractopamine on Growth Performance, Pork Quality, and Fatty Acid Profiles in Genetically Lean Gilts. *J. Anim. Sci.* **2006**, 84(3), 720-732. doi:10.2527/2006.843720x.

263. Bee, G.; S. Jacot; G. Guex; C. Biolley. Effects of Two Supplementation Levels of Linseed Combined with Cla or Tallow on Meat Quality Traits and Fatty Acid Profile of Adipose and Different Muscle Tissues in Slaughter Pigs. *Animal* **2008**, 2(5), 800-811. doi:10.1017/S175173110800181X

264. Corino, C.; M. Musella; G. Pastorelli; R. Rossi; K. Paolone; L. Costanza; A. Manchisi; G. Maiorano. Influences of Dietary Conjugated Linoleic Acid (Cla) and Total Lysine Content on Growth, Carcass Characteristics and Meat Quality of Heavy Pigs. *Meat Sci.* **2008**, 79(2), 307-316. doi:10.1016/j.meatsci.2007.10.001

265. Martin, D.; E. Muriel; E. Gonzalez; J. Viguera; J. Ruiz. Effect of Dietary Conjugated Linoleic Acid and Monounsaturated Fatty Acids on Productive, Carcass and Meat Quality Traits of Pigs. *Livest. Sci.* **2008**, 117(2/3), 155-164. doi:10.1016/j.livsci.2007.12.005

266. White, H.M.; B.T. Richert; J.S. Radcliffe; A.P. Schinckel; J.R. Burgess; S.L. Koser; S.S. Donkin; M.A. Latour. Feeding Conjugated Linoleic Acid Partially Recovers Carcass Quality in Pigs Fed Dried Corn Distillers Grains with Solubles. *J. Anim. Sci.* **2009**, 87(1), 157-166. doi:10.2527/jas.2007-0734

267. Jiang, Z.Y.; W.J. Zhong; C.T. Zheng; Y.C. Lin; L. Yang; S.Q. Jiang. Conjugated Linoleic Acid Differentially Regulates Fat Deposition in Backfat and Longissimus Muscle of Finishing Pigs. *J. Anim. Sci.* **2010**, 88(5), 1694-1705. doi:10.2527/jas.2008-1551

268. Han, X.F.; F.J. Feng; J.P. Yu; S.X. Tang; M.A. Bamikole; Z.L. Tan; B. Zeng; C.S. Zhou; M. Wang. Effects of Conjugated Linoleic Acid Supplementation on Growth, Carcass Characteristics and Fatty Acid Profiles of Muscle and Fat in Growing-Finishing Pigs. *J. Anim. Feed Sci.* **2011**, 20(2), 171-185. doi:10.22358/jafs/66169/2011

269. Lee, J.; K. Cha; B. Chae; S. Ohh. Supplementation of Either Conjugated Linoleic Acid or Γ-Linolenic Acid with or without Carnitine to Pig Diet Affect Flavor of Pork and Neutrophil Phagocytosis. *J. Anim. Sci. Technol.* **2011**, 53(3), 237-252. doi:10.5187/JAST.2011.53.3.237

270. Barnes, K.M.; N.R. Winslow; A.G. Shelton; K.C. Hlusko; M.J. Azain. Effect of Dietary Conjugated Linoleic Acid on Marbling and Intramuscular Adipocytes in Pork. *J. Anim. Sci.* **2012**, 90(4), 1142-1149. doi:10.2527/jas.2011-4642

271. Go, G.W.; G.Y. Wu; D.T. Silvey; S.H. Choi; X.L. Li; S.B. Smith. Lipid Metabolism in Pigs Fed Supplemental Conjugated Linoleic Acid and/or Dietary Arginine. *Amino Acids* **2012**, 43(4), 1713-1726. doi:10.1007/s00726-012-1255-5

272. Martinez-Aispuro, M.; J.L. Figueroa-Velasco; V. Zamora-Zamora; M.T. Sanchez-Torres; M.E. Ortega-Cerrilla; J.L. Cordero-Mora; A. Ruiz-Flores; S.S. Gonzalez-Muñoz. Effect of Fatty Acids Source on Growth Performance, Carcass Characteristics, Plasma Urea Nitrogen Concentration, and Fatty Acid Profile in Meat of Pigs Fed Standard- or Low-Protein Diets. *Span. J. Agric. Res* **2012**, 10(4), 993-1004. doi:10.5424/sjar/2012104-323-11

273. Rickard, J.W.; B.R. Wiegand; D. Pompeu; R.B. Hinson; G.D. Gerlemann; R. Disselhorst; M.E. Briscoe; H.L. Evans; G.L. Allee. The Effect of Corn Distiller's Dried Grains with Solubles, Ractopamine, and Conjugated Linoleic Acid on the Carcass Performance, Meat Quality, and Shelf-Life Characteristics of Fresh Pork Following Three Different Storage Methods. *Meat Sci.* **2012**, 90(3), 643-652. doi:10.1016/j.meatsci.2011.10.007

274. Pompeu, D.; B.R. Wiegand; H.L. Evans; J.W. Rickard; G.D. Gerlemann; R.B. Hinson; S.N. Carr; M.J. Ritter; R.D. Boyd; G.L. Allee. Effect of Corn Dried Distillers Grains with Solubles, Conjugated Linoleic Acid, and Racto-Pamine (Paylean) on Growth Performance and Fat Characteristics of Late Finishing Pigs. *J. Anim. Sci.* **2013**, 91(2), 793-803. doi:10.2527/jas.2012-5257

275. Tous, N.; R. Lizardo; B. Vilà; M. Gispert; M. Font-i-Furnols; E. Esteve-Garcia. Effect of a High Dose of Cla in Finishing Pig Diets on Fat Deposition and Fatty Acid Composition in Intramuscular Fat and Other Fat Depots. *Meat Sci.* **2013**, 93(3), 517-524. doi:10.1016/j.meatsci.2012.10.005

276. Martínez-Aispuro, M.; J.L. Figueroa-Velasco; V. Zamora-Zamora; J.L. Cordero-Mora; C. Narciso-Gaytán; M.T. Sánchez-Torres; S. Carrillo-Domínguez; R.M. Castillo-Domínguez. Effect of Cla Supplementation to Low-Protein Diets on the Growth Performance, Carcass Characteristics, Plasma Urea Nitrogen Concentration, and Fatty Acid Profile in the Meat of Pigs. *Braz. Arch. Biol. Technol.* **2014**, 57(5). doi:10.1590/S1516-8913201401407

277. Upadhaya, S.D.; H. Yun; S. Huang; I. Kim. Efficacy of Dietary Supplementation of Fatty Acid Compound on Performance and Production in Finishing Pigs. *Trop. Anim. Health Prod.* **2017**, 49(6), 1281-1288. doi:10.1007/s11250-017-1326-4

278. Panisson, J.C.; A. Maiorka; S.G. Oliveira; A. Saraiva; M.S. Duarte; K.F. Silva; E.V. Santos; R.L.S. Tolentino; I.M.G. Lopes; L.L.M. Guedes; B.A.N. Silva. Effect of Ractopamine and Conjugated Linoleic Acid on Performance of Late Finishing Pigs. *Animal* **2020**, 14(2), 277-284. doi:10.1017/S1751731119001708

279. Averette Gatlin, L.; M.T. See; D.K. Larick; X. Lin; J. Odle. Conjugated Linoleic Acid in Combination with Supplemental Dietary Fat Alters Pork Fat Quality. *J. Nutr.* **2002**, 132(10), 3105-3112. doi:10.1093/jn/131.10.3105

280. Corino, C.; S. Magni; G. Pastorelli; R. Rossi; J. Mourot. Effect of Conjugated Linoleic Acid on Meat Quality, Lipid Metabolism, and Sensory Characteristics of Dry-Cured Hams from Heavy Pigs. *J. Anim. Sci.* **2003**, 81(9), 2219-2229. doi:10.2527/2003.8192219x.

281. Corino, C.; A. Di Giancamillo; R. Rossi; C. Domeneghini. Dietary Conjugated Linoleic Acid Affects Morphofunctional and Chemical Aspects of Subcutaneous Adipose Tissue in Heavy Pigs. *J. Nutr.* **2005**, 135(6), 1444-50. doi:10.1093/jn/135.6.1444

282. Ostrowska, E.; R.F. Cross; R.D. Warner; M. Muralitharan; D.E. Bauman; F.R. Dunshea. Dietary Conjugated Linoleic Acid Improves Carcass Leanness without Altering Meat Quality in the Growing Pig. *Aust. J. Exp. Agric.* **2005**, 45(6), 691-697. doi:10.1071/EA04144

283. Rossi, R.; G. Pastorelli; M. Musella; C. Corino. Influence of Dietary Conjugated Linoleic Acid (Cla) and L-Lysine on Heavy Pigs Performances and Meat Quality. *Ital. J. Anim. Sci.* **2005**, 4(Supplement 2), 464-466. doi:10.4081/ijas.2005.2s.464

284. Cechova, M.; Z. Hadas; R. Beckova; L. Sladek; M. Rychetska. Changes in the Fattening Capacity and Carcass Value of Pigs Receiving the Cla Preparation. *Res. Pig Breed.* **2009**, 3.

285. Larsen, S.T.; B.R. Wiegand; F.C. Parrish Jr; J.E. Swan; J.C. Sparks. Dietary Conjugated Linoleic Acid Changes Belly and Bacon Quality from Pigs Fed Varied Lipid Sources. *J. Anim. Sci.* **2009**, 87(1), 285-295. doi:10.2527/jas.2008-1213

286. Cechova, M.; R. Beckova; Z. Hadas; E. Vaclavkova; M. Rychetska. Effect of Cla and Sunflower Oil in Pig Diet on Carcass Value Traits and Meat Quality. *Res. Pig Breed.* **2010**, 4(1).

287. Cordero, G.; B. Isabel; D. Menoyo; A. Daza; J. Morales; C. Piñeiro; C.J. Lopez-Bote. Dietary Cla Supplementation and Gender Modify Fatty Acid Composition of Subcutaneous and Intramuscular Fat in Iberian × Duroc Finishing Heavy Pigs. *Span. J. Agric. Res* **2010**, 8(4), 962-970. doi:10.5424/sjar/2010084-1244

288. Cordero, G.; B. Isabel; D. Menoyo; A. Daza; J. Morales; C. Piñeiro; C.J. López-Bote. Dietary Cla Alters Intramuscular Fat and Fatty Acid Composition of Pig Skeletal Muscle and Subcutaneous Adipose Tissue. *Meat Sci.* **2010**, 85(2), 235-239. doi:10.1016/j.meatsci.2010.01.004

289. Bothma, C.; A. Hugo; G. Osthoff; C.C. Joubert; J.C. Swarts; H.L.d. Kock. Effect of Dietary Conjugated Linoleic Acid Supplementation on the Technological Quality of Backfat of Pigs. *Meat Sci.* **2014**, 97(2), 277-286. doi:10.1016/j.meatsci.2014.02.002

290. James, B.W.; M.D. Tokach; R.D. Goodband; J.L. Nelssen; S.S. Dritz; K.Q. Owen; J.C. Woodworth; R.C. Sulabo. Interactive Effects of Dietary Ractopamine Hcl and L-Carnitine on Finishing Pigs: I. Growth Performance. *J. Anim. Sci.* **2013**, 91(7), 3265-3271. doi:10.2527/jas.2011-4286

291. Meng, Q.; S. Sun; Y. Sun; J. Li; D. Wu; A. Shan; B. Shi; B. Cheng. Effects of Dietary Lecithin and L-Carnitine on Fatty Acid Composition and Lipid-Metabolic Genes Expression in Subcutaneous Fat and Longissimus Thoracis of Growing-Finishing Pigs. *Meat Sci.* **2018**, 136, 68-78. doi:10.1016/j.meatsci.2017.10.012

292. Ying, W.; M.D. Tokach; J.M. DeRouchey; T.E. Houser; S.S. Dritz; R.D. Goodband; J.L. Nelssen. Effects of Dietary L-Carnitine and Dried Distillers Grains with Solubles on Growth, Carcass Characteristics, and Loin and Fat Quality of Growing-Finishing Pigs. *J. Anim. Sci.* **2013**, 91(7), 3211-3219. doi:10.2527/jas.2012-5606

293. Ringseis, R.; J. Keller; K. Eder. Basic Mechanisms of the Regulation of L-Carnitine Status in Monogastrics and Efficacy of L-Carnitine as a Feed Additive in Pigs and Poultry. *J. Anim. Physiol. Anim. Nutr. (Berl.)* **2018**, 102(6), 1686-1719. doi:10.1111/jpn.12959

294. Owen, K.Q.; H. Jit; C.V. Maxwell; J.L. Nelssen; R.D. Goodband; M.D. Tokach; G.C. Tremblay; S.I. Koo. Dietary L-Carnitine Suppresses Mitochondrial Branched-Chain Keto Acid Dehydrogenase Activity and Enhances Protein Accretion and Carcass Characteristics of Swine. *J. Anim. Sci.* **2001**, 79(12), 3104-12. doi:10.2527/2001.79123104x

295. Owen, K.Q.; J.L. Nelssen; R.D. Goodband; M.D. Tokach; K.G. Friesen. Effect of Dietary L-Carnitine on Growth Performance and Body Composition in Nursery and Growing-Finishing Pigs. *J. Anim. Sci.* **2001**, 79(6), 1509-1515. doi:10.2527/2001.7961509x

296. Bertol, T.M.; M. Ellis; D.N. Hamilton; M.J. Ritter. Effects of Dietary Supplementation with L-Carnitine and Fat on Blood Acid-Base Responses to Handling in Slaughter Weight Pigs. *J. Anim. Sci.* **2005**, 83(1), 75-81. doi:10.2527/2005.83175x

297. Han, Y.K.; P.A. Thacker. Effects of L-Carnitine, Selenium-Enriched Teast, Jujube Fruit and Hwangto (Red Clay) Supplementation on Performance and Carcass Measurements of Finishing Pigs. *Asian Australas. J. Anim. Sci.* **2006**, 19(2), 217-223. doi:10.5713/ajas.2006.217

298. Chen, Y.J.; I.H. Kim; J.H. Cho; J.S. Yoo; Q. Wang; Y. Wang; Y. Huang. Evaluation of Dietary L-Carnitine or Garlic Powder on Growth Performance, Dry Matter and Nitrogen Digestibilities, Blood Profiles and Meat Quality in Finishing Pigs. *Anim. Feed Sci. Technol.* **2008**, 141(1/2), 141-152. doi:10.1016/j.anifeedsci.2007.05.025

299. Pietruszka, A.; E. Jacyno; A. Kolodziej; M. Kawçcka; C. Elzanowski; B. Matysiak. Effects of L-Carnitine and Iron Diet Supplementations on Growth Performance, Carcass Characteristics and Blood Metabolites in Fattening Pigs. *Agric. Food Sci.* **2009**, 18(1), 27-34. doi:10.2137/145960609788066816

300. James, B.W.; M.D. Tokach; R.D. Goodband; J.L. Nelssen; S.S. Dritz; K.Q. Owen; J.C. Woodworth; R.C. Sulabo. Effects of Dietary L-Carnitine and Ractopamine Hcl on the Metabolic Response to Handling in Finishing Pigs. *J. Anim. Sci.* **2013**, 91(9), 4426-4439. doi:10.2527/jas.2011-4411

301. James, B.W.; M.D. Tokach; R.D. Goodband; J.L. Nelssen; S.S. Dritz; K.Q. Owen; J.C. Woodworth; R.C. Sulabo. Interactive Effects of Dietary Ractopamine Hcl and L-Carnitine on Finishing Pigs: Ii. Carcass Characteristics and Meat Quality. *J. Anim. Sci.* **2013**, 91(7), 3272-3282. doi:10.2527/jas.2011-4287

302. Aranda-Aguirre, E.; L.E. Robles-Jimenez; J. Osorio-Avalos; E. Vargas-Bello-Pérez; M. Gonzalez-Ronquillo. A Systematic-Review on the Role of Exogenous Enzymes on the Productive Performance at Weaning, Growing and Finishing in Pigs. *Vet. Anim. Sci.* **2021**, 14, 100195. doi:10.1016/j.vas.2021.100195

303. Kiarie, E.G.; S. Steelman; M. Martinez; K. Livingston. Significance of Single Β-Mannanase Supplementation on Performance and Energy Utilization in Broiler Chickens, Laying Hens, Turkeys, Sows, and Nursery-Finish Pigs: A Meta-Analysis and Systematic Review. *Transl. Anim. Sci.* **2021**, 5(4), txab160. doi:10.1093/tas/txab160

304. Thacker, P.A.; G.L. Campbell; J.W.D. GrootWassink. The Effect of Beta-Glucanase Supplementation on the Performance of Pigs Fed Hulless Barley. *Nutr. Rep. Int.* **1988**, 38(1), 91-99.

305. Baas, T.C.; P.A. Thacker. Impact of Gastric Ph on Dietary Enzyme Activity and Survivability in Swine Fed Β-Glucanase Supplemented Diets. *Can. J. Anim. Sci.* **1996**, 76(2), 245-252. doi:10.4141/cjas96-03

306. Flis, M.; W. Sobotka; Z. Zduńczyk. Replacement of Soyabean Meal by White Lupin Cv. Bardo Seeds and the Effectiveness of Β-Glucanase and Xylanase in Growing-Finishing Pig Diets. *J. Anim. Feed Sci.* **1998**, 7(3), 301-312. doi:10.22358/jafs/69305/1998

307. O'Doherty, J.V.; S. Forde. The Effect of Protease and Α-Galactosidase Supplementation on the Nutritive Value of Peas for Growing and Finishing Pigs. *Irish J. Agric. Food Res.* **1999**, 38(2), 217-226.

308. Thacker, P.A.; G.L. Campbell. Performance of Growing/Finishing Pigs Fed Untreated or Micronized Hulless Barley-Based Diets with or without B-Glucanase. *J. Anim. Feed Sci.* **1999**, 8(2), 157-170. doi:10.22358/jafs/68833/1999

309. Mavromichalis, I.; J.D. Hancock; B.W. Senne; T.L. Gugle; G.A. Kennedy; R.H. Hines; C.L. Wyatt. Enzyme Supplementation and Particle Size of Wheat in Diets for Nursery and Finishing Pigs. *J. Anim. Sci.* **2000**, 78(12), 3086-3095. doi:10.2527/2000.78123086x.

310. Grandhi, R.R. Effect of Dietary Ideal Amino Acid Ratios, and Supplemental Carbohydrase in Hulless-Barley-Based Diets on Pig Performance and Nitrogen Excretion in Manure. *Can. J. Anim. Sci.* **2001**, 81(1), 125-132. doi:10.4141/A00-064

311. Pettey, L.A.; S.D. Carter; B.W. Senne; J.A. Shriver. Effects of Beta-Mannanase Addition to Corn-Soybean Meal Diets on Growth Performance, Carcass Traits, and Nutrient Digestibility of Weanling and Growing-Finishing Pigs. *J. Anim. Sci.* **2002**, 80(4), 1012-9. doi:10.2527/2002.8041012x

312. Park, J.S.; I.H. Kim; J.D. Hancock; R.H. Hines; C. Cobb; H. Cao; J.W. Hong; O.S. Kwon. Effects of Amylase and Cellulase Supplementation in Sorghum-Based Diets for Finishing Pigs. *Asian Australas. J. Anim. Sci.* **2003**, 16(1), 70-76. doi:10.5713/ajas.2003.70

313. Park, J.S.; I.H. Kim; J.D. Hancock; C.L. Wyatt; K.C. Behnke; G.A. Kennedy. Effects of Expander Processing and Enzyme Supplementation of Wheat-Based Diets for Finishing Pigs. *Asian Australas. J. Anim. Sci.* **2003**, 16(2), 248-256. doi:10.5713/ajas.2003.248

314. Flis, M.; W. Sobotka. Fine Particle Size and Enzyme Supplementation as Factors Improving Utilization of Protein from Diets with Lowered Protein Contents by Pigs. *J. Anim. Feed Sci.* **2005**, 14(Suppl.1), 341-344. doi:10.22358/jafs/70574/2005

315. Thacker, P.A. Effect of Xylanase and Protease on the Performance of Growing-Finishing Pigs Fed Corn-Based Diets. *J. Appl. Anim. Res.* **2005**, 28(1), 17-23. doi:10.1080/09712119.2005.9706781

316. Thacker, P.A.; B.G. Rossnagel. Effect of Enzyme Supplementation on the Performance of Growing-Finishing Pigs Fed Diets Containing Normal or High Fat Oats. *J. Anim. Vet. Adv.* **2005**, 4(4), 484-490. doi:javaa.2005.484.490

317. Thacker, P.A.; B.G. Rossnagel. Performance of Growing-Finishing Pigs Fed Diets Containing Normal or Low Lignin-High Fat Oat Supplemented or Unsupplemented with Enzyme. *J. Anim. Vet. Adv.* **2005**, 4(7), 681-687. doi:javaa.2005.681.687

318. Kim, S.W.; J.H. Zhang; K.T. Soltwedel; D.A. Knabe. Use of Carbohydrases in Corn-Soybean Meal Based Grower-Finisher Pig Diets. *Anim. Res.* **2006**, 55(6), 563-578. doi:10.1051/animres:2006039

319. Roșu, M.; C. Falcă. The Effect of Swine Intake Supplementation with Biozyme X 1000 Upon Some Sanguine and Bioproductive Parameters. *Bulletin of University of Agricultural Sciences and Veterinary Medicine Cluj-Napoca. Veterinary Medicine* **2007**, 64(1/2), 283-286.

320. Świątkiewicz, M.; E. Hanczakowska. Effect of Herbs Mixture and Enzymes Supplementation of a Grass Silage Diet for Pigs on Performance and Meat Quality. *Med. Weter.* **2008**, 64(6), 782-785.

321. Wang, J.P.; S.M. Hong; L. Yan; J.S. Yoo; J.H. Lee; H.D. Jang; H.J. Kim; I.H. Kim. Effects of Single or Carbohydrases Cocktail in Low-Nutrient-Density Diets on Growth Performance, Nutrient Digestibility, Blood Characteristics, and Carcass Traits in Growing-Finishing Pigs. *Livest. Sci.* **2009**, 126(1/3), 215-220. doi:10.1016/j.livsci.2009.07.003

322. Widyaratne, G.P.; J.F. Patience; R.T. Zijlstra. Effect of Xylanase Supplementation of Diets Containing Wheat Distiller's Dried Grains with Solubles on Energy, Amino Acid and Phosphorus Digestibility and Growth Performance of Grower-Finisher Pigs. *Can. J. Anim. Sci.* **2009**, 89(1), 91-95. doi:10.4141/CJAS08103

323. Jacela, J.Y.; S.S. Dritz; J.M. DeRouchey; M.D. Tokach; R.D. Goodband; J.L. Nelssen. Effects of Supplemental Enzymes in Diets Containing Distillers Dried Grains with Solubles on Finishing Pig Growth Performance. *Prof. Anim. Sci.* **2010**, 26(4), 412-424. doi:10.15232/S1080-7446(15)30623-9

324. Yoon, S.Y.; Y.X. Yang; P.L. Shinde; J.Y. Choi; J.S. Kim; Y.W. Kim; K. Yun; J.K. Jo; J.H. Lee; S.J. Ohh; I.K. Kwon; B.J. Chae. Effects of Mannanase and Distillers Dried Grain with Solubles on Growth Performance, Nutrient Digestibility, and Carcass Characteristics of Grower-Finisher Pigs. *J. Anim. Sci.* **2010**, 88(1), 181-191. doi:10.2527/jas.2008-1741

325. Barnes, J.A.; J.M. DeRouchey; M.D. Tokach; R.D. Goodband; S.S. Dritz; J.L. Nelssen. Effects of Xylanase in Growing-Finishing Diets Varying in Dietary Energy and Fiber on Growth Performance, Carcass Characteristics, and Nutrient Digestibility. *Kansas Agricultural Experiment Station Research Reports* **2011**, 0(10), 227-239. doi:10.4148/2378-5977.7124

326. Hanczakowska, E.; M. Świątkiewicz; I. Kühn. Efficiency and Dose Response of Xylanase in Diets for Fattening Pigs. *Ann. Anim. Sci.* **2012**, 12(4), 539-548. doi:10.2478/v10220-012-0045-z

327. Jo, J.K.; S.L. Ingale; J.S. Kim; Y.W. Kim; K.H. Kim; J.D. Lohakare; J.H. Lee; B.J. Chae. Effects of Exogenous Enzyme Supplementation to Corn- and Soybean Meal-Based or Complex Diets on Growth Performance, Nutrient Digestibility, and Blood Metabolites in Growing Pigs. *J. Anim. Sci.* **2012**, 90(9), 3041-3048. doi:10.2527/jas.2010-3430

328. McAlpine, P.O.; C.J. O'Shea; P.F. Varley; J.V. O'Doherty. The Effect of Protease and Xylanase Enzymes on Growth Performance and Nutrient Digestibility in Finisher Pigs. *J. Anim. Sci.* **2012**, 90(Suppl. 4), 375-377. doi:10.2527/jas.53979

329. Cho, J.H.; I.H. Kim. Effects of Beta Mannanase and Xylanase Supplementation in Low Energy Density Diets on Performances, Nutrient Digestibility, Blood Profiles and Meat Quality in Finishing Pigs. *Asian J. Anim. Vet. Adv.* **2013**, 8(4), 622-630. doi:10.3923/ajava.2013.622.630

330. Kim, K.; J. Cho; I. Kim. Effects of Dietary Carbohydrases on Growth Performance, Nutrient Digestibility and Blood Characteristics in Finishing Pigs. *J. Anim. Sci. Technol.* **2013**, 55(4), 289-293. doi:10.5187/JAST.2013.55.4.289

331. Lipiński, K.; H. Skórko-Sajko; C. Purwin; Z. Antoszkiewicz; M. Werpachowski. Effect of Xylanase Supplementation to Cereal-Based Diets on Apparent Fecal Digestibility and Growth Performance of Pigs. *Ann. Anim. Sci.* **2013**, 13(2), 303-311. doi:10.2478/aoas-2013-0011

332. O'Shea, C.J.; P.O. Mc Alpine; P. Solan; T. Curran; P.F. Varley; A.M. Walsh; J.V.O. Doherty. The Effect of Protease and Xylanase Enzymes on Growth Performance, Nutrient Digestibility, and Manure Odour in Grower-Finisher Pigs. *Anim. Feed Sci. Technol.* **2014**, 189, 88-97. doi:10.1016/j.anifeedsci.2013.11.012

333. Villca, B.; R. Lizardo; J. Broz; J. Brufau; D. Torrallardona. Effect of a Carbohydrase Enzyme Complex on the Nutrient Apparent Total Tract Digestibility of Rye-Based Diets Fed to Growing-Finishing Pigs under Liquid Feeding. *J. Anim. Sci.* **2016**, 94(7), 230-233. doi:10.2527/jas2015-9747

334. Lindemann, M., *An Integrated Evaluation of the Nutrient Uplift Provided by Xylanase in Finishing*. 2016, University of Kentucky.

335. Nguyen, D.H.; K.Y. Lee; H.N. Tran; S.D. Upadhaya; Y.J. Jeong; I.H. Kim. Influence of Enterococcus Faecium and Endo-1,4-Β-Xylanase Supplementation on Growth Performance, Nutrient Digestibility, Fecal Microflora, Fecal Gas Emission, and Meat Quality in Finishing Pigs Fed with Diets Based on Corn-Soybean Meal. *Can. J. Anim. Sci.* **2018**, 98(1), 126-134. doi:10.1139/cjas-2016-0197

336. Torres-Pitarch, A.; U.M. McCormack; V.E. Beattie; E. Magowan; G.E. Gardiner; A.M. Pérez-Vendrell; D. Torrallardona; J.V. O'Doherty; P.G. Lawlor. Effect of Phytase, Carbohydrase, and Protease Addition to a Wheat Distillers Dried Grains with Solubles and Rapeseed Based Diet on in Vitro Ileal Digestibility, Growth, and Bone Mineral Density of Grower-Finisher Pigs. *Livest. Sci.* **2018**, 216, 94-99. doi:10.1016/j.livsci.2018.07.003

337. Smit, M.N.; X. Zhou; J.L. Landero; M.G. Young; E. Beltranena. Increasing Hybrid Rye Level Substituting Wheat Grain with or without Enzyme on Growth Performance and Carcass Traits of Growing-Finishing Barrows and Gilts. *Transl. Anim. Sci.* **2019**, 3(4), 1561-1574. doi:10.1093/tas/txz141

338. Jang, J.C.; D.H. Kim; J.S. Hong; Y.D. Jang; Y.Y. Kim. Effects of Copra Meal Inclusion Level in Growing- Finishing Pig Diets Containing Β-Mannanase on Growth Performance, Apparent Total Tract Digestibility, Blood Urea Nitrogen Concentrations and Pork Quality. *Animals* **2020**, 10(10), 1-12. doi:10.3390/ani10101840

339. Jang, J.C.; K.H. Kim; D.H. Kim; S.K. Jang; J.S. Hong; P.S. Heo; Y.Y. Kim. Effects of Increasing Levels of Palm Kernel Meal Containing Β-Mannanase to Growing-Finishing Pig Diets on Growth Performance, Nutrient Digestibility, and Pork Quality. *Livest. Sci.* **2020**, 238. doi:10.1016/j.livsci.2020.104041

340. Torres-Pitarch, A.; G.E. Gardiner; P. Cormican; M. Rea; F. Crispie; J.V. O'Doherty; P. Cozannet; T. Ryan; J. Cullen; P.G. Lawlor. Effect of Cereal Fermentation and Carbohydrase Supplementation on Growth, Nutrient Digestibility and Intestinal Microbiota in Liquid-Fed Grow-Finishing Pigs. *Sci. Rep.* **2020**, 10(1), 13716. doi:10.1038/s41598-020-70443-x

341. Torres-Pitarch, A.; G.E. Gardiner; P. Cormican; M. Rea; F. Crispie; J.V. O'Doherty; P. Cozannet; T. Ryan; P.G. Lawlor. Effect of Cereal Soaking and Carbohydrase Supplementation on Growth, Nutrient Digestibility and Intestinal Microbiota in Liquid-Fed Grow-Finishing Pigs. *Sci Rep* **2020**, 10(1), 1023. doi:10.1038/s41598-020-57668-6

342. Kpogo, A.L.; J. Jose; J.C. Panisson; A.K. Agyekum; B.Z. Predicala; A.C. Alvarado; J.M. Agnew; C.J. Sprenger; A.D. Beaulieu. Greenhouse Gases and Performance of Growing Pigs Fed Wheat-Based Diets Containing Wheat Millrun and a Multi-Carbohydrase Enzyme. *J. Anim. Sci.* **2021**, 99(10). doi:10.1093/jas/skab213

343. Reyna, L.; J.L. Figueroa; V. Zamora; J.L. Cordero; M.T. Sánchez-Torres; M. Cuca. Addition of Protease to Standard Diet or Low Protein, Amino Acid Supplemented, Sorghum-Soyabean Meal Diets for Growing-Finishing Pigs. *J. Anim. Vet. Adv.* **2006**, 5(12), 1202-1208. doi:javaa.2006.1202.1208

344. Stephenson, E.W.; J.M. DeRouchey; J. Escobar; J.C. Woodworth; M.D. Tokach; R.D. Goodband; S.S. Dritz, *Effects of a Novel Protease Enzyme (Cibenza Dp100) on Finishing Pig Growth Performance and Carcass Characteristics*, in *Kansas State University Swine Day*. 2014, Kansas State University: Kansas. p. 69-76.

345. Upadhaya, S.D.; H.M. Yun; I.H. Kim. Influence of Low or High Density Corn and Soybean Meal-Based Diets and Protease Supplementation on Growth Performance, Apparent Digestibility, Blood Characteristics and Noxious Gas Emission of Finishing Pigs. *Anim. Feed Sci. Technol.* **2016**, 216, 281-287. doi:10.1016/j.anifeedsci.2016.04.003

346. Choe, J.; K.S. Kim; H.B. Kim; S. Park; J. Kim; S. Kim; B. Kim; S.H. Cho; J.Y. Cho; I.H. Park; J.H. Cho; M. Song. Effects of Protease on Growth Performance and Carcass Characteristics of Growing-Finishing Pigs. *S. Afr. J. Anim. Sci.* **2017**, 47(5), 697-703. doi:10.4314/sajas.v47i5.13

347. Lei, X.; J. Cheong; J. Park; I. Kim. Supplementation of Protease, Alone and in Combination with Fructooligosaccharide to Low Protein Diet for Finishing Pigs. *Anim. Sci. J.* **2017**, 88(12), 1987-1993. doi:10.1111/asj.12849

348. Nguyen, D.H.; S.I. Lee; J.Y. Cheong; I.H. Kim. Influence of Low-Protein Diets and Protease and Bromelain Supplementation on Growth Performance, Nutrient Digestibility, Blood Urine Nitrogen, Creatinine, and Faecal Noxious Gas in Growing–Finishing Pigs. *Can. J. Anim. Sci.* **2018**, 98(3), 488-497. doi:10.1139/cjas-2016-0116

349. Figueroa, J.L.; J.A. Martinez; M.T. Sanchez-Torres; J.L. Cordero; M. Martinez; V.M. Valdez; A. Ruiz. Evaluation of Reduced Amino Acids Diets Added with Protected Protease on Productive Performance in 25-100 Kg Barrows. *Austral J. Vet. Sci.* **2019**, 51(2), 53-60. doi:10.4067/S0719-81322019000200053

350. Liu, X.; J. Yin; I. Kim. Effect of Protease Derived from Pseudoalteromonas Arctica Supplementation on Growth Performance, Nutrient Digestibility, Meat Quality, Noxious Gas Emission and Blood Profiles in Finishing Pigs. *J. Anim. Physiol. Anim. Nutr. (Berl.)* **2019**, 103(6), 1926-1933. doi:10.1111/jpn.13202

351. Min, Y.; Y. Choi; Y. Kim; Y. Jeong; D. Kim; J. Kim; H. Jung; M. Song. Effects of Protease Supplementation on Growth Performance, Blood Constituents, and Carcass Characteristics of Growing-Finishing Pigs. *J. Anim. Sci. Technol.* **2019**, 61(4), 234-238. doi:10.5187/jast.2019.61.4.234

352. Lee, J.; J. Choe; J. Kang; J. Cho; S. Park; R. Perez-Maldonado; J. Cho; I. Park; H. Kim; M. Song. Dietary Protease Improves Growth Rate and Protein Digestibility of Growing-Finishing Pigs. *J. Anim. Sci. Technol.* **2020**, 62(3), 313-320. doi:10.5187/jast.2020.62.3.313

353. Kim, Y.J.; J.H. Lee; T.H. Kim; M.H. Song; W. Yun; H.J. Oh; J.S. Lee; H.B. Kim; J.H. Cho. Effect of Low Protein Diets Added with Protease on Growth Performance, Nutrient Digestibility of Weaned Piglets and Growingfinishing Pigs. *J. Anim. Sci. Technol.* **2021**, 63(3), 491-500. doi:10.5187/jast.2021.e49

354. Perez-Palencia, J.Y.; R.S. Samuel; C.L. Levesque. Supplementation of Protease to Low Amino Acid Diets Containing Superdose Level of Phytase for Wean-to-Finish Pigs: Effects on Performance, Postweaning Intestinal Health and Carcass Characteristics. *Transl. Anim. Sci.* **2021**, 5(2). doi:10.1093/tas/txab088

355. Cromwell, G.L.; T.S. Stahly; R.D. Coffey; H.J. Monegue; J.H. Randolph. Efficacy of Phytase in Improving the Bioavailability of Phosphorus in Soybean Meal and Corn-Soybean Meal Diets for Pigs. *J. Anim. Sci.* **1993**, 71(7), 1831-1840. doi:10.2527/1993.7171831x

356. Cromwell, G.L.; R.D. Coffey; H.J. Monegue; J.H. Randolph. Efficacy of Low-Activity, Microbial Phytase in Improving the Bioavailability of Phosphorus in Corn-Soybean Meal Diets for Pigs. *J. Anim. Sci.* **1995**, 73(2), 449-456. doi:10.2527/1995.732449x

357. Helander, E.; K. Partanen. Effects of Phosphorus Level and Microbial Phytase Supplementation on the Performance and Bone Mineralization in Pigs. *Acta Agric. Scand.* **1997**, 47(3), 159-167. doi:10.1080/09064709709362382

358. O'Doherty, J.V.; S. Forde; J.J. Callan. The Use of Microbial Phytase in Grower and Finisher Pig Diets. *Irish J. Agric. Food Res.* **1999**, 38(2), 227-239.

359. Gebert, S.; G. Bee; H.P. Pfirter; C. Wenk. Growth Performance and Nutrient Utilisation as Influenced in Pigs by Microbial Phytase and Vitamin E Supplementation to a Diet of High Oxidative Capacity. *Ann. Zootech.* **1999**, 48(2), 105-115. doi:10.1051/animres:19990203

360. Gagné, F.; J.J. Matte; G. Barnett; C. Pomar. The Effect of Microbial Phytase and Feed Restriction on Protein, Fat and Ash Deposition in Growing-Finishing Pigs. *Can. J. Anim. Sci.* **2002**, 82(4), 551-558. doi:10.4141/A01-076

361. Brady, S.M.; J.J. Callan; D. Cowan; M. McGrane; J.V. O'Doherty. Effect of Phytase Inclusion and Calcium/Phosphorus Ratio on the Performance and Nutrient Retention of Grower-Finisher Pigs Fed Barley/Wheat/Soya Bean Meal-Based Diets. *J. Sci. Food Agric.* **2002**, 82(15), 1780-1790. doi:10.1002/jsfa.1262

362. Thacker, P.A.; B.G. Rossnagel. Performance and Carcass Traits of Finishing Pigs Fed Low Phosphorus Containing Diets Based on Normal Hulled or Hulless Barley of a Low-Phytate Hulless Barley with and without Phytase. *J. Anim. Vet. Adv.* **2006**, 5(5), 401-407. doi:javaa.2006.401.407

363. Thacker, P.A.; B.G. Rossnagel; V. Raboy. The Effects of Phytase Supplementation on Nutrient Digestibility, Plasma Parameters, Performance and Carcass Traits of Pigs Fed Diets Based on Low-Phytate Barley without Inorganic Phosphorus. *Can. J. Anim. Sci.* **2006**, 86(2), 245-254.

364. Varley, P.F.; J.J. Callan; J.V. O'Doherty. Effect of Phosphorus Level and Phytase Inclusion on the Performance, Bone Mineral Concentration, Apparent Nutrient Digestibility, and on Mineral and Nitrogen Utilization in Finisher Pigs. *Irish J. Agric. Food Res.* **2010**, 49(2), 141-152. doi:10.2307/41219179

365. Langbein, K.B.; J.C. Woodworth; R.D. Goodband; M.D. Tokach; J.L. Nelssen; S.S. Dritz; J.M. DeRouchey. Effects of Super-Dosing Phytase in Diets with Adequate Phosphorus on Finishing Pig Growth Performance and Carcass Characteristics. *Kansas Agricultural Experiment Station Research Reports* **2013**, 0(10), 128-131. doi:10.4148/2378-5977.7044

366. Patience, J., *Improving Nutrient Utilization and Biological and Financial Performance through the Use of Super-Dosing of Phytase in Grow-Finish Diets*. 2015, Iowa State University.

367. Pérez Alvarado, M.A.; D.A. Calderón Montañez; D. Braña Varela; J.A. Cuarón Ibargüengoytia. A Study of the Strategies of Amino Acid Value Assignment to Phytases. *Rev. Mex. Cienc. Pecu.* **2015**, 6(3), 305-314.

368. Holloway, C.L.; R. Dean Boyd; D. Koehler; S.A. Gould; Q. Li; J.F. Patience. The Impact of "Super-Dosing" Phytase in Pig Diets on Growth Performance During the Nursery and Grow-out Periods. *Transl. Anim. Sci.* **2019**, 3(1), 419-428. doi:10.1093/tas/txy148

369. Dang, D.X.; I.H. Kim. Effects of Adding High-Dosing Aspergillus Oryzae Phytase to Corn–Wheat–Soybean Meal-Based Basal Diet on Growth Performance, Nutrient Digestibility, Faecal Gas Emission, Carcass Traits and Meat Quality in Growing-Finishing Pigs. *J. Anim. Physiol. Anim. Nutr. (Berl.)* **2021**, 105(6), 1056-1062. doi:10.1111/jpn.13537

370. Dang, d.X.; I.H. Kim. Effects of Supplementation of High-Dosing Trichoderma Reesei Phytase in the Corn-Wheat-Soybean Meal-Based Diets on Growth Performance, Nutrient Digestibility, Carcass Traits, Faecal Gas Emission, and Meat Quality in Growing-Finishing Pigs. *J. Anim. Physiol. Anim. Nutr. (Berl.)* **2021**, 105(3), 485-492. doi:10.1111/jpn.13499

371. Domaćinović, M.; Z. Steiner; Đ. Senčić; Z. Antunovic; P. Mijić. Individual and Combined Usage of Enzyme Preparation and Heat-Treated Cereals in Pig Fattening. *Czech J. Anim. Sci.* **2006**, 51(4). doi:10.17221/3923-CJAS

372. Feoli, C.; J.D. Hancock; T.L. Gugle; S.D. Carter; N.A. Cole, *Effects of Adding Enzymes to Diets with Corn- and Sorghum-Based Dried Distillers Grains with Solubles on Growth Performance and Nutrient Digestibility in Nursery and Finishing Pigs*, in *Kansas State University Swine Day*. 2008, Kansas State University: Kansas. p. 104-110.

373. Benz, J.M.; J.L. Nelssen; J.M. DeRouchey; M.D. Tokach; R.D. Goodband; S.S. Dritz. Effects of an Enzyme Blend (Livestock Answer) in Diets Containing Dried Distillers Grains with Solubles on Growth Performance of Nursery and Finishing Pigs. *Kansas Agricultural Experiment Station Research Reports* **2009**, 0(10), 213-219. doi:10.4148/2378-5977.6795

374. Thacker, P.A. Effects of Supplementary Threonine, Canola Oil or Enzyme on Nutrient Digestibility, Performance and Carcass Traits of Growing-Finishing Pigs Fed Diets Containing Wheat Distillers Grains with Solubles. *Asian Australas. J. Anim. Sci.* **2009**, 22(12), 1676-1685. doi:10.5713/ajas.2009.90295

375. Ao, X.; T.X. Zhou; Q.W. Meng; J.H. Lee; H.D. Jang; J.H. Cho; I.H. Kim. Effects of a Carbohydrase Cocktail Supplementation on the Growth Performance, Nutrient Digestibility, Blood Profiles and Meat Quality in Finishing Pigs Fed Palm Kernel Meal. *Livest. Sci.* **2011**, 137(1-3), 238-243. doi:10.1016/j.livsci.2010.11.014

376. Lee, S.; H. Jung; K. Cho; J. Park; I. Kim; P. Seong; Y. Song. Effects of Corn Dried Distiller's Grains with Solubles and Enzyme Premix Supplements on Growth Performance, Carcass Characteristics and Meat Quality Parameters in Finishing Pigs. *Anim. Sci. J.* **2011**, 82(3), 461-467. doi:10.1111/j.1740-0929.2010.00848.x

377. Sitanaka, N.Y.; F.E.L. Budiño; S.R. De Oliveira; A.D.C.V. Boas; J.E. De Moraes. Enzyme Complex Supplementation on the Performance of Swine in Growth and Finishing Phases. *Rev. Caatinga* **2018**, 31(3), 748-758. doi:10.1590/1983-21252018v31n325rc

378. Lawlor, P.G.; P. Cozannet; W.F. Ryan; P.B. Lynch. Effect of a Combination Phytase and Carbohydrolase Enzyme Supplement on Growth Performance and Bone Mineralization of Pigs from Six Weeks to Slaughter at 105 Kg. *Livest. Sci.* **2019**, 223, 144-150. doi:10.1016/j.livsci.2019.01.028

379. Balasubramanian, B.; J.H. Park; S. Shanmugam; I.H. Kim. Influences of Enzyme Blend Supplementation on Growth Performance, Nutrient Digestibility, Fecal Microbiota and Meat-Quality in Grower-Finisher Pigs. *Animals* **2020**, 10(3), 386. doi:10.3390/ani10030386

380. Coelho, D.; J. Pestana; J.M. Almeida; C.M. Alfaia; C.M.G.A. Fontes; O. Moreira; J.A.M. Prates. A High Dietary Incorporation Level of Chlorella Vulgaris Improves the Nutritional Value of Pork Fat without Impairing the Performance of Finishing Pigs. *Animals* **2020**, 10(12), 1-18. doi:10.3390/ani10122384

381. Jerez-Bogota, K.; C. Sánchez; J. Ibagon; M. Jlali; P. Cozannet; A. Preynat; T.A. Woyengo. Growth Performance and Nutrient Digestibility of Growing and Finishing Pigs Fed Multienzyme-Supplemented Low-Energy and -Amino Acid Diets. *Transl. Anim. Sci.* **2020**, 4(2), 602-615. doi:10.1093/tas/txaa040

382. Huang, Y.K.; L. Zhao; H. Sun; X.M. Xu; J. Maamer; A. Preynat; L.H. Sun; D.S. Qi. A Multicarbohydrase and Phytase Complex Is Able to Compensate a Nutrient-Deficiency in Growing-Finishing Pigs. *Animals* **2021**, 11(4). doi:10.3390/ani11041129

383. Pettey, L.A.; S.D. Carter; B.W. Senne; J.A. Shriver. Effects of Beta-Mannanase Addition to Corn-Soybean Meal Diets on Growth Performance, Carcass Traits, and Nutrient Digestibility of Weanling and Growing-Finishing Pigs. *J Anim Sci* **2002**, 80(4), 1012-9. doi:10.2527/2002.8041012x

384. Pauly, C.; P. Spring; D. Gahan; J.V. Odoherty. The Effect of Cereal Type and Enzyme Supplementation on Carcass Characteristics, Volatile Fatty Acids and Intestinal Microflora and Boar Taint in Entire Male Pigs. *Animal* **2011**, 5(3), 378-386. doi:10.1017/S1751731110001849
